# The Long-Term Health Consequences of Child Physical Abuse, Emotional Abuse, and Neglect: A Systematic Review and Meta-Analysis

**DOI:** 10.1371/journal.pmed.1001349

**Published:** 2012-11-27

**Authors:** Rosana E. Norman, Munkhtsetseg Byambaa, Rumna De, Alexander Butchart, James Scott, Theo Vos

**Affiliations:** 1 Queensland Children's Medical Research Institute, University of Queensland, Herston, Queensland, Australia; 2 School of Population Health, University of Queensland, Herston, Queensland, Australia; 3 Department of Violence and Injury Prevention and Disability, Noncommunicable Diseases and Mental Health, World Health Organization, Geneva, Switzerland; 4 Queensland Centre for Mental Health Research, The Park Centre for Mental Health, Wacol, Queensland, Australia; 5 Metro North Mental Health, Royal Brisbane and Women's Hospital, Herston, Queensland, Australia; 6 The University of Queensland Centre for Clinical Research, Herston, Queensland, Australia; Stellenbosch University, South Africa

## Abstract

**Background:**

Child sexual abuse is considered a modifiable risk factor for mental disorders across the life course. However the long-term consequences of other forms of child maltreatment have not yet been systematically examined. The aim of this study was to summarise the evidence relating to the possible relationship between child physical abuse, emotional abuse, and neglect, and subsequent mental and physical health outcomes.

**Methods and Findings:**

A systematic review was conducted using the Medline, EMBASE, and PsycINFO electronic databases up to 26 June 2012. Published cohort, cross-sectional, and case-control studies that examined non-sexual child maltreatment as a risk factor for loss of health were included. All meta-analyses were based on quality-effects models. Out of 285 articles assessed for eligibility, 124 studies satisfied the pre-determined inclusion criteria for meta-analysis. Statistically significant associations were observed between physical abuse, emotional abuse, and neglect and depressive disorders (physical abuse [odds ratio (OR) = 1.54; 95% CI 1.16–2.04], emotional abuse [OR = 3.06; 95% CI 2.43–3.85], and neglect [OR = 2.11; 95% CI 1.61–2.77]); drug use (physical abuse [OR = 1.92; 95% CI 1.67–2.20], emotional abuse [OR = 1.41; 95% CI 1.11–1.79], and neglect [OR = 1.36; 95% CI 1.21–1.54]); suicide attempts (physical abuse [OR = 3.40; 95% CI 2.17–5.32], emotional abuse [OR = 3.37; 95% CI 2.44–4.67], and neglect [OR = 1.95; 95% CI 1.13–3.37]); and sexually transmitted infections and risky sexual behaviour (physical abuse [OR = 1.78; 95% CI 1.50–2.10], emotional abuse [OR = 1.75; 95% CI 1.49–2.04], and neglect [OR = 1.57; 95% CI 1.39–1.78]). Evidence for causality was assessed using Bradford Hill criteria. While suggestive evidence exists for a relationship between maltreatment and chronic diseases and lifestyle risk factors, more research is required to confirm these relationships.

**Conclusions:**

This overview of the evidence suggests a causal relationship between non-sexual child maltreatment and a range of mental disorders, drug use, suicide attempts, sexually transmitted infections, and risky sexual behaviour. All forms of child maltreatment should be considered important risks to health with a sizeable impact on major contributors to the burden of disease in all parts of the world. The awareness of the serious long-term consequences of child maltreatment should encourage better identification of those at risk and the development of effective interventions to protect children from violence.

*Please see later in the article for the Editors' Summary*

## Abstract 2

Rosana Norman and colleagues conduct a systematic review and meta-analysis to assess the relationship between child physical abuse, emotional abuse, and neglect, and subsequent mental and physical health outcomes.

## Editors' Summary

**Background:**

Child maltreatment—the abuse and neglect of children—is a global problem. There are four types of child maltreatment—sexual abuse (the involvement of a child in sexual activity that he or she does not understand, is unable to give consent to, or is not developmentally prepared for), physical abuse (the use of physical force that harms the child's health, survival, development, or dignity), emotional abuse (the failure to provide a supportive environment by, for example, verbally threatening the child), and neglect (the failure to provide for all aspects of the child's well-being). Most child maltreatment is perpetrated by parents or parental guardians, many of whom were maltreated themselves as children. Other risk factors for parents abusing their children include poverty, mental health problems, and alcohol and drug misuse. Although there is considerable uncertainty about the frequency and severity of child maltreatment, according to the World Health Organization (WHO) about 20% of women and 5%–10% of men report being sexually abused as children, and the prevalence of physical abuse in childhood may be 25%–50%.

**Why Was This Study Done?:**

Child maltreatment has a large public health impact. Sometimes this impact is immediate and direct (injuries and deaths), but, more often, it is long-term, affecting emotional development and overall health. For child sexual abuse, the relationship between abuse and mental disorders in adult life is well-established. Exposure to other forms of child maltreatment has also been associated with a wide range of psychological and behavioral problems, but the health consequences of physical abuse, emotional abuse, and neglect have not been systematically examined. A better understanding of the long-term health effects of child maltreatment is needed to inform maltreatment prevention strategies and to improve treatment for children who have been abused or neglected. In this systematic review and meta-analysis, the researchers quantify the association between exposure to physical abuse, emotional abuse, and neglect in childhood and mental health and physical health outcomes in later life. A systematic review uses predefined criteria to identify all the research on a given topic; a meta-analysis is a statistical approach that combines the results of several studies.

**What Did the Researchers Do and Find?:**

The researchers identified 124 studies that investigated the relationship between child physical abuse, emotional abuse, or neglect and various health outcomes. Their meta-analysis of data from these studies provides suggestive evidence that child physical abuse, emotional abuse, and neglect are causally linked to mental and physical health outcomes. For example, emotionally abused individuals had a three-fold higher risk of developing a depressive disorder than non-abused individuals (an odds ratio [OR] of 3.06). Physically abused and neglected individuals also had a higher risk of developing a depressive disorder than non-abused individuals (ORs of 1.54 and 2.11, respectively). Other mental health disorders associated with child physical abuse, emotional abuse, or neglect included anxiety disorders, drug abuse, and suicidal behavior. Individuals who had been non-sexually maltreated as children also had a higher risk of sexually transmitted diseases and/or risky sexual behavior than non-maltreated individuals. Finally, there was weak and inconsistent evidence that child maltreatment increased the risk of chronic diseases and lifestyle risk factors such as smoking.

**What Do These Findings Mean?:**

By providing suggestive evidence of a causal link between non-sexual child maltreatment and mental health disorders, drug use, suicide attempts, and sexually transmitted diseases and risky sexual behavior, these findings contribute to our understanding of the non-injury health impacts of child maltreatment. Although most of the studies included in the meta-analysis were undertaken in high-income countries, the findings suggest that this link occurs in both high- and low-to-middle-income countries. They also suggest that neglect may be as harmful as physical and emotional abuse. However, they need to be interpreted carefully because of the limitations of this meta-analysis, which include the possibility that children who have been abused may share other, unrecognized factors that are actually the cause of their later mental health problems. Importantly, this confirmation that physical abuse, emotional abuse, and neglect in childhood are important risk factors for a range of health problems draws attention to the need to develop evidence-based strategies for preventing child maltreatment both to reduce childhood suffering and to alleviate an important risk factor for later health problems.

**Additional Information:**

Please access these websites via the online version of this summary at http://dx.doi.org/10.1371/journal.pmed.1001349.

- The World Health Organization provides information on child maltreatment and its prevention (in several languages); Preventing Child Maltreatment: A Guide to Taking Action and Generating Evidence is a 2006 report produced by WHO and the International Society for Prevention of Child Abuse and Neglect
- The US Centers for Disease Control and Prevention provides information on child maltreatment and links to additional resources
- The National Society for the Prevention of Cruelty to Children (NSPCC) is a not-for-profit organization that aims to end all cruelty to children in the UK; Childline is a resource provided by the NSPCC that provides help, information, and support to children who are being abused
- The Hideout is a UK-based website that helps children and young people understand domestic abuse
- Childhelp is a US not-for-profit organization dedicated to helping victims of child abuse and neglect; its website includes a selection of personal stories about child maltreatment

## Introduction

Child maltreatment is defined as all forms of physical and/or emotional ill-treatment, sexual abuse, neglect or negligent treatment, or commercial or other exploitation of children that results in actual or potential harm to a child's health, survival, development, or dignity in the context of a relationship of responsibility, trust, or power [1]. Four types of maltreatment are commonly recognised: sexual abuse, physical abuse, emotional abuse (also referred to as psychological abuse), and neglect (Table 1).

**Table 1:** Definition of child maltreatment.

Type of Maltreatment	Description
Physical abuse	Physical abuse of a child is defined as the intentional use of physical force against a child that results in—or has a high likelihood of resulting in—harm for the child's health, survival, development, or dignity. This includes hitting, beating, kicking, shaking, biting, strangling, scalding, burning, poisoning, and suffocating. Much physical violence against children in the home is inflicted with the object of punishing.
Sexual abuse	Sexual abuse is defined as the involvement of a child in sexual activity that he or she does not fully comprehend, is unable to give informed consent to, or for which the child is not developmentally prepared, or else that violates the laws or social taboos of society. Children can be sexually abused by both adults and other children who are—by virtue of their age or stage of development—in a position of responsibility, trust, or power over the victim.
Emotional and psychological abuse	Emotional and psychological abuse involves both isolated incidents, as well as a pattern of failure over time on the part of a parent or caregiver to provide a developmentally appropriate and supportive environment. Acts in this category may have a high probability of damaging the child's physical or mental health, or his/her physical, mental, spiritual, moral, or social development. Abuse of this type includes the following: the restriction of movement; patterns of belittling, blaming, threatening, frightening, discriminating against, or ridiculing; and other non-physical forms of rejection or hostile treatment.
Neglect	Neglect includes both isolated incidents, as well as a pattern of failure over time on the part of a parent or other family member to provide for the development and well-being of the child—where the parent is in a position to do so—in one or more of the following areas: health, education, emotional development, nutrition, shelter, and safe living conditions. The parents of neglected children are not necessarily poor.

Adapted from Butchart et al. [5].

There is a great deal of uncertainty around estimates of the frequency and severity of child maltreatment worldwide. Furthermore, much violence against children remains largely hidden and unreported because of fear and stigma and the societal acceptance of this type of violence [2]. Globally, prevalence of reported child sexual abuse varies from 2% to 62%, with some of this variation explained by a number of methodological factors including definition of abuse, method of data collection, and type of sample assessed [3]. In high-income countries, the annual prevalence of physical abuse ranges from 4% to 16%, and approximately 10% of children are neglected or emotionally abused [4]. Eighty percent of this maltreatment is perpetrated by parents or parental guardians [4], and poverty, mental health problems, low educational achievement, alcohol and drug misuse, having been maltreated oneself as a child, and family breakdown or violence between other family members are all important risk factors for parents abusing their children [5].

There is growing recognition that different forms of interpersonal violence have a large public health impact [6]. In children, the consequences of violence can vary widely. Physical injuries and, in extreme cases, death are direct consequences. World Health Organization (WHO) estimates of child homicide suggest that infants and very young children are at greatest risk, with rates for the 0- to 4-y age group about double those for 5- to 14-y-olds as a result of their dependency and vulnerability [5]. However, in the majority of non-fatal cases, the direct physical injury causes less morbidity to the child than the long-term impact of the violence on the child's neurological, cognitive, and emotional development and overall health [5].

Child maltreatment is a major public health problem, yet a lack of understanding of its serious lifelong consequences and of the cost and burden on society has hampered investment in prevention policies and programs. In order to effectively respond to the problem, the WHO 2006 report on prevention of child maltreatment [5] recommended expanding the scientific evidence base for the magnitude, consequences, and preventability of child maltreatment.

The relationship between child sexual abuse and adverse psychological consequences in adults is well established [7]–[9], and in the WHO comparative risk assessment study, Andrews and colleagues [3] carried out a systematic review and meta-analysis summarising the evidence of a relationship between child sexual abuse and subsequent mental disorders. This review is currently being updated in the new iteration of the Global Burden of Diseases, Injuries, and Risk Factors Study, aiming to provide global estimates of attributable burden for 1990 to 2010 [10], but other forms of child maltreatment have been omitted.

Exposure to non-sexual child maltreatment, namely, physical abuse, emotional abuse, and neglect, is associated with increased risk of a wide range of psychological and behavioural problems, including depression, alcohol abuse, anxiety, and suicidal behaviour, and with increased risk of HIV and herpes simplex virus type 2 (HSV2) infection [11]–[14]. However, the long-term health consequences of these other forms of child maltreatment have not been systematically examined. To address these omissions, clarify the present state of empirical research, and enable the quantification of the health impacts of child neglect, physical abuse, and emotional abuse at the population level using burden of disease and comparative risk assessment methodology, we conducted a systematic review of the scientific literature and quantitative meta-analyses. To the best of our knowledge, this is the first meta-analysis to summarise the evidence for associations between individual types of non-sexual child maltreatment and outcomes related to mental and physical health.

## Methods

General recommendations from the PRISMA 2009 revision [15], with regard to processing and reporting of results, were taken into account (Text S1). The meta-analysis conforms to the guidelines outlined by the Meta-analysis of Observational Studies in Epidemiology recommendations [16]. Methods and inclusion criteria were specified in advance and documented in a review protocol (Text S2).

### Inclusion and Exclusion Criteria

This systematic review and meta-analysis incorporated retrospective and prospective cohort, cross-sectional, and case-control studies meeting the following inclusion criteria: (1) the study reported original, empirical research published in a peer-reviewed journal, (2) the study considered non-sexual child maltreatment as a potential risk factor for loss of health, and (3) the related health outcomes or behavioural risk factors were among those listed in the Global Burden of Diseases, Injuries, and Risk Factors Study [10]. Studies reporting exposure only to combined types of abuse were excluded. Included studies reported odds ratios (ORs) and confidence intervals (CIs) comparing those exposed and not exposed by type of abuse or, alternatively, provided the information from which effect sizes and confidence intervals could be calculated (Text S2).

### Search Strategy

Three electronic databases (Medline, EMBASE, and PsycINFO up to 26 June 2012) were searched using full text and Medical Subject Headings (MeSH) terms to identify studies reporting an association between non-sexual child maltreatment and health outcomes (Text S2). Truncation of terms was used to capture variation in terminology. The search was not restricted to the English language, nor restricted by any other means. Searches were conducted using synonyms and combinations of the following search terms: “maltreatment”, “physical abuse”, “psychological abuse”, and “emotional abuse”, and automatic explosion of the terms “child abuse” and “child neglect”. The search was also not restricted to any particular health outcome. Instead, the broader terms “risk”, “adverse effect”, “consequences”, “harm”, and “association” were used to encompass all studies that investigated any adverse outcome of non-sexual child maltreatment. In addition, reference lists of selected studies were screened for any other relevant study, and additional studies were also identified through contact with study authors. Articles in languages other than English were translated.

### Data Collection and Quality Assessment

The full-text article of any study that appeared to meet the inclusion criteria was retrieved for closer examination. Two reviewers (R. E. N. and M. B.) independently assessed articles for eligibility. Disagreements were resolved by consensus. The coders were not masked to the journals or authors of the studies reviewed. A standardised data extraction sheet was developed, and data retrieved included publication details, country where study was conducted, methodological characteristics such as sample size and study design, exposure and outcome measures, type of abuse, and health outcomes (Text S2). The data extraction sheet included a quality assessment tool (Table 2) to rate the methodological quality of each study based on the Newcastle-Ottawa Scale for assessing the quality of observational studies [17]. Quality assessment was completed independently by two reviewers, and disagreements were resolved by discussion. One author was contacted for further information.

**Table 2:** Assessment of study quality.

Quality Criteria	Quality Score
**Representativeness of the population**	Population-based representative = 1
	Not representative, selected group, volunteers, or no description = 0
**Ascertainment of exposure to child abuse and neglect**	Data on child maltreatment collected prospectively = 1
	Data on child maltreatment collected retrospectively = 0
**Selection of the non-exposed cohort/controls**	Drawn from the same population = 1
	Drawn from a different source or no description = 0
**Assessment of child abuse and neglect**	Secure official record (court-substantiated abuse) = 1
	Self-reported or structured interview or self-administered questionnaire or no description = 0
**Case definition for child abuse and neglect**	Uses WHO definitions of child maltreatment or court-substantiated abuse or Barnett-Cicchetti Maltreatment Classification System = 1
	Marks and bruises (physical abuse), questions from scales (e.g., Childhood Trauma Questionnaire), published surveys, or own system = 0
**Assessment of outcome**	Use of structured clinical interview for DSM-III/IV (DIS, DISC, CIDI) (mental health); direct physical measurements or blood tests (physical health) = 1
	Questions from published health surveys/screening instruments, own system, symptoms described, no system, not specified, or self-reported = 0
**Adequacy of follow-up of cohorts (where relevant) or response rate**	Completeness good (≥80%), with description of those lost to follow-up = 1
	Completeness poor (<80%) or no statement = 0
**Appropriate statistical analysis**	Yes = 1
	No = 0
**Appropriate methods to control confounding**	Yes = 1 (multivariable adjusted OR including SES, education, or family dysfunction in models)
	No = 0 (univariate analysis or controls for age/sex only)
**Source of funding declared**	Yes (financial disclosure, funding/support/grant declared) = 1
	No = 0

CIDI, Composite International Diagnostic Interview; DIS, Diagnostic Interview Schedule; DISC, Diagnostic Interview Schedule for Children; SES, socioeconomic status.

### Statistical Analyses

Weighted summary measures were computed using MetaXL, version 1.2 [18], a tool for meta-analysis in Microsoft Excel, with ORs chosen as the principal summary measure. Heterogeneity was quantitatively assessed using the Cochran's *Q* and *I* ^2^ statistics to evaluate whether the pooled studies represent a homogeneous distribution of effect sizes. Evidence of publication bias was investigated by means of funnel plots using the standard error on the *y*-axis [19].

Meta-analyses were complicated by the presence of significant heterogeneity in the data, likely due to a combination of true variance in these relationships and variability produced by differences in the methodology used to measure exposure and outcomes. We hypothesised that effect size may differ according to the methodological quality of the studies. MetaXL implements a process to explicitly address study heterogeneity caused by differences in study quality. This so-called quality effects (Doi and Thalib) model [20] is a modified version of the fixed-effects inverse variance method that additionally allows giving greater weight to studies of high quality versus studies of lesser quality by using the quality scores assigned to each study to weigh studies not only according to sample size but also by study quality [20],[21]. Forest plots were made to visualise individual as well as pooled effects.

To address the effects of important study characteristics and explore heterogeneity, we additionally conducted several pre-specified subgroup analyses (depending on data availability) by the following: gender of participants in the sample, geographic location (high income versus low-to-middle income), type of sample (population-based versus non-representative samples), measurement of abuse (self-reported versus official records), assessment of health outcome (structured clinical interview versus self-reported), prospective versus retrospective assessment of abuse and neglect, and appropriate adjustment versus no or inadequate adjustment for confounders.

## Results

Out of 285 articles assessed for eligibility, 124 studies provided evidence of a relationship between non-sexual child maltreatment and various health outcomes for use in subsequent meta-analyses (Figure 1). The majority (*n* = 112) were from Western Europe, North America, Australia, and New Zealand. Data from low- and middle-income countries were sparse. Only 16 studies used a prospective cohort design that followed abused or neglected children over time to identify later health outcomes (Table 3). The remaining studies included cohort, cross-sectional, and case-control studies that measured the maltreatment retrospectively, usually by self-report in adolescence or adulthood. Most of the studies included in our meta-analysis presented data from regional or nationally representative samples (Table 3). The results of primary meta-analyses are presented in Tables 4–6, with Figures S1, S2, S3, S4, S5, S6, S7, S8, S9, S10, S11, S12, S13, S14, S15, S16, S17, S18, S19, S20, S21, S22, S23, S24, S25, S26, S27, S28, S29, S30, S31, S32, S33, S34, S35, S36, S37, S38, S39, S40, S41, S42 showing the forest plots of these meta-analyses. Details of subgroup analyses are presented in Tables S1, S2, S3, S4, S5, S6, S7, S8, S9, S10, S11.

**Figure 1:**
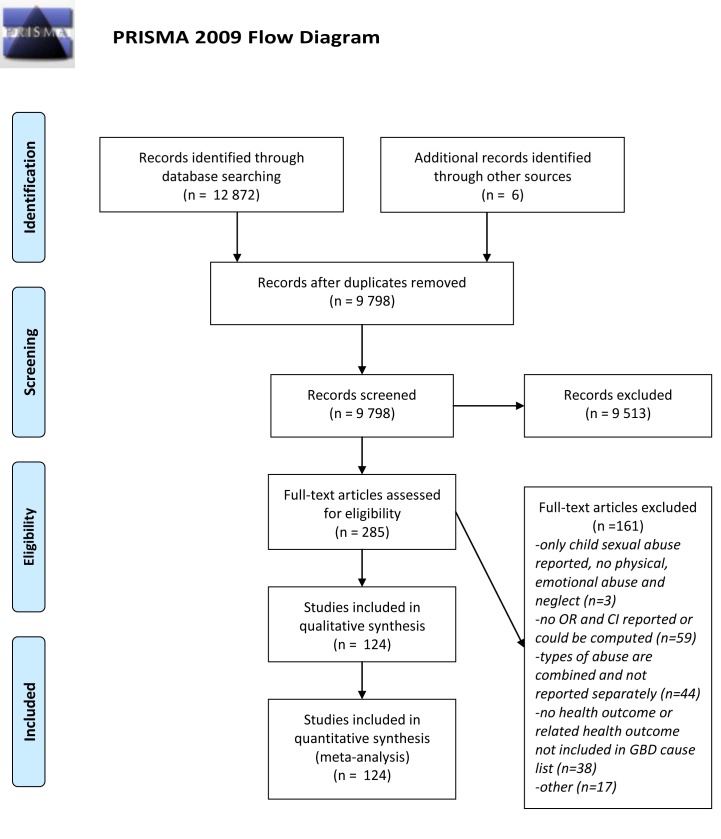
PRISMA flow diagram showing process of study selection for inclusion in systematic review and meta-analyses.

**Table 3:** Summary of meta-analysis study characteristics.

First Author [Reference]	Year	Setting	Sample Size (*N*)	Percent Female	Type of Maltreatment	Child Maltreatment Measurement	Assessment of Health Outcome	Health Outcomes	Ascertainment of Exposure to Child Maltreatment/Study Type	Sample
Afifi [54]	2006	US	5,838	50.5%	Physical punishment	Face-to-face interviews using CTS	CIDI	Major depression, anxiety, alcohol problems	Retrospective/cross-sectional	Population-based
Afifi [26]	2008	US	5,692	Not given	Physical abuse	Face-to-face interviews	CIDI	Anxiety, substance abuse, self-inflicted injuries	Retrospective/cross-sectional	Population-based
Afifi [55]	2012	US	34,653	40.6% for physical punishment and 52.3% for no punishment	Harsh physical punishment (excludes abuse)	Face-to-face interviews, items adapted from ACE questionnaire	AUDADIS-IV	Major depression, dysthymia, anxiety disorders, alcohol, drug use	Retrospective/cross-sectional	Population-based
Anda [94]	1999	US	9,215	53.80%	Physical and emotional abuse	Self-administered ACE questionnaire^a^	Self-reported	Current smoking, early smoking initiation	Retrospective/cohort	HMO members
Anda [82]	2010	US	17,337	54%	Physical and emotional abuse	Self-administered ACE questionnaire^a^	Self-reported	Frequent headaches	Retrospective/cohort	HMO members
Astin [95]	1995	US	87	100%	Physical abuse	SCID for DSM-III-R	SCID for DSM-III-R	PTSD	Retrospective/cross-sectional	Battered women
Bennett [96]	1994	US	733	100%	Physical abuse	Self-administered questionnaire—own questions	Self-administered questionnaire—own questions	Substance abuse	Retrospective/cross-sectional	Convenience sample of mothers
Bensley [97]	2000	US	3,473	50.7%	Physical abuse	Telephone survey—own questions	Self-reported	HIV risk behaviours, heavy drinking	Retrospective/cross-sectional	Population-based
Bentley [98]	2009	US	713	53.4%	Physical abuse and neglect	Official record	Height and weight measurements, BMI>30 kg/m^2^	Obesity	Prospective/cohort	Abused youth
Bonomi [99]	2008	US	3,568	100%	Physical abuse	Telephone interview	Self-reported (CES-D for depression/presence-of-symptom surveys)	Depressive disorders, back pain, headache/migraine, diarrhoea	Retrospective/cross-sectional	Insured women
Boynton-Jarrett [100]	2011	US	68,505	100%	Physical abuse	Self-administered questionnaire with items from CTQ and CTS	Hysterectomy/ultrasound confirmation	Uterine leiomyoma	Retrospective/cohort	Pre-menopausal nurses
Bremner [101]	1993	US	66	0%	Physical abuse	Self-reported, using CSTE	SCID for DSM-III-R	PTSD	Retrospective/case-control	Viet Nam combat veterans
Brezo [27]	2008	Canada	1,684	47.2%	Physical abuse	Interview using CTS	DIS-III-R, DISC-II, SSI	Suicide ideation/attempt	Retrospective/cohort	Population-based
Brown [102]	1999	US	639	47.7%	Physical abuse and neglect	Combined official records and self-reported abuse and neglect	DISC-I	Major depression, dysthymia, depressive disorders, self-inflicted injuries	Retrospective/cohort	Population-based
Chapman [40]	2004	US	9,460	54%	Physical and emotional abuse	Self-administered ACE questionnaire^a^	Some questions from CES-D	Depressive disorders	Retrospective/cohort	HMO members
Chartier [103]	2009	Canada	8,116	50.2%	Physical abuse	Self-administered questionnaire	CIDI structured face-to-face interview (alcohol abuse) and self-administered questionnaire	Smoking, alcohol abuse, low exercise, obesity, risky sexual behaviour	Retrospective/cross-sectional	Population-based
Cohen [104]	2001	US	664	50.3%	Physical abuse and neglect	Official records of abuse and neglect and self-reported abuse and neglect	DISC-I and symptom scales	Depressive disorders, anxiety, childhood behavioural disorders, substance abuse	Retrospective/cohort	Population-based
Coid [105]	2003	UK	1,207	100%	Beaten by parent	Self-administered questionnaire	Self-reported symptom scale (anxiety/depression), CAGE (alcohol problems)	Anxiety, depression, PTSD, suicide attempt, self-inflicted injuries, drug use, alcohol problems	Retrospective/cross-sectional	Primary care patients
Conroy [106]	2009	Australia	1,313	43.5%	Physical and emotional abuse, and neglect	Structured face-to-face interview	History of opioid pharmacotherapy	Opioid dependence	Retrospective/case-control	Not representative
Cougle [73]	2010	US	4,141	56%	Physical abuse	Structured face-to-face interview	CIDI	Anxiety disorders	Retrospective/cross-sectional	Population-based
Courtney [107]	2008	US	92	81.5%	Emotional abuse	Self-administered questionnaire using CTQ	BDI-II	Depressive symptoms	Retrospective/cohort	Adolescent primary care patients
Dong [108]	2004	US	17,337	54%	Physical and emotional abuse, and neglect	Self-administered ACE questionnaire^a^	Self-reported	Ischaemic heart disease	Retrospective/cohort	HMO members
Draper [109]	2008	Australia	22,251	58.7%	Physical abuse	Self-administered questionnaire—own questions	Self-reported	Current smoking, alcohol problems, diabetes, cardiovascular disease, COPD, cancer	Retrospective/cross-sectional	Population-based
Dube [110]	2001	US	17,337	54%	Physical and emotional abuse	Self-administered ACE questionnaire^a^	Self-reported	Self-inflicted injuries	Retrospective/cohort	HMO members
Dube [111]	2003	US	8,613	54%	Physical and emotional abuse, and neglect	Self-administered ACE questionnaire^a^	Self-reported	Drug use	Retrospective/cohort	HMO members
Dube [112]	2006	US	8,417	54%	Physical and emotional abuse, and neglect	Self-administered ACE questionnaire^a^	Self-reported	Ever use of alcohol, early alcohol initiation (≤14 y)	Retrospective/cohort	HMO members
Duke [28]	2010	US	136,549	50.2%	Physical abuse	Self-reported based on ACE questionnaire	Self-reported	Suicide ideation/attempt, self-harm	Retrospective/cross-sectional	Population-based
Duncan [57]	1996	US	4,008	100%	Physical assault	Telephone interview ICI	SCID for DSM-III-R	Major depressive episode, PTSD, drug use	Retrospective/cross-sectional	Population-based
Egeland [113]	2002	US	140	Not given	Physical abuse and emotional neglect	Official records (physical abuse); project staff assessment (neglect)	K-SADS	Conduct disorders	Prospective/cohort	High-risk youth
Enns [114]	2006	Netherlands	7,076	Not given	Physical and emotional abuse, and neglect	Face-to-face interviews—standardised questions	CIDI	Self-inflicted injuries	Retrospective/cohort	Population-based
Evans-Campbell [115]	2006	US	112	100%	Physical abuse	Face-to-face interviews—own questions	Self-reported	HIV risk behaviour	Retrospective/cross-sectional	Representative sample of American Indian/Alaska Native
Fergusson [41]	2008	New Zealand	1,265	Not given	Physical abuse/punishment	Face-to-face interviews—own questions	CIDI	Major depression, mental disorders, substance abuse, self-inflicted injuries	Retrospective/cohort	Population-based
Fergusson [116]	2008	New Zealand	1,265	Not given	Physical abuse/punishment	Face-to-face interviews—own questions	CIDI	Illicit drug use/dependence	Retrospective/cohort	Population-based
Flisher [117]	1996	South Africa	7,340	54%	Physical abuse/injury	Self-administered questionnaire—own questions	Self-reported	Suicide attempt	Retrospective/cross-sectional	Students
Fuemmeler [74]	2009	US	15,197	Not given	Physical abuse and neglect	Self-reported	Height and weight measurements, BMI>30 kg/m^2^	Obesity	Retrospective/cohort	Population-based
Fujiwara [118]	2011	Japan	1,722	49.4%	Physical abuse and neglect	Modified version of CTS	CIDI	Anxiety disorders, intermittent explosive disorder, substance abuse	Retrospective/cross-sectional	Population-based
Fuller-Thomson [62]	2009	Canada	13,092	51.6%	Physical abuse	Self-reported	Self-reported	Cancer	Retrospective/cross-sectional	Population-based
Fuller-Thomson [119]	2009	Canada	11,108	51.4%	Physical abuse	Self-reported	Self-reported	Osteoarthritis	Retrospective/cross-sectional	Population-based
Fuller-Thomson [63]	2010	Canada	13,093	51.6%	Physical abuse	Self-reported	Self-reported	Heart disease	Retrospective/cross-sectional	Population-based
Fuller-Thomson [61]	2010	Canada	13,089	56.1%	Physical abuse	Self-reported	Self-reported	Migraine	Retrospective/cross-sectional	Population-based
Fuller-Thomson [120]	2011	Canada	13,069	56.1%	Physical abuse	Self-reported	Self-reported	Peptic ulcer	Retrospective/cross-sectional	Population-based
Gal [121]	2011	Israel	4,859	50.8%	Physical abuse	Face-to-face interviews	CIDI	Anxiety disorders	Retrospective/cross-sectional	Population-based
Goodwin [122]	2002	US	3,032	Not given	Physical and emotional abuse	Self-administered questionnaire using CTS	Self-reported	Type 2 diabetes	Retrospective/cross-sectional	Population-based
Goodwin [65]	2003	US	3,032	Not given	Physical abuse	Self-administered questionnaire using CTS	CIDI for mental disorders and self-reported for physical	Migraine headache, ulcers	Retrospective/cross-sectional	Population-based
Goodwin [68]	2003	US	5,877	Not given	Physical abuse	Self-administered questionnaire using CTS	CIDI for mental disorders and self-reported for physical	Major depression, alcohol dependence, hypertension	Retrospective/cross-sectional	Population-based
Goodwin [66]	2004	US	5,877	Not given	Physical abuse and neglect	Self-administered questionnaire—own questions	CIDI for mental disorders and self-reported for physical	Self-reported arthritis, hypertension, ulcer, neurological disorders, diabetes	Retrospective/cross-sectional	Population-based
Goodwin [64]	2005	NZ	983	Not given	Physical abuse/punishment	Face-to-face interviews—own questions	CIDI	Panic disorders	Retrospective/cohort	Population-based
Goodwin [67]	2012	US	3,032	Not given	Physical abuse	Self-administered questionnaire	Self-reported	Respiratory disease	Retrospective/cross-sectional	Population-based
Gould [123]	1994	US	292	71%	Physical and emotional abuse	Self-administered questionnaire	Self-reported	Suicide attempt	Retrospective/cross-sectional	Convenience sample, primary care
Green [124]	2010	US	5,692	42%	Physical abuse and neglect	Face-to-face interviews with modified form of the CTS	CIDI	Anxiety, substance use, disruptive behaviour	Retrospective/cross-sectional	Population-based
Griffin [75]	2010	US	290	100%	Physical abuse	Face-to-face interviews	Self-reported	Alcohol problem	Retrospective/cross-sectional	Non-probability sample
Gunstad [125]	2006	Australia, US, UK, and the Netherlands	696	51.30%	Emotional abuse	Self-administered modified Child Abuse and Trauma Scale	Self-reported height and weight	BMI, obesity	Retrospective/cross-sectional	Not representative
Hamburger [126]	2008	US	3,559	52%	Physical abuse	Self-administered questionnaire	Self-reported	Alcohol use/problems	Retrospective/cross-sectional	Students in high-risk community
Hanson [127]	2001	US	4,008	100%	Physical abuse (aggravated assault)	Face-to-face interviews—own questions	SCID for DSM-III-R	Major depressive episode, PTSD	Retrospective/cross-sectional	Population-based
Haydon [76]	2011	US	8,922	55.5%	Physical abuse and neglect	Computer-assisted self-interview	Test-identified current STD	Current STDs	Retrospective/cohort	Population-based
Hillis [128]	2000	US	9,323	54.30%	Physical and emotional abuse	Self-administered ACE questionnaire^a^	Self-reported	STDs	Retrospective/cohort	HMO members
Hovens [22]	2010	Netherlands	1,931	Not given	Physical abuse, emotional abuse, emotional neglect	Face-to-face interviews	CIDI	Current depressive disorders, anxiety disorders	Retrospective/cross-sectional	Population-based
Huang [129]	2011	US	4,882	49.3%	Physical abuse and neglect	Interview using items consistent with CTS and CTQ	Self-reported	Drug use	Retrospective/cohort	Population-based
Jeon [130]	2009	South Korea	6,986	37.5%	Physical and emotional abuse	Self-administered questionnaire ETISR-SF	Self-reported	Suicide ideation/attempt	Retrospective/cross-sectional	Medical students
Jewkes [13]	2010	South Africa	2,782 (1,367 men and 1,415 women)	50.9%	Physical punishment, emotional abuse, emotional neglect	Face-to-face interviews with modified form of the CTQ	Self-reported using CES-D, blood test for HIV and HSV2	HIV and HSV2 infection, depressive disorders, alcohol/drug abuse, self-inflicted injuries	Retrospective/cross-sectional for psycho-social outcome measures, longitudinal analysis for risk of HIV and HSV2 infection	Volunteer sample
Jirapramukpitak [77]	2005	Thailand	202	58%	Physical and emotional abuse	Self-administered questionnaire using CTS	Lay-administered CIS-R for mental disorders, AUDIT for alcohol	Drug use, alcohol problems	Retrospective/cross-sectional	Population-based
Johnson [23]	2002	US	782	49%	Physical neglect, harsh maternal punishment	Maternal behaviour assessed by interviewer	DISC-I	Eating disorders, obesity	Prospective/cohort	Population-based
Juang [131]	2004	Taiwan	116	67%	Neglect	Neglect assessed by teacher interviews (GFES)	By neurologist using S-L criteria	Chronic daily headache	Case-control	Convenience sample of students
Jun [132]	2008	US	68,505	100%	Physical abuse	Self-administered questionnaire with items from CTQ	Self-reported	Adolescent smoking	Retrospective/cohort	Nurses
Kaplan [133]	1998	US	99 abused and 99 non-abused adolescents	50%	Physical abuse	Official records	SCID for DSM-III-R	Depressive disorder, childhood behavioural disorders, drug use, cigarette use	Retrospective/cross-sectional	Abused youth
Kerr [134]	2009	Canada	560	34%	Physical abuse	Interviewer-administered questionnaire using CTQ	Self-reported	Injection drug use	Retrospective/cohort	Street youth
Lau [135]	2003	China	489	38.2%	Physical abuse and punishment	Face-to-face interview—own questionnaire	Achenbach Child Behavior Checklist	Substance use, smoking, self-inflicted injuries	Retrospective/cross-sectional	Population-based
Levitan [136]	2003	Canada	6,597	61%	Physical abuse	Self-administered questionnaire—own questions	CIDI	Depressive disorders, anxiety, comorbid depressed and anxious	Retrospective/cross-sectional	Population-based
Libby [69]	2004	US	3,084 (1,446 from southwest area and 1,638 from northern plains area)	57.3% in southwest; 51.75% in northern plains	Physical abuse	Face-to-face interviews—own questions	CIDI	Alcohol use/dependence, drug use/dependence	Retrospective/cross-sectional	Population-based
Libby [137]	2005	US	3,084 (1,446 from southwest area and 1,638 from northern plains area)	57.3% in southwest; 51.75% in northern plains	Physical abuse	Face-to-face interviews—own questions	CIDI	Depressive disorders, anxiety, PTSD	Retrospective/cross-sectional	Population-based
Lissau [138]	1994	Denmark	756	Not given	Neglect	School medical service answered a questionnaire about the hygiene of the child	Height and weight measurements	Obesity	Prospective/cohort	Population-based
Logan [139]	2009	US	1,484	Not given	Physical abuse	Self-administered questionnaire	Self-reported	Suicide ideation/attempt, drug use	Retrospective/cross-sectional	High-risk youth
Macmillan [70]	2001	Canada	7,016	52.4%	Physical abuse	Self-administered questionnaire using CTS	CIDI	Major depression, anxiety, alcohol abuse/dependence, drug abuse/dependence	Retrospective/cross-sectional	Population-based
Mullen [29]	1996	New Zealand	497	100%	Emotional abuse	Face-to-face interviews—PBI	PSE	Eating disorder, suicide attempt, depression	Retrospective/cross-sectional	Population-based
Nichols [71]	2004	US	722	100%	Physical abuse	Self-administered questionnaire—own questions derived from CTS	Self-reported	Smoking	Retrospective/cohort	Population-based
Nikulina [140]	2011	US	1,005	47.3%	Neglect	Official record	Diagnostic interview-DIS-III-R	PTSD, major depression	Prospective/cohort	Abused youth
Perkins [141]	2002	US	100,236	100%	Physical abuse	Self-administered questionnaire—own questions	ABQ	Bulimia (purging two or more times per week)	Retrospective/cross-sectional	Students, not representative
Pillai [142]	2009	India	3,662	51.4%	Physical abuse	Face-to-face interviews	Self-reported	Suicide ideation/attempt	Retrospective/cross-sectional	Population-based
Ramiro [143]	2010	Philippines	1,068	50.1%	Physical and emotional abuse, and neglect	Self-administered ACE questionnaire^a^	Self-reported	Current smoking, alcohol, drug use, risky sexual behaviour, suicide attempt	Retrospective/cross-sectional	Population-based
Rich-Edwards [78]	2010	US	67,853	100%	Physical abuse	Self-administered questionnaire with items from CTQ	Self-reported	Type 2 diabetes	Retrospective/cohort	Nurses
Riley [144]	2010	US	68,505	100%	Physical abuse	Self-administered questionnaire with items from CTQ	Self-reported	Hypertension	Retrospective/cohort	Nurses
Ritchie [145]	2009	France	942	58.1%	Physical punishment and emotional abuse	Self-reported	MINI, CES-D, anti-depressant treatment	Depressive disorders	Retrospective/cross-sectional	Elderly (65+ y)
Roberts [32]	2008	US	11,394	Not given	Physical abuse	Self-administered questionnaire—own questions	Self-reported smoking, CES-D for depression	Ever regular smoking	Retrospective/cross-sectional	Population-based
Rohde [146]	2008	US	4,641	100%	Physical abuse	Telephone interview based on CTQ	Self-reported height and weight, depression	Obesity, depression	Retrospective/cross-sectional	Health plan members
Romans [147]	2002	New Zealand	477	100%	Physical abuse	Face-to-face interview—own questions	Self-reported	Headache/migraine, asthma, diabetes, CVD	Retrospective/cross-sectional	Population-based
Rubino [148]	2009	Italy	788	56.5% for controls	Physical and emotional abuse	Self-reported	SCID for DSM-IV	Schizophrenia, depression	Retrospective/case-control	Voluntary inpatients
Schneider [79]	2007	US	3,936	100%	Physical and emotional abuse	Self-administered questionnaire—TSS for physical abuse and CTS for emotional abuse	CDC Healthy Days Measure, PC-PTSD	Anxiety, PTSD	Retrospective/cross-sectional	Population-based
Schoemaker [42]	2002	Netherlands	1,987	100%	Physical and emotional abuse, and neglect	Face-to-face interviews—own questions	CIDI	Bulimia nervosa	Retrospective/cohort (uses cross-sectional data)	Population-based
Scott [149]	2008	Americas, Europe, Japan	18,303	52.7%	Physical abuse and neglect	Face-to-face interviews	Self-reported	Asthma	Retrospective/cross-sectional	Population-based
Scott [150]	2011	Americas, Europe, Japan	18,303	52.7%	Physical abuse and neglect	Face-to-face interviews	Self-reported	Heart disease, diabetes, chronic spinal pain, headache	Retrospective/cross-sectional	Population-based
Sidhartha [151]	2006	India	1,205	40%	Physical abuse and neglect	Self-administered questionnaire—AISS	Self-reported	Suicidal behaviour	Retrospective/cross-sectional	School students
Silverman [30]	1996	US	375	50%	Physical abuse	Face-to-face interviews—own questions	YSR and CDI (age 15 y), DIS-III-R (age 21 y)	Major depression, PTSD, alcohol abuse/dependence, drug abuse/dependence, self-inflicted injuries	Retrospective/cohort	Population-based
Smith [152]	2005	US	884	27.10%	Physical abuse and neglect (adolescent)	Official records (using Barnett-Cicchetti Maltreatment Classification System)	Self-reported	Drug use	Prospective/cohort	High-risk youth
Springer [153]	2007	US	2,051	55.6%	Physical abuse	Self-administered questionnaire based on CTS	Self-reported using CES-D (mental health), self-reported (physical)	Depressive disorders, asthma, high blood pressure, allergies	Retrospective/cohort	Population-based
Springer [154]	2009	US	3,317	52%	Physical abuse	Self-administered questionnaire based on CTS	Self-reported	Bronchitis/emphysema, ulcers	Retrospective/cohort	Population-based
Stein [155]	1996	Canada	122 cases 124 controls	42.4% for controls	Physical abuse	Semistructured interview	SCID for DSM-IV	Anxiety disorders	Retrospective/case-control	Population-based
Stein [156]	2010	Americas, Europe, Japan	18,630	52.8%	Physical abuse and neglect	Face-to-face interviews	Self-reported	Hypertension	Retrospective/cross-sectional	Population-based
Straus [56]	1994	US	2,149	Not given	Physical punishment (adolescent)	Face-to-face interviews—CTS	Four items from PERI Life Events Scale	Depressive symptoms, self-inflicted injuries, alcohol abuse	Retrospective/cross-sectional	Population-based
Strine [72]	2012	US	7,279	54%	Physical and emotional abuse, and neglect	Self-administered ACE questionnaire^a^	Self-reported	Alcohol problems	Retrospective/cohort	HMO members
Thomas [157]	2008	UK	9,310	Not given	Physical and emotional abuse, and neglect	self-administered questionnaire based on ACE questionnaire^a^ (retrospective); local authority health visitor interviewed parents at child ages 7, 11, and 16 y (prospective)	Measured weight, height, and waist circumference, blood glucose levels	Obesity, type 2 diabetes	Prospective and retrospective/cohort	Population-based
Thompson [158]	2002	US	8,000	100%	Physical victimisation	Telephone interview—CTS	Self-reported	Drug use, alcohol use	Retrospective/cross-sectional	Population-based
Thompson [159]	2004	US	16,000	50%	Physical abuse	Telephone interview—CTS	Self-reported	Drug use, alcohol use	Retrospective/cross-sectional	Population-based
Thompson [160]	2012	US	740	52.6%	Physical and emotional abuse, and neglect	Official records (neglect); self-reported (physical/emotional)	Self-reported	Suicide ideation	Retrospective/cohort	High-risk youth
Timko [161]	2008	US	6,942	100%	Emotional abuse	Self-reported	Self-reported	Binge drinking	Retrospective/cross-sectional	Population-based
Trent [162]	2007	US	5,697	46.6%	Physical abuse	Self-administered questionnaire using CTS	MAST	Alcohol use, binge drinking	Retrospective/cross-sectional	Military personnel, not representative
Turner [163]	2003	Australia	9,512	100%	Physical and emotional abuse	Self-administered questionnaire—own questions	Self-reported	Illicit drug use	Retrospective/cohort	Population-based
Vander Weg [164]	2011	US	10,277	51.3%	Physical assault and emotional abuse	Telephone survey	Self-reported	Lifetime, current smoking	Retrospective/cross-sectional	Arkansas and Louisiana residents
Von Korff [165]	2009	Americas, Europe, Japan	18,309	52.5%	Physical abuse and neglect	Face-to-face interviews	Self-reported	Arthritis	Retrospective/cross-sectional	Population-based
Wainwright [166]	2002	UK	3,491	55.2%	Physical abuse	Self-administered questionnaire	Structured self-assessment	Major depression	Retrospective/cohort	Population-based
Wan [167]	2010	Hong Kong	2,754	44.3%	Physical abuse	Self-administered questionnaire adapted from CTQ	Self-reported+YSR	Suicide ideation/attempt	Retrospective/cross-sectional	Population-based
Welch [24]	1996	UK	306	100%	Physical abuse	investigator-based interview using own questionnaire	EDE diagnostic interview	Bulimia nervosa	Retrospective/case-control	Population-based
Widom [168]	1995	US	1,068	49%	Physical abuse and neglect	Official record	Diagnostic interview—DIS-III-R	Alcoholism	Prospective/cohort	Abused youth
Widom [169]	1996	US	1,187	49%	Physical abuse and neglect	Official record	Self-report interview	Risky sexual behaviour	Prospective/cohort	Abused youth
Widom [170]	1999	US	1,196	48.7%	Physical abuse and neglect	Official record and self-reported using own questionnaire based on CTS	Diagnostic interview—DIS-III-R	Drug abuse/dependence	Prospective and retrospective/cohort	Abused youth
Widom [171]	1999	US	1,196	49%	Physical abuse and neglect	Official record	Diagnostic interview—DIS-III-R	PTSD	Prospective/cohort	Abused youth
Widom [43]	2007	US	1,196	48.7%	Physical abuse and neglect	Official record	Diagnostic interview—DIS-III-R	Major depression	Prospective/cohort	Abused youth
Widom [33]	2012	US	754	52.9%	Physical abuse and neglect	Official record	Mantoux test, blood tests, blood pressure measurements, height and weight measurements, eye and hearing (Weber and Rinne) tests, oral examination	Tuberculosis, anaemia, malnutrition, hepatitis C, HIV, syphilis, hearing problems, vision loss, hypertension	Prospective/cohort	Abused youth
Williamson [31]	2002	US	13,177	51%	Physical and emotional abuse	Self-administered ACE questionnaire^a^	Height and weight measurements	Obesity (BMI≥30 kg/m^2^)	Retrospective/cohort	HMO members
Wilson [172]	2008	US	630	55.2%	Physical abuse and neglect	Official record	Diagnostic interview—DIS-III-R, blood tests	HIV-positive status, risky sexual behaviours	Prospective/cohort	Abused youth
Wilson [173]	2009	US	754	52.9%	Physical abuse and neglect	Official record	Self-reported	STDs	Prospective/cohort	Abused youth
Wilson [174]	2011	US	800	52.9%	Physical abuse and neglect	Official record	Self-reported	Risky sexual behaviour	Prospective/cohort	Abused youth
Wise [175]	2011	US	35,728	100%	Physical abuse	Mail questionnaire adapted from CTS	Self-reported	Breast cancer	Retrospective/cohort	Convenience sample of African-American women
Yates [25]	2008	US	164	49%	Physical abuse and physical neglect	Official records (physical abuse); project staff assessment (neglect)	SIBQ	Self-inflicted injury	Prospective/cohort	High-risk youth
Young [176]	2006	US	41,482	0%	Physical and emotional abuse, and neglect	Self-administered questionnaire—own questions based on ACE, CTS, and CTQ	AUDIT-C questionnaire	Risky drinking	Retrospective/cross-sectional	Military personnel

a Some ACE questionnaire categories were defined using items adapted from other questionnaires. These were the Conflict Tactics Scale (physical abuse, witnessing interparental violence, and emotional abuse) and the Childhood Trauma Questionnaire (emotional and physical neglect).

ABQ, Search Institute's Profiles of Student Life: Attitude and Behavior Questionnaire [177]; AISS, Adjustment Inventory for School Students [178]; AUDADIS-IV, Alcohol Use Disorders and Associated Disabilities Interview Schedule IV [179]; AUDIT, Alcohol Use Disorders Identification Test [180]; AUDIT-C, Alcohol Use Disorders Identification Test–alcohol consumption questions [181]; BDI-II, Beck Depression Inventory II [182]; CAGE, CAGE questionnaire [183]; CDC Healthy Days Measure, Centers for Disease Control and Prevention's Healthy Days Measure [184]; CDI, Children's Depression Inventory [185]; CES-D, Center for Epidemiologic Studies Depression Scale [186]; CIDI, Composite International Diagnostic Interview (a standardised diagnostic instrument) [187]; CIS-R, Clinical Interview Schedule–Revised [188]; COPD, chronic obstructive pulmonary disease; CSTE, Checklist of Stressful and Traumatic Events [189]; CTQ, Childhood Trauma Questionnaire [190]; CTS, Conflict Tactics Scale [191]; CVD, cardiovascular disease; DISC-I, National Institute of Mental Health Diagnostic Interview Schedule for Children I [192]; DISC-II, National Institute of Mental Health Diagnostic Interview Schedule for Children II [193]; DIS-III-R, National Institute of Mental Health Diagnostic Interview Schedule IIIR [194]; EDE, Eating Disorder Examination (a standardised investigator-based interview that operationalizes DSM-III-R criteria) [195]; ETISR-SF, Early Trauma Inventory Self Report–Short Form [196]; GFES, Global Family Environment Scale [197]; HMO, health maintenance organization; ICI, Incident Classification Interview [198]; K-SADS, Kiddie Schedule for Affective Disorders and Schizophrenia for School-Age Children [199]; MAST, Michigan Alcoholism Screening Test [200]; MINI, Mini International Neuropsychiatric Interview [201]; PBI, Parental Bonding Instrument [202]; PC-PTSD, Primary Care PTSD Screen [203]; PERI Life Events Scale, Psychiatric Epidemiological Research Instrument Life Events Scale [204],[205]; PSE, Present State Examination [206]; SSI, Scale for Suicide Ideation [207]; SIBQ, Self-Injurious Behavior Questionnaire [208]; S-L criteria, Silberstein-Lipton criteria [209]; SCID for DSM-III-R, Structured Clinical Interview for DSM-III-R [210]; SCID for DSM-IV, Structured Clinical Interview for DSM-IV [211]; TSS, Traumatic Stress Schedule [212]; YSR, Youth Self-Report [213].

**Table 4:** Summary of primary meta-analyses on mental health consequences of child non-sexual maltreatment.

Category	Health Outcome and Type of Maltreatment	Number of Data Points	Pooled OR	95% CI Lower Bound	95% CI Upper Bound	Cochran's *Q*	*I* ^2^ (%)	Test for Heterogeneity (*p-*Value)
**Mental disorders**	**Depressive disorders**							
	Physical abuse	36	1.54	1.16	2.04	273.81	87.22	<0.01
	Emotional abuse	9	3.06	2.43	3.85	21.99	63.63	<0.01
	Neglect	14	2.11	1.61	2.77	45.33	71.32	<0.01
	**Anxiety disorders**							
	Physical abuse	59	1.51	1.27	1.79	592.99	90.22	<0.01
	Emotional abuse	4	3.21	2.05	5.03	43.17	93.05	<0.01
	Neglect	8	1.82	1.51	2.20	11.24	37.74	0.13
	**Eating disorders**							
	Physical abuse	6	2.58	1.17	5.70	43.66	88.55	<0.01
	Emotional abuse	2	2.56	1.41	4.65	4.40	77.27	0.04
	Neglect	2	2.99	1.53	5.83	2.14	53.33	0.14
	**Childhood behavioural/conduct disorders**							
	Physical abuse	12	2.29	1.76	2.97	15.83	30.53	0.15
	Neglect	6	2.01	1.42	2.84	2.02	0.00	0.85
**Substance abuse/alcohol and drug use**	**Substance abuse**							
	Physical abuse	9	1.61	1.21	2.16	12.18	26.11	0.14
	Emotional abuse	1	2.00	0.60	6.30	Not pooled	Not pooled	Not pooled
	Neglect	2	1.29	0.67	2.47	2.39	58.20	0.12
	**Alcohol use**							
	Physical abuse: any alcohol use	44	1.30	1.10	1.55	207.27	79.25	<0.01
	Physical abuse: non-problem drinking	11	1.47	1.17	1.85	32.87	69.57	<0.01
	Physical abuse: problem drinking	33	1.26	1.03	1.55	153.20	79.11	<0.01
	Emotional abuse: any alcohol use	10	1.27	1.11	1.46	13.26	32.12	0.15
	Emotional abuse: non-problem drinking	2	1.29	0.88	1.90	4.28	76.62	0.04
	Emotional abuse: problem drinking	8	1.27	1.11	1.46	8.58	18.38	0.28
	Neglect: any alcohol use	15	1.14	0.92	1.39	100.32	86.04	<0.01
	Neglect: non-problem drinking	4	1.50	1.15	1.96	15.14	80.18	<0.01
	Neglect: problem drinking	11	1.09	0.87	1.35	50.38	80.15	<0.01
	**Drug use**							
	Physical abuse	43	1.92	1.67	2.20	136.06	69.13	<0.01
	Emotional abuse	8	1.41	1.11	1.79	30.51	77.06	<0.01
	Neglect	41	1.36	1.21	1.54	180.81	77.88	<0.01
**Suicidal behaviour**	Physical abuse	58	3.00	2.07	4.33	2,392.41	97.62	<0.01
	Emotional abuse	11	3.08	2.42	3.93	32.36	69.10	<0.01
	Neglect	15	1.85	1.25	2.73	19.43	27.94	0.15

**Table 5:** Summary of meta-analyses on sexually transmitted infections and risky sexual behaviour as consequences of child non-sexual maltreatment.

Health Outcome and Type of Maltreatment	Number of Data Points	Pooled OR	95% CI Lower Bound	95% CI Upper Bound	Cochran's *Q*	*I* ^2^ (%)	Test for Heterogeneity (*p-*Value)
**STIs/risky sexual behaviour**							
Physical abuse	33	1.78	1.50	2.10	49.12	34.85	0.03
Emotional abuse	5	1.75	1.49	2.04	2.96	0.00	0.57
Neglect	30	1.57	1.39	1.78	50.14	42.16	0.01
**HIV infection**							
Physical abuse	4	2.51	1.16	5.42	1.09	0.00	0.78
Emotional abuse	2	1.82	1.34	2.47	0.21	0.00	0.65
Neglect	2	2.50	0.77	8.15	0.29	0.00	0.59
**Other STIs**							
Physical abuse	12	1.53	1.13	2.07	17.27	7.65	0.10
Emotional abuse	2	1.56	1.26	1.93	0.76	0.00	0.38
Neglect	14	1.26	1.08	1.46	7.96	0.00	0.85
**Risky sexual behaviour**							
Physical abuse	17	1.95	1.58	2.40	23.37	31.54	0.10
Emotional abuse	1	2.10	1.50	3.00	Not pooled	Not pooled	Not pooled
Neglect	14	1.80	1.52	2.13	27.74	53.14	0.01

**Table 6:** Summary of primary meta-analyses on chronic diseases, lifestyle risk factors, and other physical health outcomes associated with exposure to child non-sexual maltreatment.

Category	Health Outcome and Type of Maltreatment	Number of Data Points	Pooled OR	95% CI Lower Bound	95% CI Upper Bound	Cochran's *Q*	*I* ^2^ (%)	Test for Heterogeneity (*p-*Value)
**Chronic diseases**	**Cardiovascular diseases**							
	***Stroke***							
	Physical abuse	3	1.76	0.56	5.51	0.78	0.00	0.68
	Neglect	2	3.00	0.99	9.10	0.57	0.00	0.45
	***Ischaemic heart disease***							
	Physical abuse	1	1.50	1.40	1.90	Not pooled	Not pooled	Not pooled
	Emotional abuse	1	1.70	1.50	1.90	Not pooled	Not pooled	Not pooled
	Neglect	2	1.35	1.17	1.55	0.28	0.00	0.60
	***Any cardiovascular disease***							
	Physical abuse	4	1.57	1.11	2.22	6.78	55.75	0.08
	Neglect	1	1.37	0.99	1.91	Not pooled	Not pooled	Not pooled
	**Type 2 diabetes**							
	Physical abuse	11	1.01	0.79	1.29	41.26	75.76	<0.01
	Emotional abuse	3	1.19	0.74	1.93	10.45	80.86	0.01
	Neglect	14	1.11	0.97	1.26	16.37	20.57	0.23
	**Respiratory diseases**							
	***Asthma***							
	Physical abuse	2	1.74	1.15	2.62	0.14	0.00	0.71
	***Asthma (hazard ratio)***							
	Physical abuse	1	1.92	1.32	2.81	Not pooled	Not pooled	Not pooled
	Neglect	1	1.02	0.70	1.49	Not pooled	Not pooled	Not pooled
	***Bronchitis/emphysema***							
	Physical abuse	3	1.39	1.19	1.62	0.91	0.00	0.63
	***Any respiratory disease***							
	Physical abuse (sometimes)	1	1.42	0.91	2.22	Not pooled	Not pooled	Not pooled
	Physical abuse (frequent)	1	1.09	0.78	1.52	Not pooled	Not pooled	Not pooled
**Other physical health outcomes**	**Ulcers**							
	Physical abuse	7	1.71	1.44	2.02	5.69	0.00	0.46
	Neglect	2	1.26	0.56	2.83	0.44	0.00	0.51
	**Headache/migraine**							
	Physical abuse	6	1.42	1.24	1.62	5.00	0.04	0.54
	Emotional abuse	1	1.60	1.40	1.70	Not pooled	Not pooled	Not pooled
	Neglect	1	3.11	0.31	30.80	Not pooled	Not pooled	Not pooled
	**Headache/migraine (hazard ratio)**							
	Physical abuse	1	1.64	1.44	1.88	Not pooled	Not pooled	Not pooled
	Neglect	1	1.21	1.02	1.43	Not pooled	Not pooled	Not pooled
	**Neurological disorders**							
	Physical abuse	3	2.19	1.30	3.69	0.55	0.00	0.76
	Neglect	3	2.07	0.99	4.32	0.08	0.00	0.96
	**Cancer**							
	Physical abuse	2	1.26	0.97	1.65	1.43	30.28	0.23
	**Arthritis**							
	Physical abuse	4	1.52	1.28	1.80	1.30	0.00	0.94
	Neglect	2	1.70	1.06	2.73	0.06	0.00	1.00
	**Arthritis (hazard ratio)**							
	Physical abuse	1	1.42	1.22	1.66	Not pooled	Not pooled	Not pooled
	Neglect	1	1.29	1.08	1.55	Not pooled	Not pooled	Not pooled
**Lifestyle risk factors**	**Tobacco smoking**							
	Physical abuse	19	1.55	1.09	2.21	161.75	88.87	<0.01
	Emotional abuse	6	1.70	1.55	1.87	2.38	0.00	0.79
	Neglect	2	1.20	0.98	1.48	0.63	0.00	0.43
	**Hypertension**							
	Physical abuse	6	1.16	0.94	1.44	5.64	11.33	0.34
	Neglect	4	1.04	0.78	1.39	1.16	0.00	0.76
	**Obesity**							
	Physical abuse	11	1.32	1.06	1.64	37.54	73.36	<0.01
	Emotional abuse	5	1.24	1.13	1.36	6.95	42.48	0.14
	Neglect	18	1.07	0.97	1.19	44.68	61.95	<0.01
	**Low exercise**							
	Physical abuse	1	1.04	0.86	1.26	Not pooled	Not pooled	Not pooled

**Table 7:** Summary of review findings on health consequences of child non-sexual maltreatment for disorders where data were insufficient to include in meta-analyses.

Health Outcome and Type of Maltreatment	OR	95% CI Lower Bound	95% CI Upper Bound
**Allergy ** [**153**]			
Physical abuse	1.38	1.06	1.78
**Anaemia ** [**33**]			
Physical abuse	0.56	0.23	1.34
Neglect	0.59	0.37	0.95
**Underweight/malnutrition ** [**33**]			
Physical abuse	3.16	1.53	6.50
Neglect	1.39	0.87	2.21
**Hepatitis C ** [**33**]			
Physical abuse	0.99	0.30	3.26
Neglect	1.18	0.59	2.38
**Tuberculosis ** [**33**]			
Physical abuse	0.75	0.07	8.58
Neglect	1.18	0.32	4.39
**Hearing loss ** [**33**]			
Physical abuse	2.37	0.68	8.26
Neglect	1.72	0.74	4.01
**Oral health ** [**33**]			
Physical abuse	0.70	0.37	1.35
Neglect	1.07	0.72	1.59
**Vision problems ** [**33**]			
Physical abuse	0.58	0.29	1.17
Neglect	1.17	0.76	1.78
**Diarrhoea (prevalence ratio) ** [**99**]			
Physical abuse	1.13	0.81	1.59
**Uterine leiomyoma ** [**100**]			
Physical abuse—mild	1.09	1.03	1.15
Physical abuse—moderate	1.10	1.04	1.15
Physical abuse—severe	1.16	1.07	1.25
**Back pain (prevalence ratio) ** [**99**]			
Physical abuse	1.03	0.84	1.26
**Chronic spinal pain (hazard ratio) ** [**150**]			
Physical abuse	1.61	1.43	1.82
Neglect	1.33	1.15	1.34
**Schizophrenia ** [**148**]			
Physical abuse	5.81	2.31	14.63
Emotional abuse	12.24	4.82	31.09
**Breast cancer (incidence rate ratio) ** [**175**]			
Physical abuse	1.01	0.88	1.17

### Mental Disorders

Physically abused (OR = 1.54; 95% CI 1.16–2.04), emotionally abused (OR = 3.06; 95% CI 2.43–3.85), and neglected (OR = 2.11; 95% CI 1.61–2.77) individuals were found to have a higher risk of developing depressive disorders than non-abused individuals (Table 4; Figures S1, S2, S3). The test for heterogeneity was highly significant, with *p*<0.01 for both abuse types and neglect. Funnel plots indicate the possibility of publication bias for physical abuse, as it appears that some smaller, less precise studies have a greater effect size than the larger studies, and there are no smaller studies to the left (negative) side of the graph, suggesting that some negative studies may never have been published (Figure S4).

For physical abuse, emotional abuse, and neglect, OR estimates in males were higher than in females, but the difference was not statistically significant (Table S1). The odds of developing depressive disorders with exposure to physical abuse were greatest in prospective studies. Although the OR point estimate was higher in subgroup analyses of studies where exposure to physical abuse was court-substantiated by official records—which would include the more severe cases of abuse (OR = 2.41; 95% CI 1.32–4.41)—compared with self-reported physical abuse (OR = 1.56; 95% CI 1.11–2.19) and physical punishment (OR = 1.20; 95% CI 0.88–1.61), the 95% CIs were overlapping, and these differences were not statistically significant. There was a stronger association between physical abuse and a diagnosis of major depressive disorder using structured interviews (OR = 1.82; 95% CI 1.44–2.30) than when depressive disorders were diagnosed by symptom scales (OR = 1.52; 95% CI 1.03–2.24), but again these differences were not statistically significant (Table S1). Restricting the physical abuse analysis to studies from high-income countries increased the odds of developing depressive disorders to 1.58 (95% CI 1.18–2.12), but the association was not significant in low-to-middle-income countries (Table S1).

However, for neglect in childhood, similar odds of developing depressive disorders were observed in high- and low-to-middle-income countries. Data from two studies suggest a dose–response relationship, with depression more likely with frequent neglect compared with neglect that occurred only sometimes in childhood [13],[22]. A dose–response relationship was also reported for emotional abuse and depressive disorders, but not for physical abuse and depressive disorders (Table S1).

Physical abuse (OR = 1.51; 95% CI 1.27–1.79), emotional abuse (OR = 3.21; 95% CI 2.05–5.03), and neglect (OR = 1.82; 95% CI 1.51–2.20) were associated with a significantly increased risk of anxiety disorders (Figures S5, S6, S7, S8). For physical abuse, significant associations were also observed with post-traumatic stress disorder (PTSD) and panic disorder diagnoses (Table S2). A dose–response relationship was observed with physical abuse but not with emotional abuse and neglect [22], with anxiety disorders more likely with frequent physical abuse than with abuse that occurred only sometimes in childhood (Table S2). Physical abuse, emotional abuse, and neglect were also associated with an almost 3-fold increased risk of developing eating disorders (Figures S9, S10, S11, S12), and physical abuse was associated with a 5-fold increased risk of developing bulimia nervosa meeting Diagnostic and Statistical Manual of Mental Disorders (DSM) diagnostic criteria. Most of the evidence came from retrospective studies, and only one prospective study [23] reported a strong association with neglect in childhood (Table S3). A dose–response relationship was also observed, with bulimia nervosa more likely with more severe and repeated physical abuse [24] (Table S3).

Physical abuse and neglect were also associated with a doubling of the odds of childhood behavioural and conduct disorders (Figures S13, S14, S15). With respect to physical abuse, higher odds of developing conduct and childhood behavioural disorders were observed in prospective than in retrospective studies, but differences were not statistically significant. Studies with non-representative samples had significantly increased effect size for the association between physical abuse and childhood behavioural problems and conduct disorder (OR = 5.98; 95% CI 2.73–13.10) compared with population-based studies (OR = 2.02; 95% CI 1.58–2.58) (Table S4).

Physical abuse significantly increased the risk of alcohol problem drinking (risky drinking, alcohol abuse/dependence, binge drinking) (OR = 1.26; 95% CI 1.03–1.55) (Figure S16) and non-problem drinking (current or ever alcohol use), but the effect did not persist in prospective studies (Table S5). In a subgroup analysis, physical abuse was also significantly associated with a diagnosis of alcohol abuse/dependence meeting DSM criteria (OR = 1.40; 95% CI 1.21–1.64) (Table S5). Alcohol problem drinking was also associated with emotional abuse (OR = 1.27; 95% CI 1.11–1.46) (Figure S17) but not with neglect in childhood (OR = 1.09; 95% CI 0.87–1.35) (Figure S18). For alcohol problems, there was no evidence of a dose–response relationship with respect to frequency of abuse and neglect (Table S5) [13]. Gender differences were observed, with the effect of physical abuse on alcohol problems stronger among males, and with females at an increased risk of alcohol problem drinking with exposure to neglect in childhood, but with overlapping confidence intervals (Table S5). Publication bias did not appear to play a role in the association between physical abuse and alcohol problem drinking (Figure S19).

Although primary analyses suggest an increased risk of drug use associated with physical abuse (OR = 1.92; 95% CI 1.67–2.20), emotional abuse (OR = 1.41; 95% CI 1.11–1.79), and neglect (OR = 1.36; 95% CI 1.21–1.54) (Figures S20, S21, S22, S23), there was only borderline significance in prospective studies, with a stronger consistent association observed in retrospective studies, albeit with overlapping confidence intervals (Table S6). A dose–response relationship between emotional abuse and neglect and drug use was not consistently seen.

Physically abused (OR = 3.00; 95% CI 2.07–4.33), emotionally abused (OR = 3.08; 95% CI 2.42–3.93), and neglected (OR = 1.85; 95% CI 1.25–2.73) individuals had a significantly increased risk of suicidal behaviour compared with non-abused individuals (Table 4). These significant associations continued in subgroup analyses by type of suicidal behaviour, with physically abused (OR = 3.40; 95% CI 2.17–5.32), emotionally abused (OR = 3.37; 95% CI 2.44–4.67), and neglected (OR = 1.95; 95% CI 1.13–3.37) individuals at a significantly increased risk of suicide attempt (Figures S24, S25, S26, S27) and suicide ideation (Table S7). There were no prospective studies investigating non-sexual child maltreatment and suicide attempt or ideation. Only one prospective study [25] was found investigating the association between self-inflicted injuries and exposure to physical abuse and neglect. Six studies [13],[26]–[30] presented the results by gender for physical abuse and suicide attempt and ideation, but no statistically significant differences were observed. One study showed that exposure to frequent childhood neglect was more strongly associated with suicidal behaviour than exposure to neglect that occurred sometimes [13] (Table S7).

### Sexually Transmitted Infections and Risky Sexual Behaviour

Physically abused (OR = 1.78; 95% CI 1.50–2.10), emotionally abused (OR = 1.75; 95% CI 1.49–2.04), and neglected (OR = 1.57; 95% CI 1.39–1.78) individuals were found to have a significantly higher risk of sexually transmitted infections (STIs) and/or risky sexual behaviour than non-abused individuals (Table 5; Figures S28, S29, S30, S31). For physical abuse and neglect, the association with STIs and risky sexual behaviour was significant in prospective and retrospective studies (Table S8). HIV infection was about twice as common in physically abused (OR = 2.51; 95% CI 1.16–5.42), emotionally abused (OR = 1.82; 95% CI 1.34–2.47), and neglected (OR = 2.50; 95% CI 0.77–8.15) individuals as in controls, although for neglect the difference did not reach conventional levels of significance, probably because of weak statistical power. Physical abuse was also associated with an increased risk of other STIs (OR = 1.53; 95% CI 1.13–2.07) and risky sexual behaviour (OR = 1.95; 95% CI 1.58–2.40) (Table 5). A dose–response relationship was observed for HIV infection, with a larger effect size reported with more frequent physical and emotional abuse in childhood [13] (Table S8).

### Chronic Diseases, Lifestyle Risk Factors, and Other Physical Health Outcomes

With regard to obesity, a significantly increased risk was observed for physical (OR = 1.32; 95% CI 1.06–1.64) and emotional abuse (OR = 1.24; 95% CI 1.13–1.36) but not for neglect (OR = 1.07; 95% CI 0.97–1.19) in the primary analysis (Figures S32, S33, S34, S35). Subgroup analysis by assessment of outcome indicated that neglect was associated with a higher risk of developing self-reported obesity, but there was no association with obesity defined by waist circumference or body mass index (BMI) measurements (Table S9). In the subgroup analysis by ascertainment of exposure to physical abuse, there was a strong association with obesity in one prospective study, but the magnitude of the effect was reduced in retrospective studies (Table S9). A dose–response relationship between physical and emotional abuse and obesity has been observed [31] (Table S9).

Physical (OR = 1.78; 95% CI 1.26–2.52) (Figure S36) and emotional abuse (OR = 1.65; 95% CI 1.46–1.87) (Figure S37) were associated with a significantly increased risk of current smoking, but the association was not significant for neglect in childhood (OR = 1.20; 95% CI 0.98–1.48). One study showed a dose response, with smoking more likely with physical abuse that occurred 3–5 times than with abuse that occurred 1–2 times, but this relationship did not continue into those who had been abused more than six times compared with those who had been abused 3–5 times [32] (Table S10).

Forty-two studies investigated the relationship between non-sexual child maltreatment and lifestyle risk factors, chronic diseases, and other physical health outcomes in adulthood. There is suggestive evidence of a significant association between child physical abuse and arthritis, ulcers, and headache/migraine in adulthood. However, for most other outcomes, including type 2 diabetes (Table S11; Figures S39, S40, S41, S42), hypertension, low exercise, cardiovascular diseases, respiratory diseases, neurological disorders, and cancer, these associations were mostly weak and inconsistent, with little adjustment for lifetime confounders. Pooled estimates were statistically significant in only a limited number of cases (Table 6). A recent prospective investigation of a group of individuals with documented histories of child abuse and neglect followed into middle adulthood provides some evidence that child abuse and neglect may increase the risk of a range of directly measured physical health outcomes after controlling for mental health problems, substance use, smoking, and BMI [33] (Table 7). However, there were insufficient studies examining the association between non-sexual child maltreatment and some of these health outcomes, including anaemia, underweight/malnutrition, hepatitis C, tuberculosis, hearing loss, vision loss, oral health, diarrhoea, allergies, uterine leiomyoma, back pain, breast cancer, and schizophrenia, to undergo meta-analysis (Table 7).

## Discussion

To the best of our knowledge, this article presents the first systematic review and meta-analysis of published studies assessing the association between non-sexual child maltreatment and mental and physical health outcomes. We identified 124 studies that examined the association between physical abuse, emotional abuse, and neglect in childhood and various health outcomes.

### Does Non-Sexual Child Maltreatment Cause Adverse Health Outcomes?

Evidence for a causal relationship between non-sexual child maltreatment and health outcomes was evaluated within the Bradford Hill framework on the grounds of the following important criteria: strength and consistency of the association, the temporal relationship of the association, evidence of a biological gradient or dose–response relationship, biological plausibility, and consideration of alternate explanations [34] (Table S12).

### Temporality

Both prospective and retrospective studies consistently showed an association between exposure to child physical abuse, emotional abuse, and neglect and adverse health outcomes. The availability of prospective studies provides conclusive evidence of a temporal relationship between exposure to non-sexual child maltreatment and the later development of mental health outcomes, drug use, and STIs and risky sexual behaviour, as in these studies abuse and neglect preceded the onset of health problems in adulthood.

However, only 16 studies were prospective, while the majority of the studies were cross-sectional and relied on adult retrospective report of abuse and neglect in childhood. By definition, these studies cannot prove a temporal relationship between exposure to child maltreatment and the onset of health outcomes. Furthermore, retrospective, self-reported information regarding abuse in childhood may be subject to recall bias, where those with adjustment problems may be more prone to recall or disclose exposure to abuse and neglect. In many cases participants were asked to report on events that would have occurred many years before, and the issue of potentially unreliable recall threatens the validity of the published literature on child maltreatment. At least with respect to child sexual abuse, evidence suggests moderate to good consistency of reports over time [35]. It has also been suggested that biases are probably towards under-reporting rather than over-reporting of abuse [36]. Nevertheless, given that retrospective reports were often the only measure of abuse available, particularly with regard to emotional abuse, we accepted these within the context of the limitations stated.

Although the strength of prospective studies includes the temporal ordering of maltreatment and subsequent health outcomes, with an objective measurement of exposure to abuse, these studies are usually conducted in non-representative samples. Official cases of abuse may only detect those who come to professional attention, and this may alter the strength of the association between non-sexual child maltreatment and adult morbidity. These official cases are also generally skewed towards the lower end of the socioeconomic spectrum and may not be generalisable to child abuse and neglect cases that occur in middle- or upper-class children [33]. Those participants who have been identified by child protection agencies as having been exposed to physical abuse or neglect may have received interventions to prevent later pathology. Furthermore, some individuals in the “never maltreated” category may actually have experienced maltreatment, given that child maltreatment tends to be under-reported. The validity of the various study designs to investigate the long-term health consequences of child maltreatment has been a source of ongoing debate [37],[38]. In this meta-analysis we have included prospective and retrospective studies. The subgroup analyses show that with both methodologies there is robust evidence of a significant association between child non-sexual maltreatment and various health outcomes.

### Strength of the Association

Associations between child physical abuse, emotional abuse, and neglect and mental disorders, drug use, and suicidal behaviour have been reported in prospective studies and/or large population-based studies. The strength of the relationship between abuse and mental disorders was generally reduced when the effects of important mediating variables were taken into account. Despite some variability, overall, child physical abuse, emotional abuse, and neglect were found to approximately double the likelihood of adverse mental health outcomes when combined in a meta-analysis.

### Consistency of the Association

As shown in the forest plots of the effects by study, there was strong consistency and agreement in the estimated effect measures across studies, particularly for neglect and physical abuse, although we suspect publication bias for some of the outcomes. Risk estimates were comparable across different types of samples, for both non-representative and representative populations (Tables S1, S2, S3, S4 and S6, S7, S8). The findings persisted across different study designs, samples, and geographic regions investigated. It can be concluded that there is a highly consistent association between child physical abuse, emotional abuse, and neglect and adverse mental health outcomes, drug use, and STIs and risky sexual behaviour. We did not observe evidence of strong consistent associations for alcohol problems, chronic diseases, or lifestyle risk factors.

### Dose–Response Relationship

We found evidence of a dose–response relationship between adverse health outcomes and non-sexual child maltreatment, such that those experiencing more severe abuse or neglect were at greater risk of developing mental disorders than those experiencing less severe maltreatment [39]. In the Chapman et al. [40] study, increasing severity of childhood adversity corresponded with poorer mental health outcomes. Consistent dose–response relationships with repeated, frequent, or severe abuse have been reported for mental disorders and physical abuse [13],[24],[41] and emotional abuse and neglect [13],[22]. Furthermore, there is evidence to suggest that experiencing multiple types of maltreatment may carry more severe consequences, with those exposed to multiple types of abuse at increased odds of developing mental disorders [42],[43], and the risk increases with the magnitude of multiple abuse [44]. Dose–response relationships with repeated frequent or severe abuse have also been reported for STIs and physical and emotional abuse [13], obesity and emotional and physical abuse [31], and smoking and physical abuse [32].

### Plausibility

With respect to biological plausibility, animal models of mental disorders do not exist, making it particularly difficult to understand the underlying biological mechanisms. Progress in understanding has to be made by association and inference rather than experimental data [3]. There are nevertheless several potential mechanisms that may explain the observed association between abuse and neglect in childhood and increased risk of mental health problems. Neurobiological development can be physiologically altered by maltreatment during a child's early years, which can in turn negatively affect a child's physical, cognitive, emotional, and social growth, leading to psychological, behavioural, and learning problems that persist throughout the life course [45],[46]. Moreover, cumulative trauma may further increase risk [47], and some victims of abuse may try to manage the subsequent distress through the use of alcohol, prescription medication, tobacco, or other drugs.

There is emerging evidence that the origins of most adult disease are found among developmental and biological disruptions in childhood. These early life experiences can affect adult mental and physical health either by cumulative damage over time or by the biological embedding of adversities during sensitive developmental periods [48]. There is generally a lag of many years before early adverse experiences are expressed in the form of disease [48]. Andrews and colleagues concluded that despite the lack of a biological link between child sexual abuse and mental disorders, a causal relationship was plausible [3], and that child maltreatment is most likely a contributory cause that acts via other intermediates.

### Consideration of Alternate Explanations

It is important to note that the role of genes, environment, and gene–environment interactions in the causation of mental disorders is not well understood. Twin studies provide one of the best ways to examine the interplay between genetic and environmental influences [3], but to the best of our knowledge, these are only available for child sexual abuse. The relationship between abuse and neglect in childhood and subsequent health effects is complex. Although childhood abuse and neglect does result in adverse health outcomes, these outcomes are not independent of broader socioeconomic contexts. Lifestyle factors, access to health care, and neighbourhood characteristics may act as mediators between child abuse and neglect and long-term health consequences [49]–[51]. Exposure to child maltreatment often co-occurs within the context of other family dysfunction, social deprivation, and other environmental stressors that are also associated with mental disorders. Child maltreatment may be a marker of other family problems that together lead to the development of mental disorders. In addition, findings from many studies do not take into account the likely contribution of hereditary influences on the predisposition to mental disorders. Children of depressed parents may be at greater risk of depression through both exposure to maltreatment by their parents and genetic predisposition [43]. Hence, some of the effect of child abuse and neglect on mental disorders may still be explained by confounding. However, the effect of abuse on mental disorders remained significant in the majority of studies included in these meta-analyses after controlling for these co-occurring factors.

### Assessment of Causality

In summary, there was robust evidence of significant associations between exposure to non-sexual child maltreatment and increased likelihood of a range of mental disorders, suicide attempts, drug use, STIs, and risky sexual behaviour. An increase in the likelihood of alcohol problem use was not consistently seen. There is weak to limited evidence suggesting a relationship between non-sexual child maltreatment and certain physical disorders and risk factors (Table 8), but more research is required to confirm these relationships.

**Table 8:** Summary of the strength of the evidence for related health outcomes.

Robust Evidence	Weak/Inconsistent Evidence	Limited Evidence
**Physical abuse**		
Depressive disorders	Cardiovascular diseases	Allergies
Anxiety disorders	Type 2 diabetes	Cancer
Eating disorders	Obesity	Neurological disorders
Childhood behavioural/conduct disorders	Hypertension	Underweight/malnutrition
Suicide attempt	Smoking	Uterine leiomyoma
Drug use	Ulcers	Chronic spinal pain
STIs/risky sexual behaviour	Headache/migraine	Schizophrenia
	Arthritis	Bronchitis/emphysema
	Alcohol problems	Asthma
**Emotional abuse**		
Depressive disorders	Eating disorders	Cardiovascular diseases
Anxiety disorders	Type 2 diabetes	Schizophrenia
Suicide attempt	Obesity	Headache/migraine
Drug use	Smoking	
STIs/risky sexual behaviour	Alcohol problems	
**Neglect**		
Depressive disorders	Eating disorders	Arthritis
Anxiety disorders	Childhood behavioural/conduct disorders	Headache/migraine
Suicide attempt	Cardiovascular diseases	Chronic spinal pain
Drug use	Type 2 diabetes	Smoking
STIs/risky sexual behaviour	Alcohol problems	
	Obesity	

### Study Limitations

Although these findings and conclusions seem to be relatively consistent and robust, they should be interpreted in light of a number of limitations of our analysis.

This meta-analysis may be subject to publication bias because non-significant findings are less likely to be published [52]. This problem is increased when statistical models are employed because often only significant estimates are reported in many studies. This may result in the association between child abuse and neglect and outcomes being overstated, particularly for depressive disorders and anxiety, where publication bias may have played a role. For some of the other conditions there were too few studies to make conclusions with respect to publication bias.

The analysis also suffers from inconsistencies in how child abuse and neglect are defined and measured across the studies, as shown in Table 3. In studies using child protection records, exposure to physical abuse was defined to include injuries such as bruises, welts, burns, abrasions, lacerations, wounds, cuts, and fractures. Some studies used the Barnett-Cicchetti Maltreatment Classification System [53] which defines physical abuse as a caregiver or responsible adult inflicting physical injury upon a child by other than accidental means. In other studies physical abuse was defined as having been hit, kicked, or punched so hard that the individual had marks or bruising or needed medical attention. Some studies referred to physical punishment [13],[54],[55] and corporal punishment [56], which may exclude more severe physical abuse, as well as physical assault by caregivers [57]. Emotional abuse definitions also varied considerably and included verbal abuse and being humiliated by a caregiver. Most studies involving neglect referred simply to “neglect”, while others distinguished between physical and emotional neglect. Similarly, definitions of childhood were not consistent across studies. The complexity of defining and measuring child abuse has been noted in several studies [58]–[60].

Measurement bias with respect to health outcomes and the questionable reliability of self-reported data may also have affected the results. We dealt with this issue in the meta-analysis by adjusting the quality score and performing subgroup analyses. For mental disorders, studies using well-validated and standardised diagnostic instruments were assigned a higher quality score than studies using self-report symptom scales.

Another limitation of meta-analyses of observational studies is that, since individuals cannot be randomly allocated to case groups, the influence of confounding variables cannot be fully evaluated. While most studies presented multivariable adjusted ORs controlling for a range of socio-demographic and study design variables, a few studies presented unadjusted associations between child maltreatment and health outcomes, or adjusted for age and sex only. We again dealt with this issue in our meta-analysis by adjusting the quality score of studies with inadequate control for confounding and by carrying out separate analyses depending on data availability. Some studies also statistically controlled for exposure to other forms of maltreatment by including the different types of abuse in the same model in order to determine the independent contribution of each abuse type. Generally, in studies presenting results from various unadjusted and adjusted models, the association between abuse and physical and mental health outcomes was attenuated when controlling for the effects of mediating variables [61]–[72] and other forms of abuse [73]–[79]. However, findings from a recent prospective cohort study indicate that for some physical health outcomes additional control for socioeconomic status, unhealthy behaviour, smoking, and mental health problems seems to play varying roles in attenuating or intensifying these complex relationships [33]. Furthermore, we cannot exclude that residual confounding or unmeasured potential confounders may still remain. Despite evidence of weak associations between non-sexual child maltreatment and chronic diseases, further studies are needed that ensure adequate adjustment for lifetime confounders, because the attributable burden would be appreciable.

Significant heterogeneity exists in the primary analysis of physical and emotional abuse, even after our attempts to control for study quality in quality effects models, and the heterogeneity remained significant in most of the subgroup analyses. Given this situation, combining the effects may not be justified. With respect to neglect, pooled estimates in primary and subgroup analyses did not show significant heterogeneity for many outcomes.

### Recommendations

Inconsistencies in the measurement and definition of child maltreatment highlight the importance of international efforts to standardise studies to enhance the comparability of findings. These include defining the cutoff age for childhood (0–18 y, as specified by the United Nations), and breaking this period into smaller age bands that can reflect age-specific patterns [5]. Researchers should select methodologies and instruments with international comparisons in mind. Identical questionnaires, research designs, and interviewing techniques should ideally be used for surveys in different countries [5]. In reality, however, all survey methods will require at least some adaptation to local conditions, and efforts to ensure comparability should involve choosing definitions of abuse and neglect, and questionnaire items, that represent an advanced level of knowledge [80]. To minimise how participants' subjective perceptions and definitions shape the answers, it is recommended that self-report studies clearly specify the behaviours and experiences being investigated, and that each sub-type of abuse and neglect is explored using multiple behaviourally specific questions, instead of a single-item “label question” [81].

Examples of international efforts to increase comparability across studies include the WHO's establishment of a global adverse childhood experiences research network, and the International Society for Prevention of Child Abuse and Neglect's Child Abuse Screening Tools (ICAST). The WHO network has developed an international version of the Adverse Childhood Experiences (ACE) questionnaire (the ACE International Questionnaire), for administration to people aged 18 y and older, which is currently being validated through trial implementation as part of broader health surveys in several countries [82]. The ICAST initiative has involved the development of three instruments that ask parents about their use of different behaviours for discipline, young adults (18–24 y) about their exposure to child abuse and neglect in childhood, and older children about their own recent experiences of violence [83].

Child maltreatment deserves increased investment in preventive and treatment strategies. Currently, there is a paucity of evidence-based interventions to reduce child maltreatment. Further research is urgently needed to identify programs that reduce the prevalence of child maltreatment, thereby alleviating an important risk factor for later health problems. Evidence-based systemic interventions that improve parenting strategies and family functioning may be more effective and economical than attempting to treat the wide-ranging deleterious health outcomes in adulthood that arise from maltreatment in the early years of life [48],[84].

A broad range of protective factors have been identified that assist in promoting resilience in children exposed to adversity. Self control, problem-solving skills, secure relationships with caregivers, and safe schools and neighbourhoods are known to reduce the risk of adverse consequences in children exposed to trauma [85],[86]. There is mounting evidence that exposure to childhood adversity interacting with particular genetic dispositions such as the short allele of the serotonin transporter gene [87] and genes involved in the regulation of the hypothalamic–pituitary axis [88],[89] can result in problems with stress regulation and increased risk of anxiety and depression. Epigenetic changes have also been postulated as a mechanism by which transgenerational resilience or vulnerability may occur [90]. In spite of the increased knowledge in this field, it remains a challenge to translate this research into interventions at a population level that can reduce the vulnerability of children exposed to maltreatment [91].

### Conclusion

This overview of the evidence suggests a causal relationship between non-sexual child maltreatment and a range of mental disorders, drug use, suicide attempts, sexually transmitted infections, and risky sexual behaviour. There is also emerging evidence that neglect in childhood may be as harmful as physical and emotional abuse. Although these conclusions have been drawn before from single empirical studies, in this article they are demonstrated in aggregate quantitative effects, to our knowledge for the first time.

This review contributes to a better understanding and measurement of the non-injury health impacts of child maltreatment globally and enables quantification of the burden attributable to physical and emotional abuse and neglect at the population level using comparative risk assessment methodology [92]. All forms of child maltreatment should be considered as part of the cluster of interpersonal violence risk factors in future global comparative risk assessments. Attributable burden is likely to be substantial, given the high prevalence of these forms of child maltreatment, the strong associations reported in our analysis, and the fact that related health outcomes are among the leading causes of disease burden globally. Despite the magnitude of the problem and increasing awareness of its high social costs, preventing child maltreatment is not a political priority in most countries. It is imperative that epidemiology and public health approaches find their proper place at the forefront of national and international efforts to understand and prevent child maltreatment [93].

## Supporting Information

Figure S1 **Forest plot for quality-effect meta-analysis of the association between physical abuse and depressive disorders.** Studies are represented by symbols, the area of which is proportional to the study's weight in the analysis. Output for ORs is set to the (natural) log scale.
(TIF)

Figure S2 **Forest plot for quality-effect meta-analysis of the association between emotional abuse and depressive disorders.** Studies are represented by symbols, the area of which is proportional to the study's weight in the analysis. Output for ORs is set to the (natural) log scale.
(TIF)

Figure S3 **Forest plot for quality-effect meta-analysis of the association between neglect and depressive disorders.** Studies are represented by symbols, the area of which is proportional to the study's weight in the analysis. Output for ORs is set to the (natural) log scale.
(TIF)

Figure S4 **Funnel plots to aid assessment of publication bias for depressive disorders and physical abuse.**
(TIF)

Figure S5 **Forest plot for quality-effect meta-analysis of the association between physical abuse and anxiety.** Studies are represented by symbols, the area of which is proportional to the study's weight in the analysis. Output for ORs is set to the (natural) log scale.
(TIF)

Figure S6 **Forest plot for quality-effect meta-analysis of the association between emotional abuse and anxiety.** Studies are represented by symbols, the area of which is proportional to the study's weight in the analysis. Output for ORs is set to the (natural) log scale.
(TIF)

Figure S7 **Forest plot for quality-effect meta-analysis of the association between neglect and anxiety.** Studies are represented by symbols, the area of which is proportional to the study's weight in the analysis. Output for ORs is set to the (natural) log scale.
(TIF)

Figure S8 **Funnel plot to aid assessment of publication bias for anxiety and physical abuse.**
(TIF)

Figure S9 **Forest plot for quality-effect meta-analysis of the association between physical abuse and eating disorders.** Studies are represented by symbols, the area of which is proportional to the study's weight in the analysis. Output for ORs is set to the (natural) log scale.
(TIF)

Figure S10 **Forest plot for quality-effect meta-analysis of the association between emotional abuse and eating disorders.** Studies are represented by symbols, the area of which is proportional to the study's weight in the analysis. Output for ORs is set to the (natural) log scale.
(TIF)

Figure S11 **Forest plot for quality-effect meta-analysis of the association between neglect and eating disorders.** Studies are represented by symbols, the area of which is proportional to the study's weight in the analysis. Output for ORs is set to the (natural) log scale.
(TIF)

Figure S12 **Funnel plot to aid assessment of publication bias for eating disorders and physical abuse.**
(TIF)

Figure S13 **Forest plot for quality-effect meta-analysis of the association between physical abuse and conduct/childhood behavioural disorders.** Studies are represented by symbols, the area of which is proportional to the study's weight in the analysis. Output for ORs is set to the (natural) log scale.
(TIF)

Figure S14 **Forest plot for quality-effect meta-analysis of the association between neglect and conduct/childhood behavioural disorders.** Studies are represented by symbols, the area of which is proportional to the study's weight in the analysis. Output for ORs is set to the (natural) log scale.
(TIF)

Figure S15 **Funnel plot to aid assessment of publication bias for childhood behavioural/conduct disorders and physical abuse.**
(TIF)

Figure S16 **Forest plot for quality-effect meta-analysis of the association between physical abuse and alcohol problem drinking.** Studies are represented by symbols, the area of which is proportional to the study's weight in the analysis. Output for ORs is set to the (natural) log scale.
(TIF)

Figure S17 **Forest plot for quality-effect meta-analysis of the association between emotional abuse and alcohol problem drinking.** Studies are represented by symbols, the area of which is proportional to the study's weight in the analysis. Output for ORs is set to the (natural) log scale.
(TIF)

Figure S18 **Forest plot for quality-effect meta-analysis of the association between neglect and alcohol problem drinking.** Studies are represented by symbols, the area of which is proportional to the study's weight in the analysis. Output for ORs is set to the (natural) log scale.
(TIF)

Figure S19 **Funnel plot to aid assessment of publication bias for alcohol problem drinking and physical abuse.**
(TIF)

Figure S20 **Forest plot for quality-effect meta-analysis of the association between physical abuse and drug use.** Studies are represented by symbols, the area of which is proportional to the study's weight in the analysis. Output for ORs is set to the (natural) log scale.
(TIF)

Figure S21 **Forest plot for quality-effect meta-analysis of the association between emotional abuse and drug use.** Studies are represented by symbols, the area of which is proportional to the study's weight in the analysis. Output for ORs is set to the (natural) log scale.
(TIF)

Figure S22 **Forest plot for quality-effect meta-analysis of the association between neglect and drug use.** Studies are represented by symbols, the area of which is proportional to the study's weight in the analysis. Output for ORs is set to the (natural) log scale.
(TIF)

Figure S23 **Funnel plot to aid assessment of publication bias for drug use and physical abuse.**
(TIF)

Figure S24 **Forest plot for quality-effect meta-analysis of the association between physical abuse and suicide attempt.** Studies are represented by symbols, the area of which is proportional to the study's weight in the analysis. Output for ORs is set to the (natural) log scale.
(TIF)

Figure S25 **Forest plot for quality-effect meta-analysis of the association between emotional abuse and suicide attempt.** Studies are represented by symbols, the area of which is proportional to the study's weight in the analysis. Output for ORs is set to the (natural) log scale.
(TIF)

Figure S26 **Forest plot for quality-effect meta-analysis of the association between neglect and suicide attempt.** Studies are represented by symbols, the area of which is proportional to the study's weight in the analysis. Output for ORs is set to the (natural) log scale.
(TIF)

Figure S27 **Funnel plot to aid assessment of publication bias for suicide attempt and physical abuse.**
(TIF)

Figure S28 **Forest plot for quality-effect meta-analysis of the association between physical abuse and sexually transmitted infections/risky sexual behaviour.** Studies are represented by symbols, the area of which is proportional to the study's weight in the analysis. Output for ORs is set to the (natural) log scale.
(TIF)

Figure S29 **Forest plot for quality-effect meta-analysis of the association between emotional abuse and sexually transmitted infections/risky sexual behaviour.** Studies are represented by symbols, the area of which is proportional to the study's weight in the analysis. Output for ORs is set to the (natural) log scale.
(TIF)

Figure S30 **Forest plot for quality-effect meta-analysis of the association between neglect and sexually transmitted infections/risky sexual behaviour.** Studies are represented by symbols, the area of which is proportional to the study's weight in the analysis. Output for ORs is set to the (natural) log scale.
(TIF)

Figure S31 **Funnel plot to aid assessment of publication bias for sexually transmitted infections/risky sexual behaviour and physical abuse.**
(TIF)

Figure S32 **Forest plot for quality-effect meta-analysis of the association between physical abuse and obesity.** Studies are represented by symbols, the area of which is proportional to the study's weight in the analysis. Output for ORs is set to the (natural) log scale.
(TIF)

Figure S33 **Forest plot for quality-effect meta-analysis of the association between emotional abuse and obesity.** Studies are represented by symbols, the area of which is proportional to the study's weight in the analysis. Output for ORs is set to the (natural) log scale.
(TIF)

Figure S34 **Forest plot for quality-effect meta-analysis of the association between neglect and obesity.** Studies are represented by symbols, the area of which is proportional to the study's weight in the analysis. Output for ORs is set to the (natural) log scale.
(TIF)

Figure S35 **Funnel plot to aid assessment of publication bias for obesity and neglect.**
(TIF)

Figure S36 **Forest plot for quality-effect meta-analysis of the association between physical abuse and current smoking.** Studies are represented by symbols, the area of which is proportional to the study's weight in the analysis. Output for ORs is set to the (natural) log scale.
(TIF)

Figure S37 **Forest plot for quality-effect meta-analysis of the association between emotional abuse and current smoking.** Studies are represented by symbols, the area of which is proportional to the study's weight in the analysis. Output for ORs is set to the (natural) log scale.
(TIF)

Figure S38 **Funnel plot to aid assessment of publication bias for current smoking and physical abuse.**
(TIF)

Figure S39 **Forest plot for quality-effect meta-analysis of the association between physical abuse and type 2 diabetes.** Studies are represented by symbols, the area of which is proportional to the study's weight in the analysis. Output for ORs is set to the (natural) log scale.
(TIF)

Figure S40 **Forest plot for quality-effect meta-analysis of the association between emotional abuse and type 2 diabetes.** Studies are represented by symbols, the area of which is proportional to the study's weight in the analysis. Output for ORs is set to the (natural) log scale.
(TIF)

Figure S41 **Forest plot for quality-effect meta-analysis of the association between neglect and type 2 diabetes.** Studies are represented by symbols, the area of which is proportional to the study's weight in the analysis. Output for ORs is set to the (natural) log scale.
(TIF)

Figure S42 **Funnel plot to aid assessment of publication bias for type 2 diabetes and neglect.**
(TIF)

Table S1 **Depressive disorders subgroup analyses.**
(DOC)

Table S2 **Anxiety disorders subgroup analyses.**
(DOC)

Table S3 **Eating disorders subgroup analyses.**
(DOC)

Table S4 **Childhood behavioural/conduct disorders subgroup analyses.**
(DOC)

Table S5 **Alcohol use subgroup analyses.**
(DOC)

Table S6 **Drug use subgroup analyses.**
(DOC)

Table S7 **Suicidal behaviour subgroup analyses.**
(DOC)

Table S8 **Sexually transmitted infections and risky sexual behaviour subgroup analyses.**
(DOC)

Table S9 **Obesity subgroup analyses.**
(DOC)

Table S10 **Tobacco smoking subgroup analyses.**
(DOC)

Table S11 **Type 2 diabetes subgroup analyses.**
(DOC)

Table S12 **Evaluation of the evidence for a causal relationship within the Bradford Hill framework for prospective and retrospective studies.**
(DOC)

Text S1 **PRISMA checklist.**
(DOC)

Text S2 **Review protocol.**
(DOC)

## Abbreviations

ACE: Adverse Childhood Experiences
BMI: body mass index
CI: confidence interval
DSM: Diagnostic and Statistical Manual of Mental Disorders
HSV2: herpes simplex virus type 2
OR: odds ratio
PTSD: post-traumatic stress disorder
STI: sexually transmitted infection
WHO: World Health Organization
