# Supplementary figures and images for: The Long-Term Health Consequences of Child Physical Abuse, Emotional Abuse, and Neglect: A Systematic Review and Meta-Analysis
Source: PLoS Med. 2012 Nov 27;9(11):e1001349. doi: 10.1371/journal.pmed.1001349 (PMC3507962; doi:10.1371/journal.pmed.1001349)

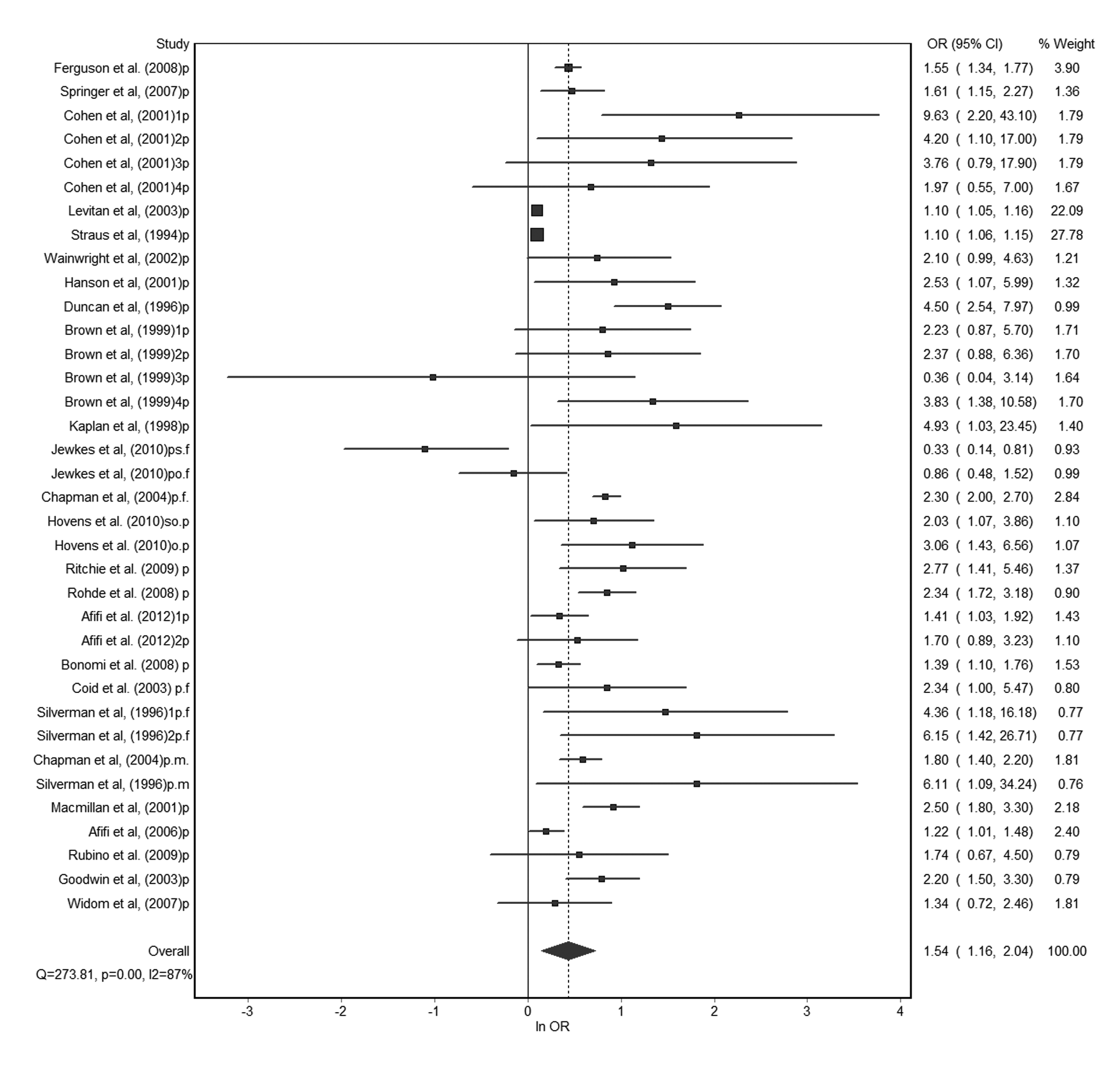

Supplement: Figure S1 — Forest plot for quality-effect meta-analysis of the association between physical abuse and depressive disorders. Studies are represented by symbols, the area of which is proportional to the study's weight in the analysis. Output for ORs is set to the (natural) log scale. (TIF) [file pmed.1001349.s001.tif]

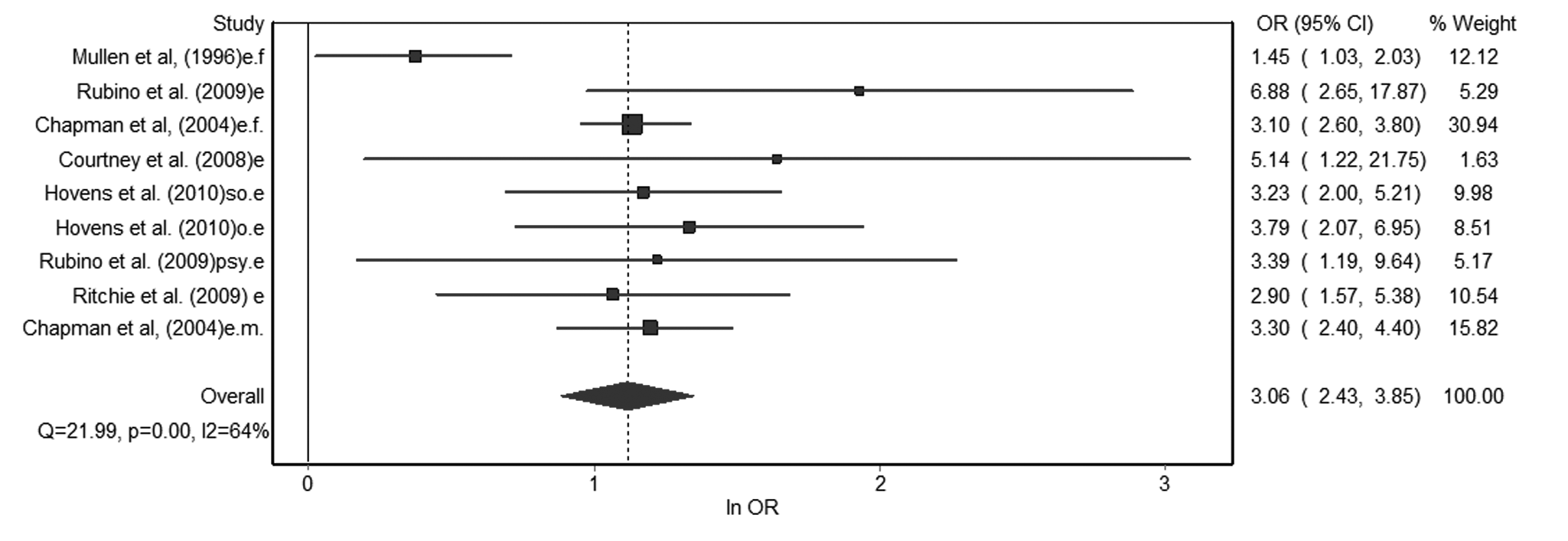

Supplement: Figure S2 — Forest plot for quality-effect meta-analysis of the association between emotional abuse and depressive disorders. Studies are represented by symbols, the area of which is proportional to the study's weight in the analysis. Output for ORs is set to the (natural) log scale. (TIF) [file pmed.1001349.s002.tif]

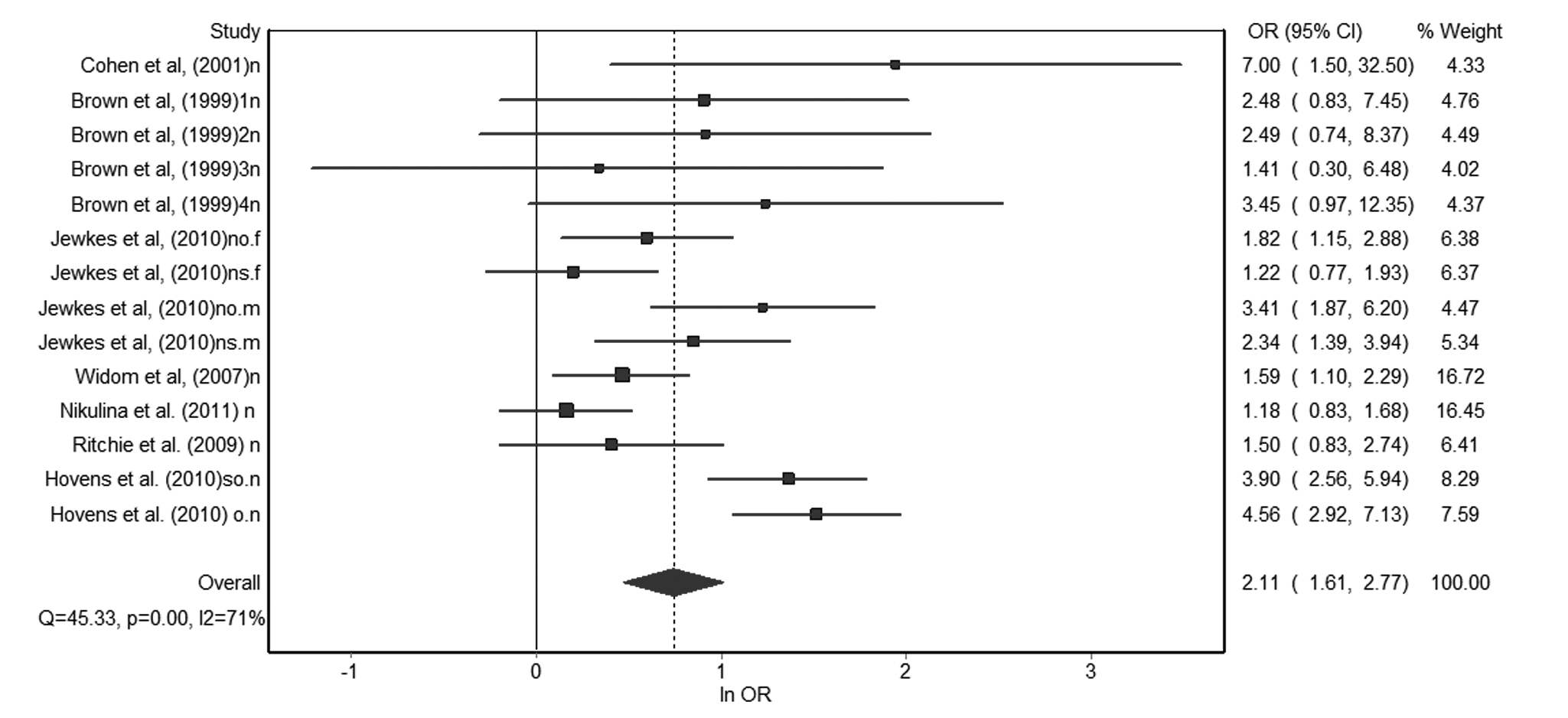

Supplement: Figure S3 — Forest plot for quality-effect meta-analysis of the association between neglect and depressive disorders. Studies are represented by symbols, the area of which is proportional to the study's weight in the analysis. Output for ORs is set to the (natural) log scale. (TIF) [file pmed.1001349.s003.tif]

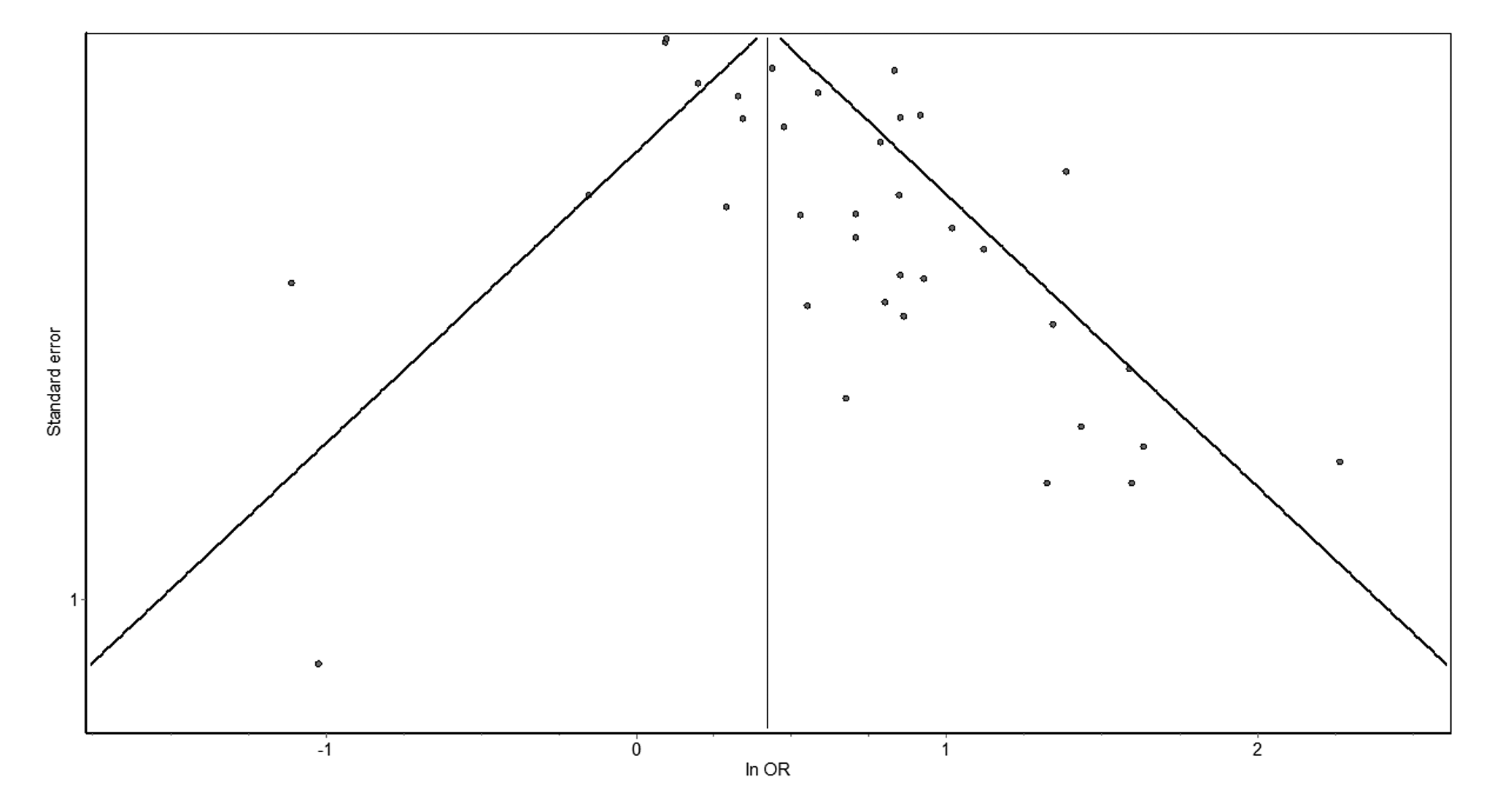

Supplement: Figure S4 — Funnel plots to aid assessment of publication bias for depressive disorders and physical abuse. (TIF) [file pmed.1001349.s004.tif]

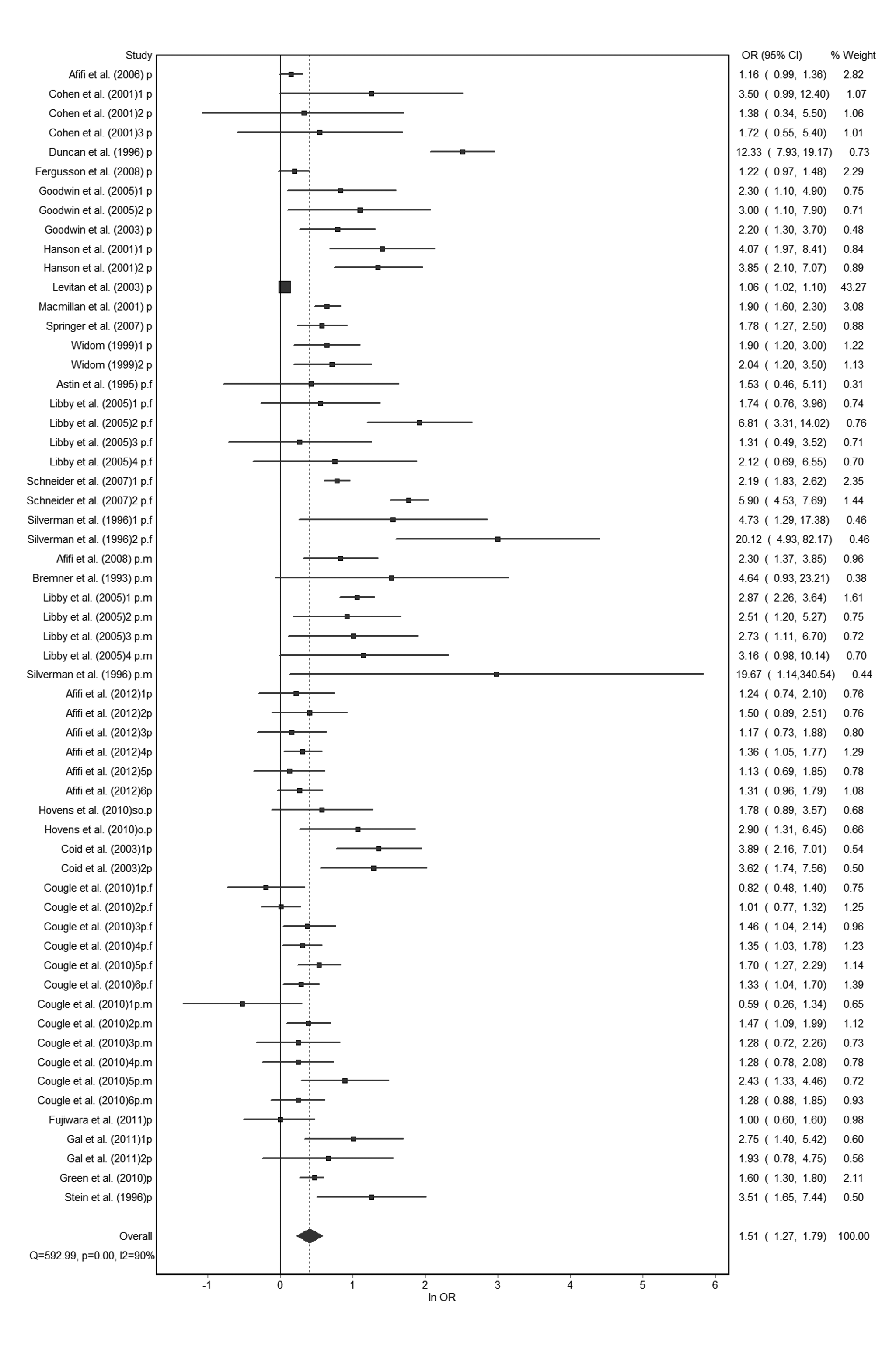

Supplement: Figure S5 — Forest plot for quality-effect meta-analysis of the association between physical abuse and anxiety. Studies are represented by symbols, the area of which is proportional to the study's weight in the analysis. Output for ORs is set to the (natural) log scale. (TIF) [file pmed.1001349.s005.tif]

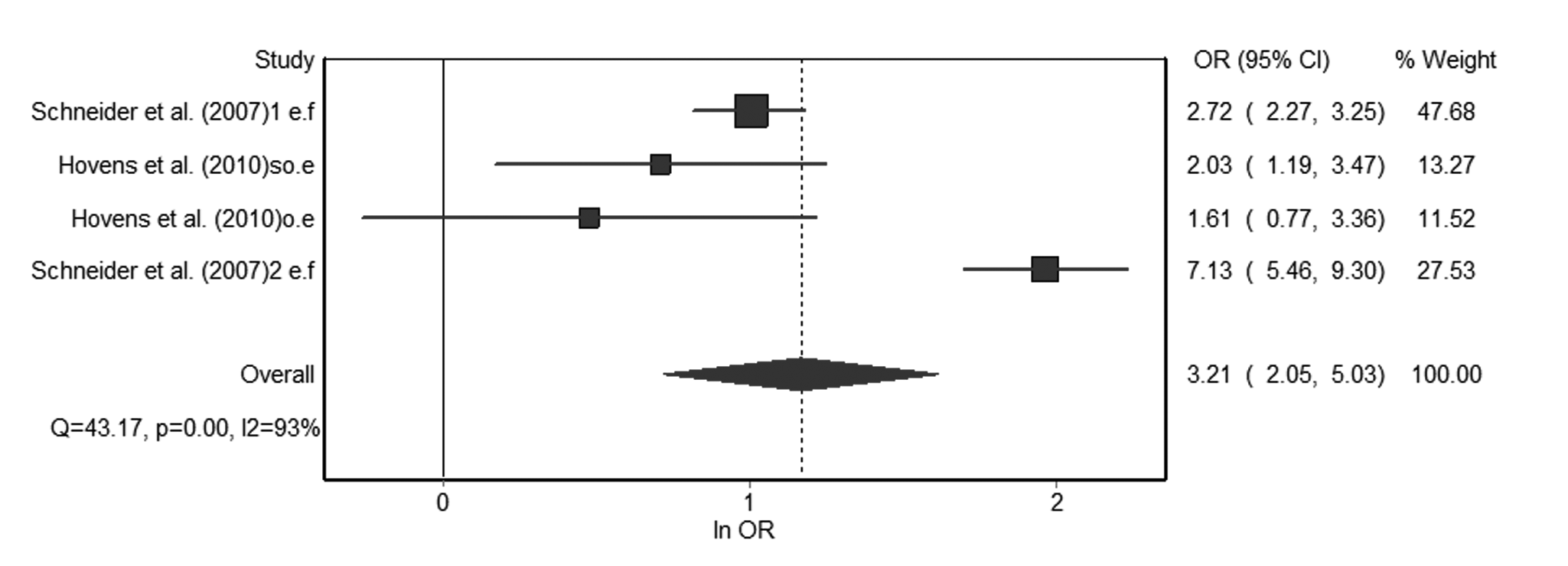

Supplement: Figure S6 — Forest plot for quality-effect meta-analysis of the association between emotional abuse and anxiety. Studies are represented by symbols, the area of which is proportional to the study's weight in the analysis. Output for ORs is set to the (natural) log scale. (TIF) [file pmed.1001349.s006.tif]

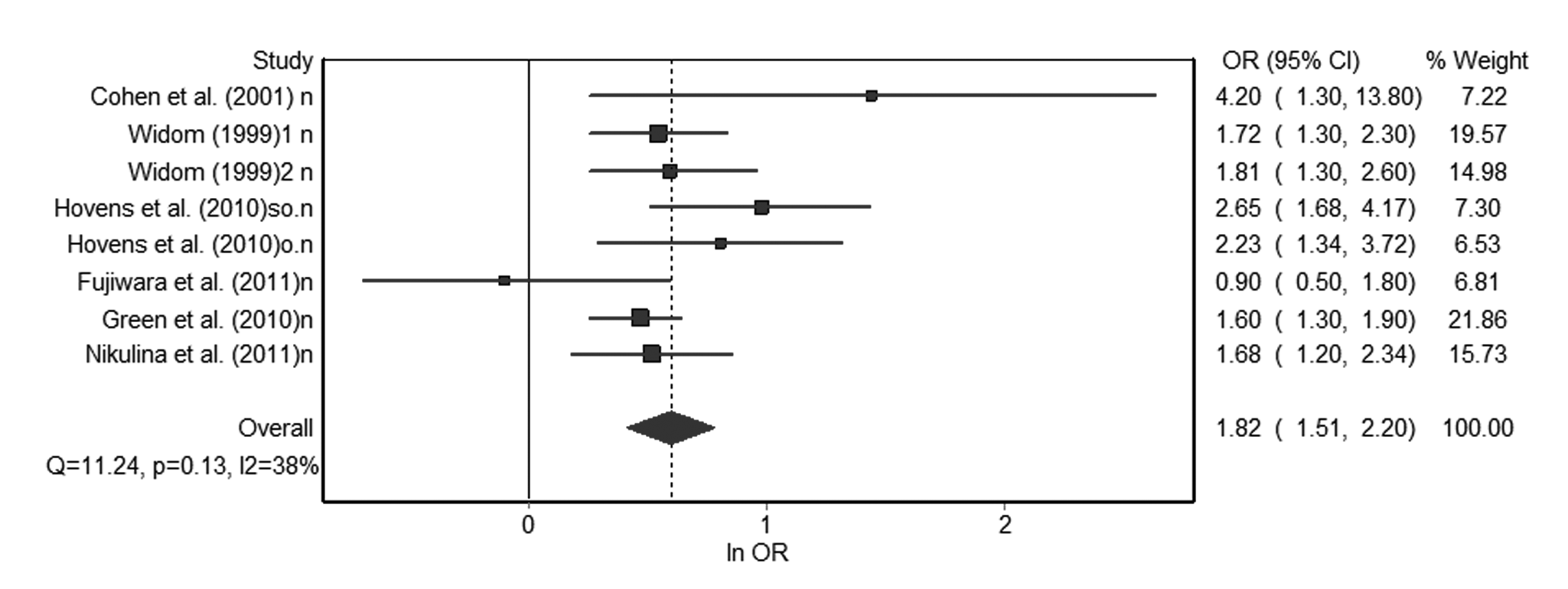

Supplement: Figure S7 — Forest plot for quality-effect meta-analysis of the association between neglect and anxiety. Studies are represented by symbols, the area of which is proportional to the study's weight in the analysis. Output for ORs is set to the (natural) log scale. (TIF) [file pmed.1001349.s007.tif]

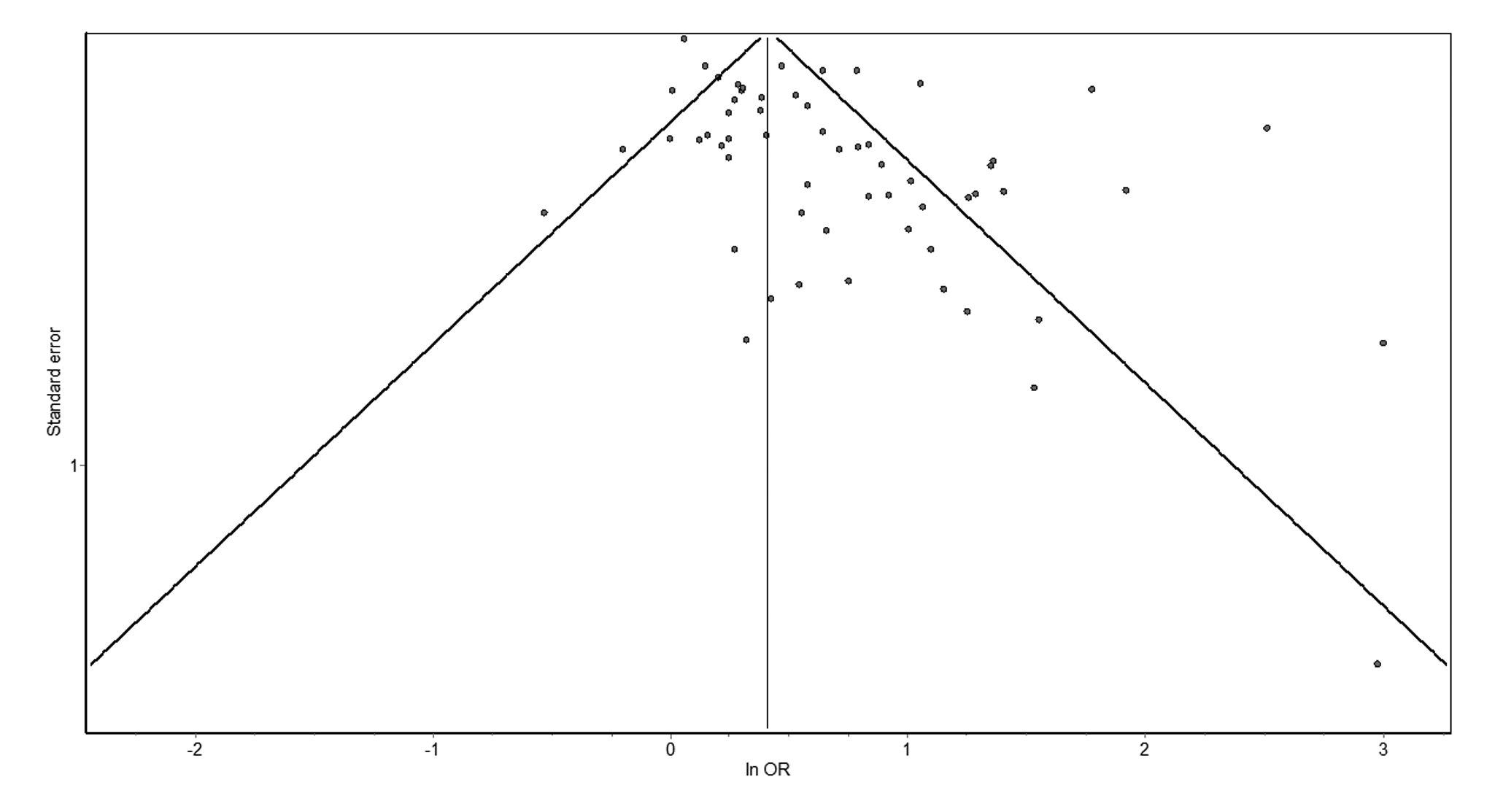

Supplement: Figure S8 — Funnel plot to aid assessment of publication bias for anxiety and physical abuse. (TIF) [file pmed.1001349.s008.tif]

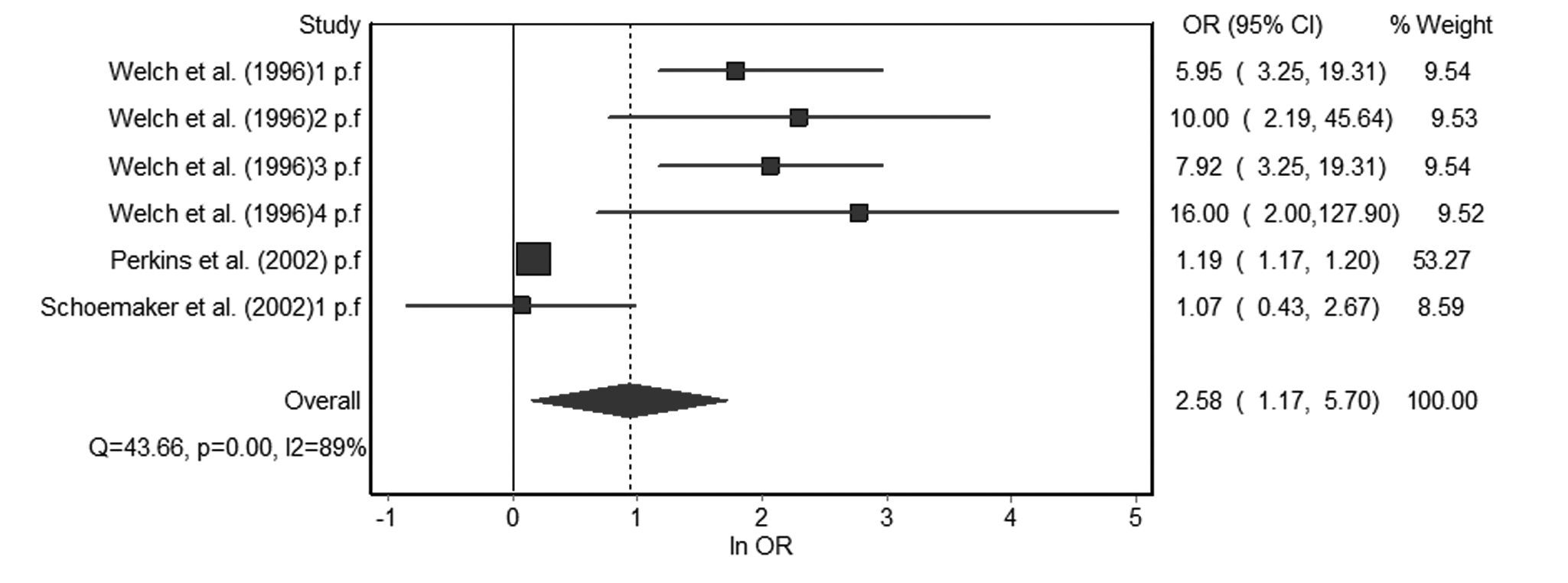

Supplement: Figure S9 — Forest plot for quality-effect meta-analysis of the association between physical abuse and eating disorders. Studies are represented by symbols, the area of which is proportional to the study's weight in the analysis. Output for ORs is set to the (natural) log scale. (TIF) [file pmed.1001349.s009.tif]

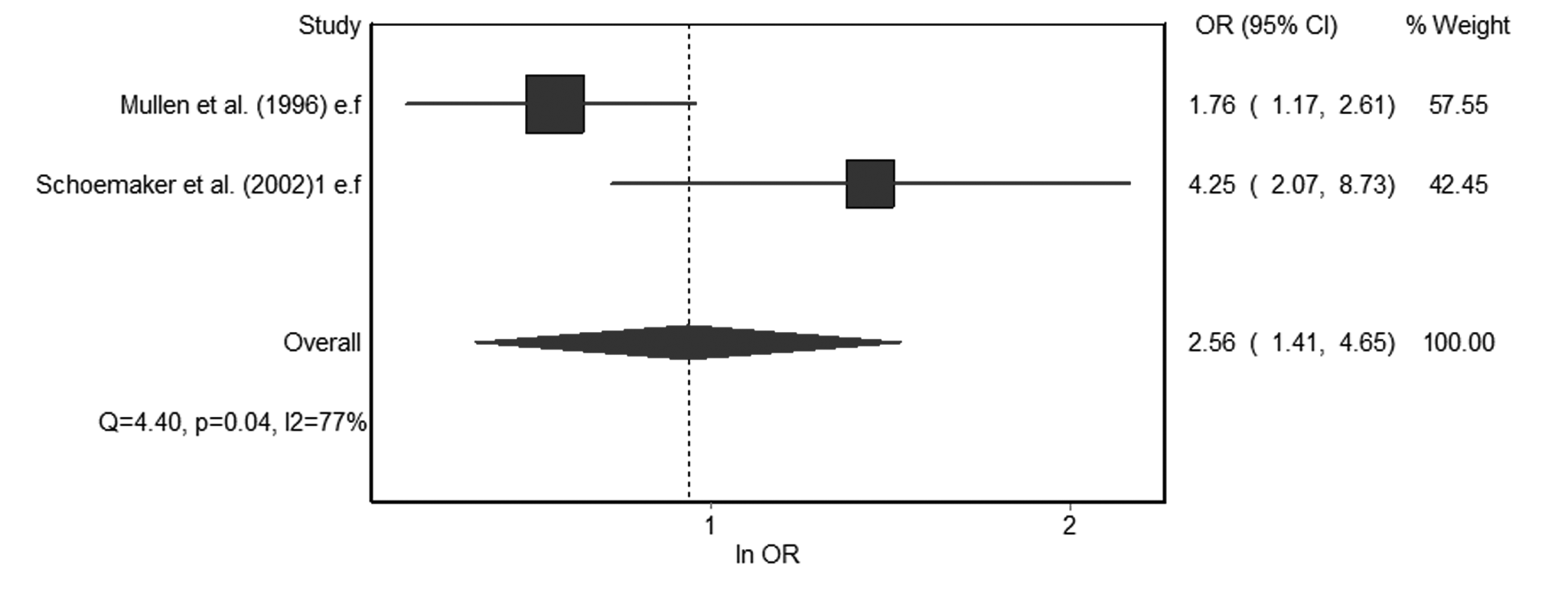

Supplement: Figure S10 — Forest plot for quality-effect meta-analysis of the association between emotional abuse and eating disorders. Studies are represented by symbols, the area of which is proportional to the study's weight in the analysis. Output for ORs is set to the (natural) log scale. (TIF) [file pmed.1001349.s010.tif]

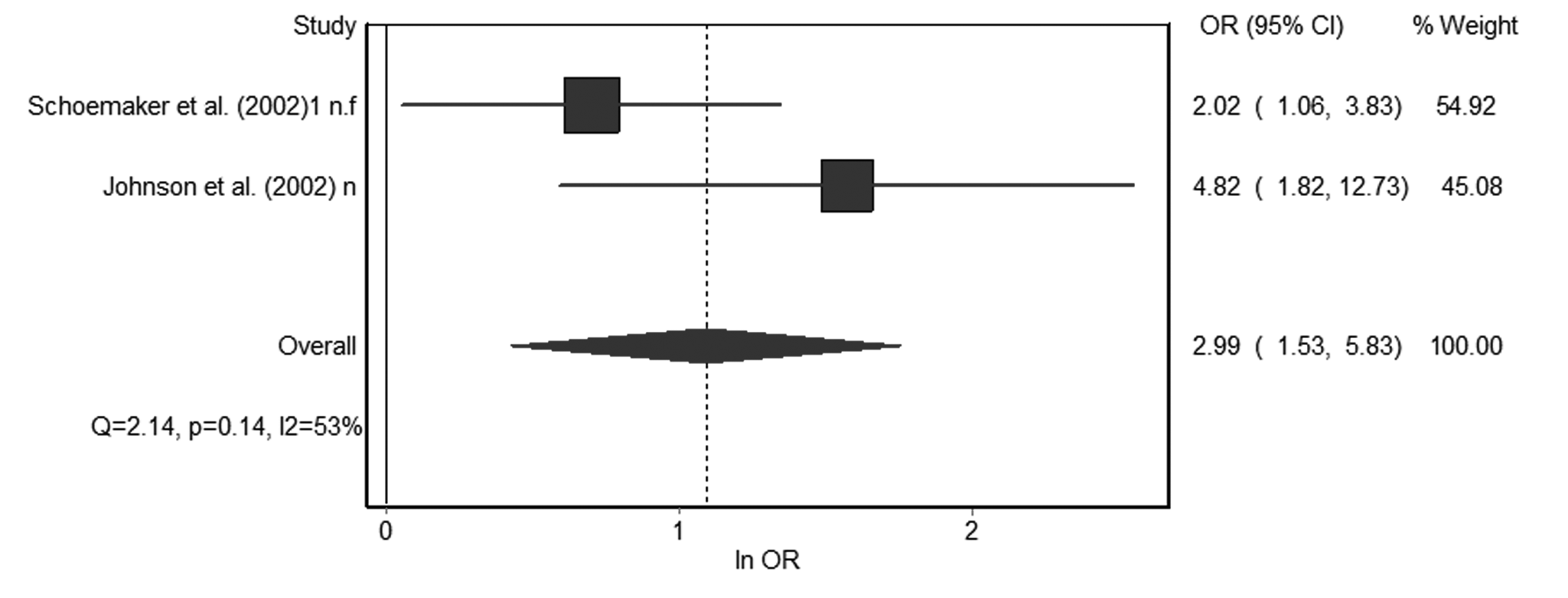

Supplement: Figure S11 — Forest plot for quality-effect meta-analysis of the association between neglect and eating disorders. Studies are represented by symbols, the area of which is proportional to the study's weight in the analysis. Output for ORs is set to the (natural) log scale. (TIF) [file pmed.1001349.s011.tif]

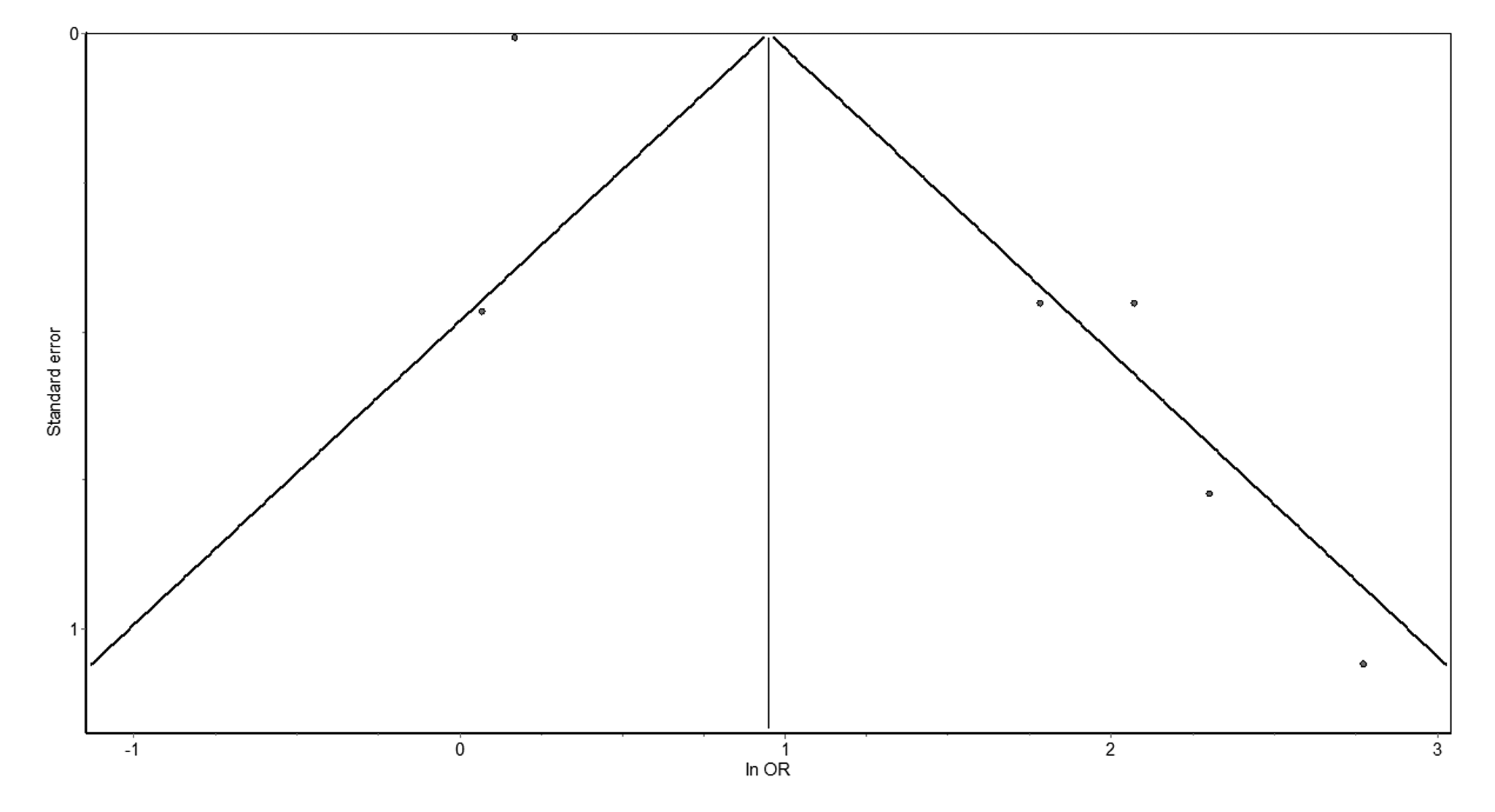

Supplement: Figure S12 — Funnel plot to aid assessment of publication bias for eating disorders and physical abuse. (TIF) [file pmed.1001349.s012.tif]

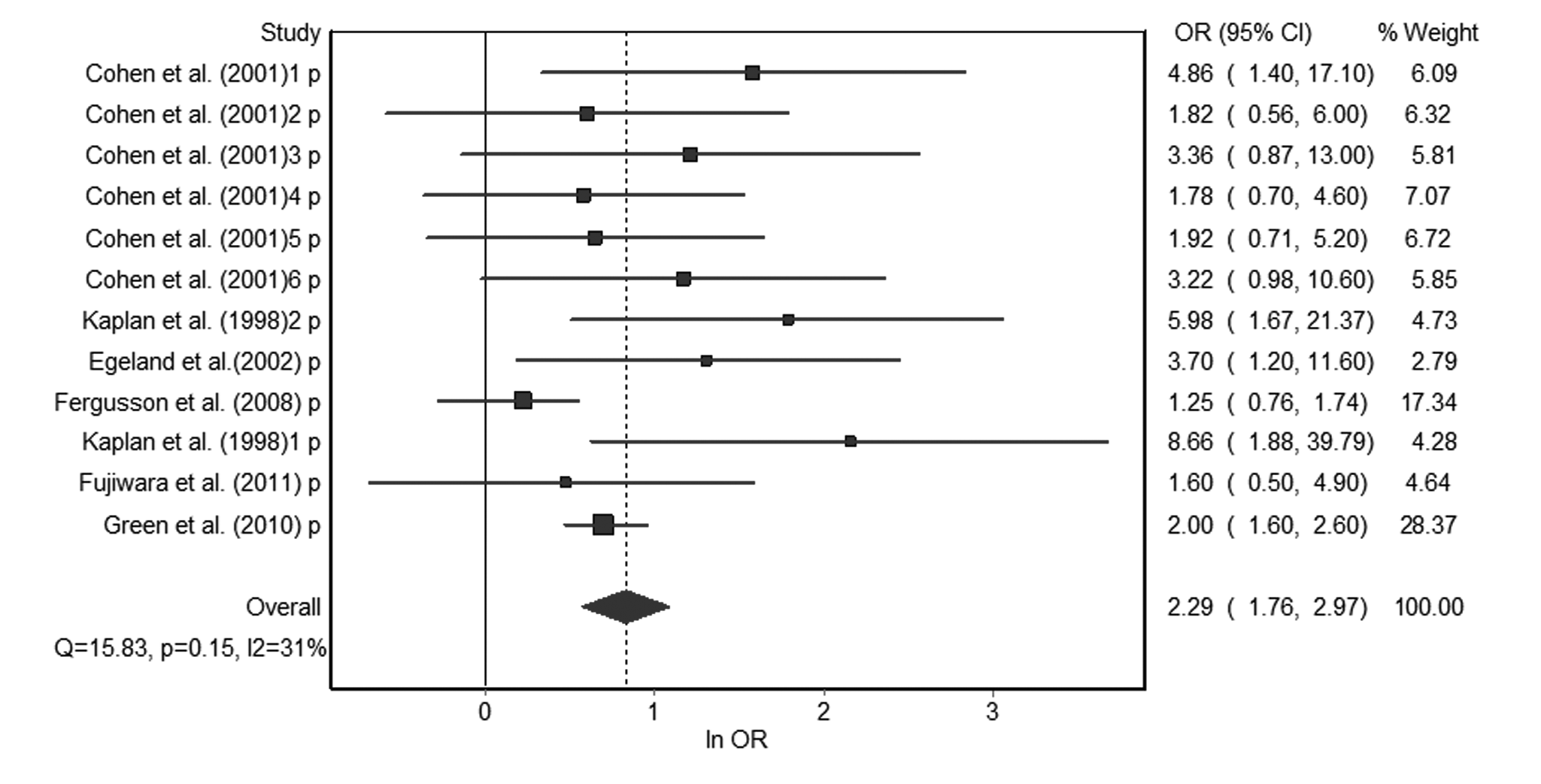

Supplement: Figure S13 — Forest plot for quality-effect meta-analysis of the association between physical abuse and conduct/childhood behavioural disorders. Studies are represented by symbols, the area of which is proportional to the study's weight in the analysis. Output for ORs is set to the (natural) log scale. (TIF) [file pmed.1001349.s013.tif]

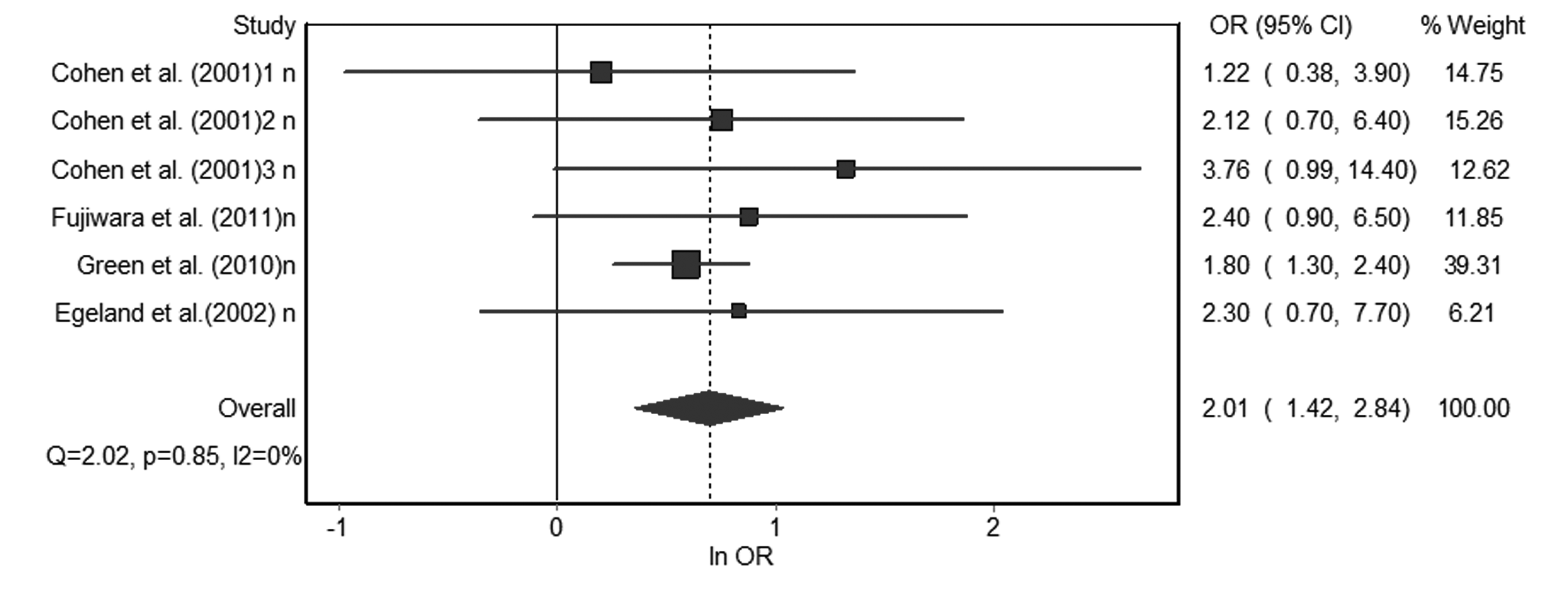

Supplement: Figure S14 — Forest plot for quality-effect meta-analysis of the association between neglect and conduct/childhood behavioural disorders. Studies are represented by symbols, the area of which is proportional to the study's weight in the analysis. Output for ORs is set to the (natural) log scale. (TIF) [file pmed.1001349.s014.tif]

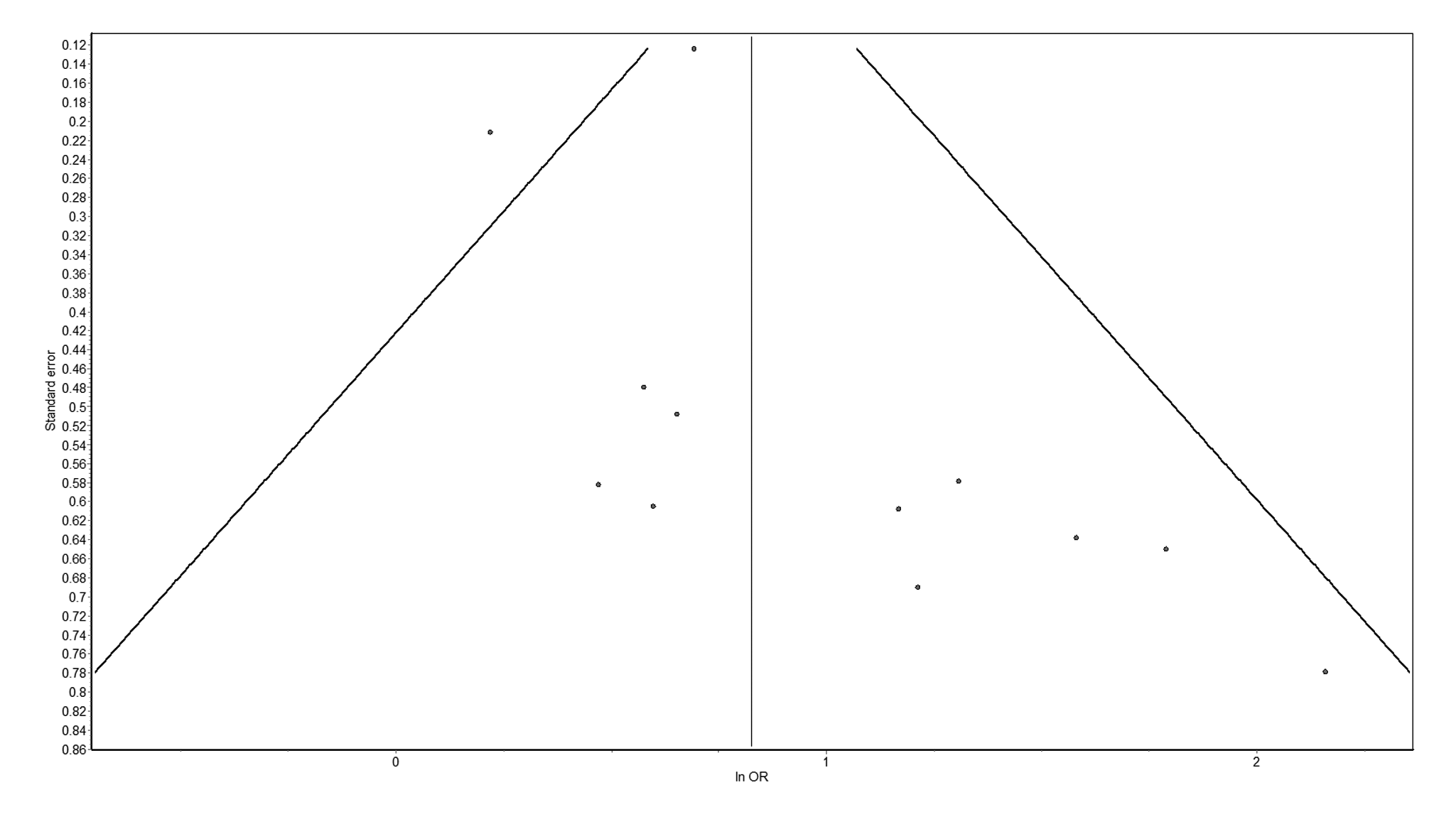

Supplement: Figure S15 — Funnel plot to aid assessment of publication bias for childhood behavioural/conduct disorders and physical abuse. (TIF) [file pmed.1001349.s015.tif]

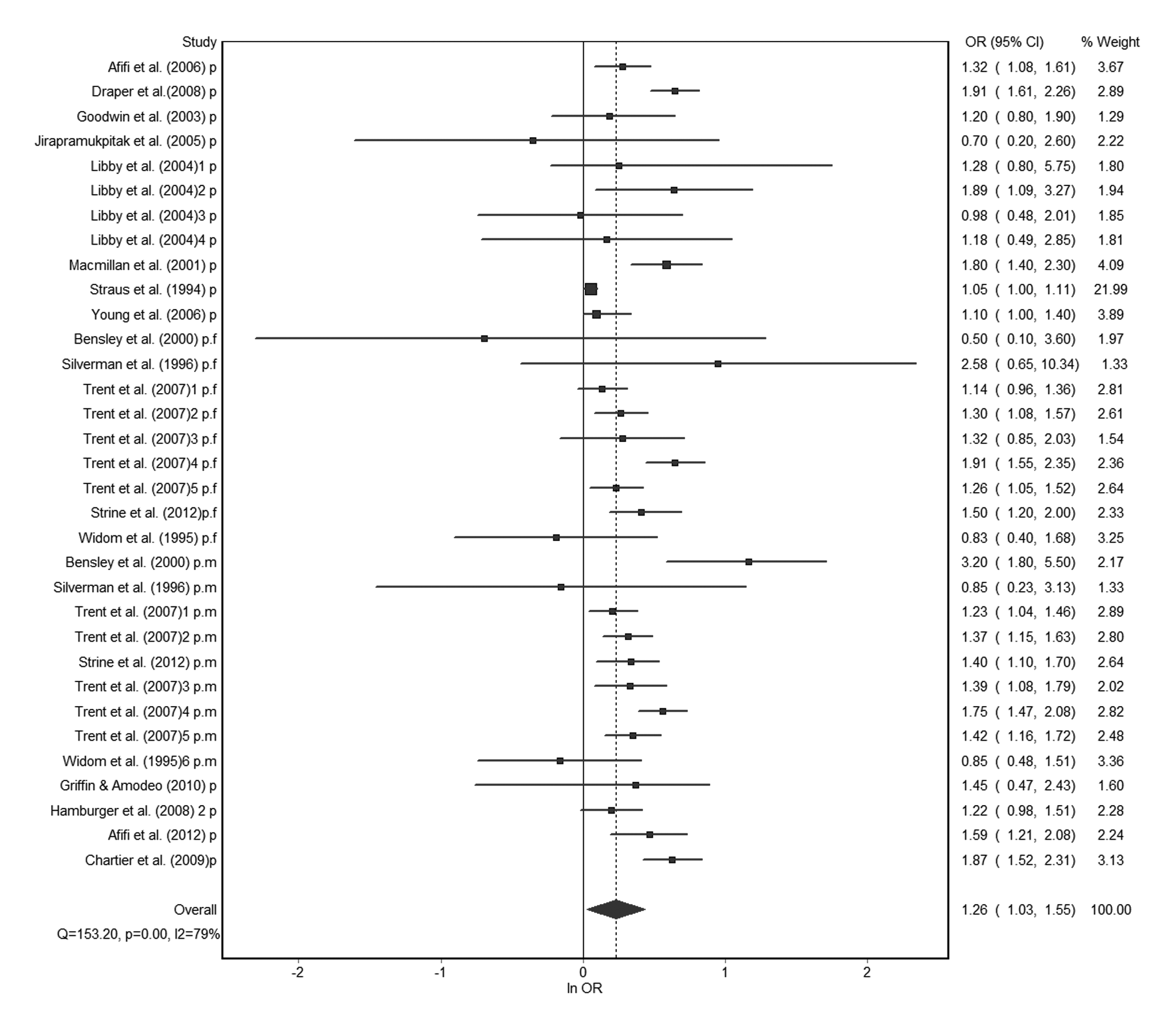

Supplement: Figure S16 — Forest plot for quality-effect meta-analysis of the association between physical abuse and alcohol problem drinking. Studies are represented by symbols, the area of which is proportional to the study's weight in the analysis. Output for ORs is set to the (natural) log scale. (TIF) [file pmed.1001349.s016.tif]

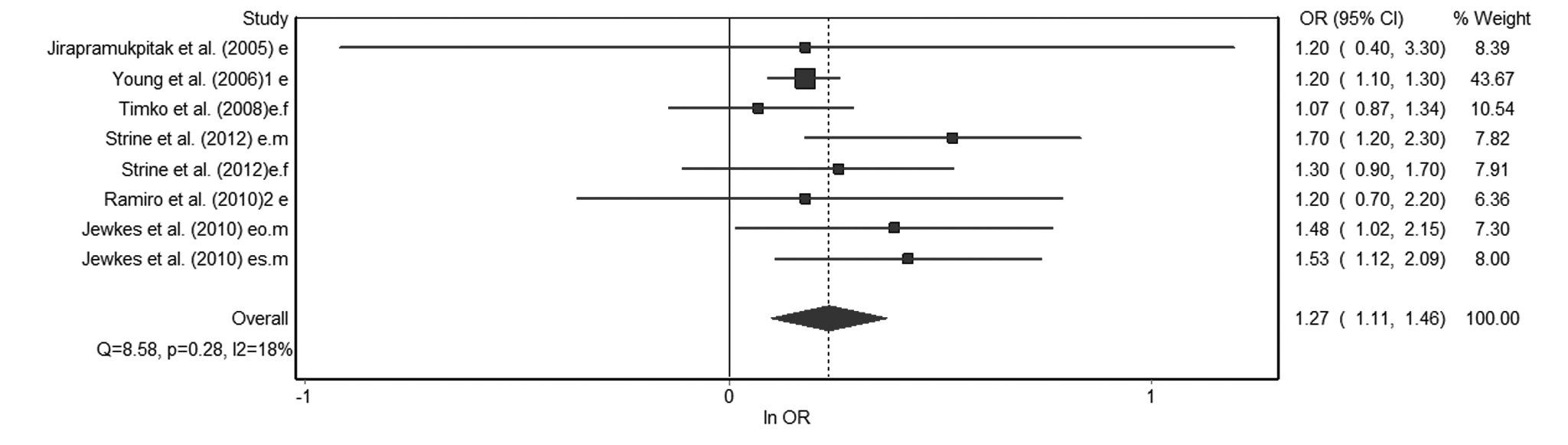

Supplement: Figure S17 — Forest plot for quality-effect meta-analysis of the association between emotional abuse and alcohol problem drinking. Studies are represented by symbols, the area of which is proportional to the study's weight in the analysis. Output for ORs is set to the (natural) log scale. (TIF) [file pmed.1001349.s017.tif]

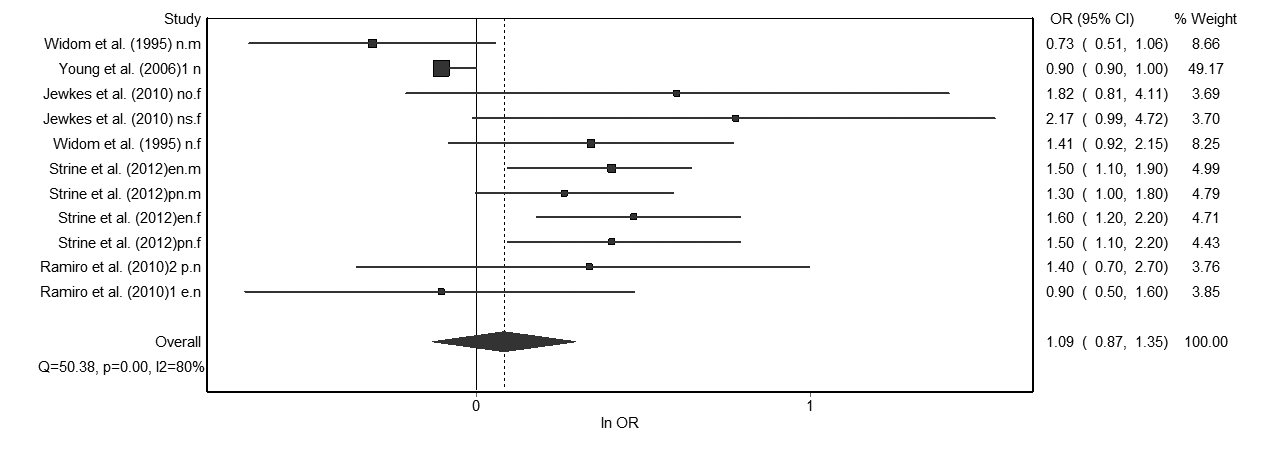

Supplement: Figure S18 — Forest plot for quality-effect meta-analysis of the association between neglect and alcohol problem drinking. Studies are represented by symbols, the area of which is proportional to the study's weight in the analysis. Output for ORs is set to the (natural) log scale. (TIF) [file pmed.1001349.s018.tif]

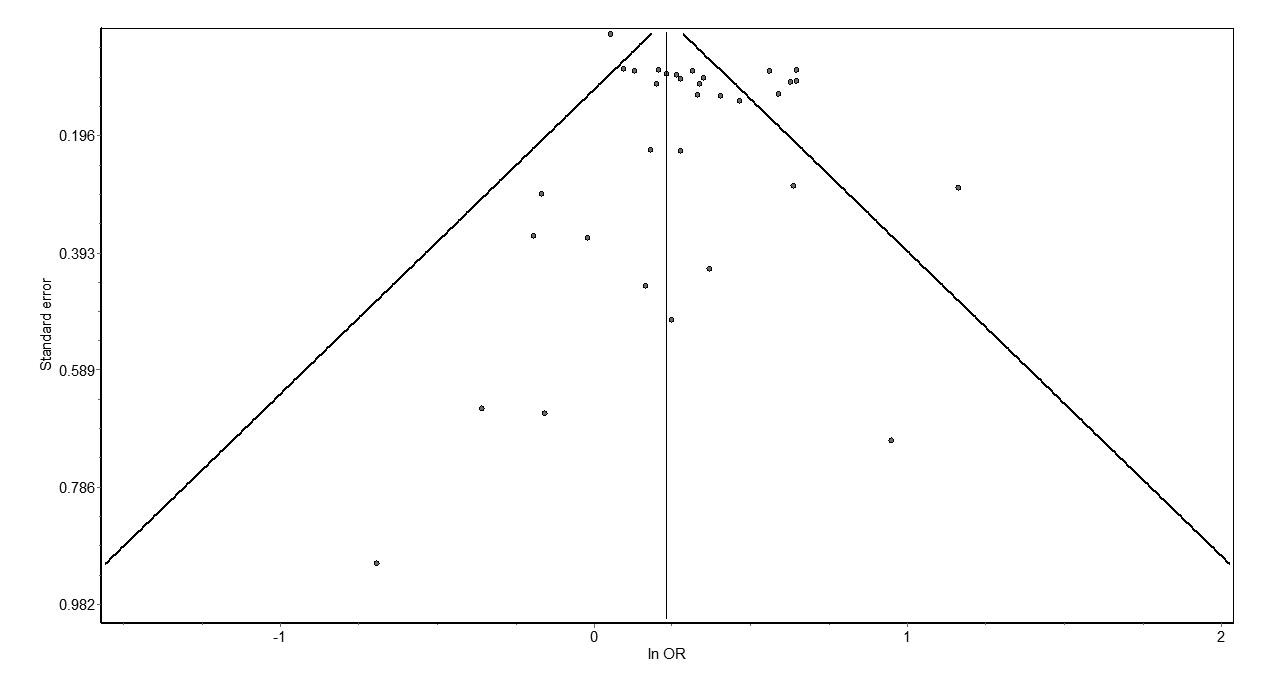

Supplement: Figure S19 — Funnel plot to aid assessment of publication bias for alcohol problem drinking and physical abuse. (TIF) [file pmed.1001349.s019.tif]

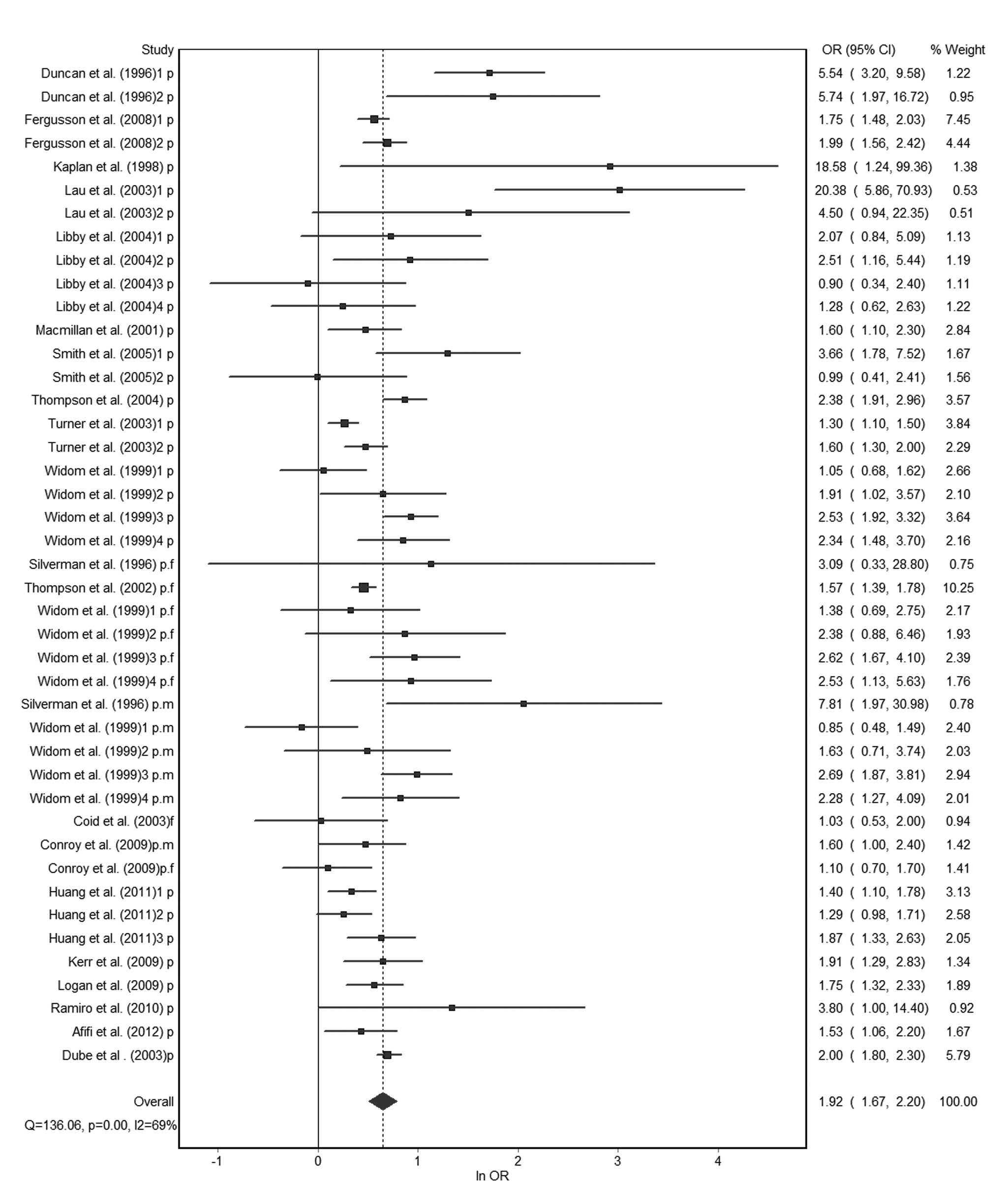

Supplement: Figure S20 — Forest plot for quality-effect meta-analysis of the association between physical abuse and drug use. Studies are represented by symbols, the area of which is proportional to the study's weight in the analysis. Output for ORs is set to the (natural) log scale. (TIF) [file pmed.1001349.s020.tif]

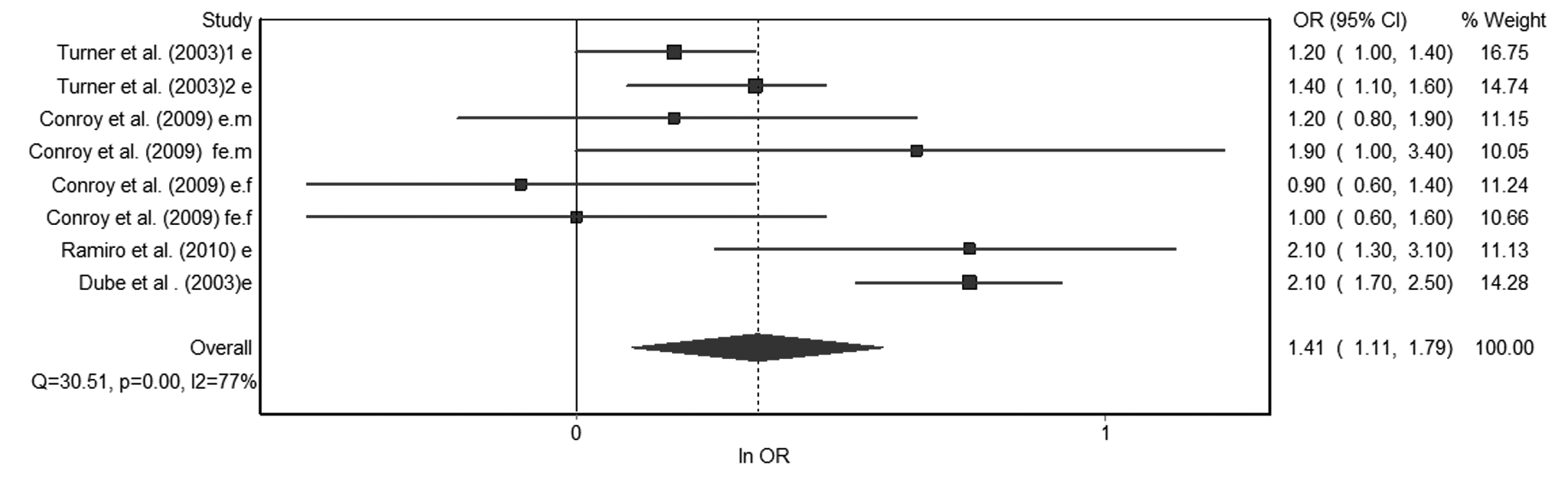

Supplement: Figure S21 — Forest plot for quality-effect meta-analysis of the association between emotional abuse and drug use. Studies are represented by symbols, the area of which is proportional to the study's weight in the analysis. Output for ORs is set to the (natural) log scale. (TIF) [file pmed.1001349.s021.tif]

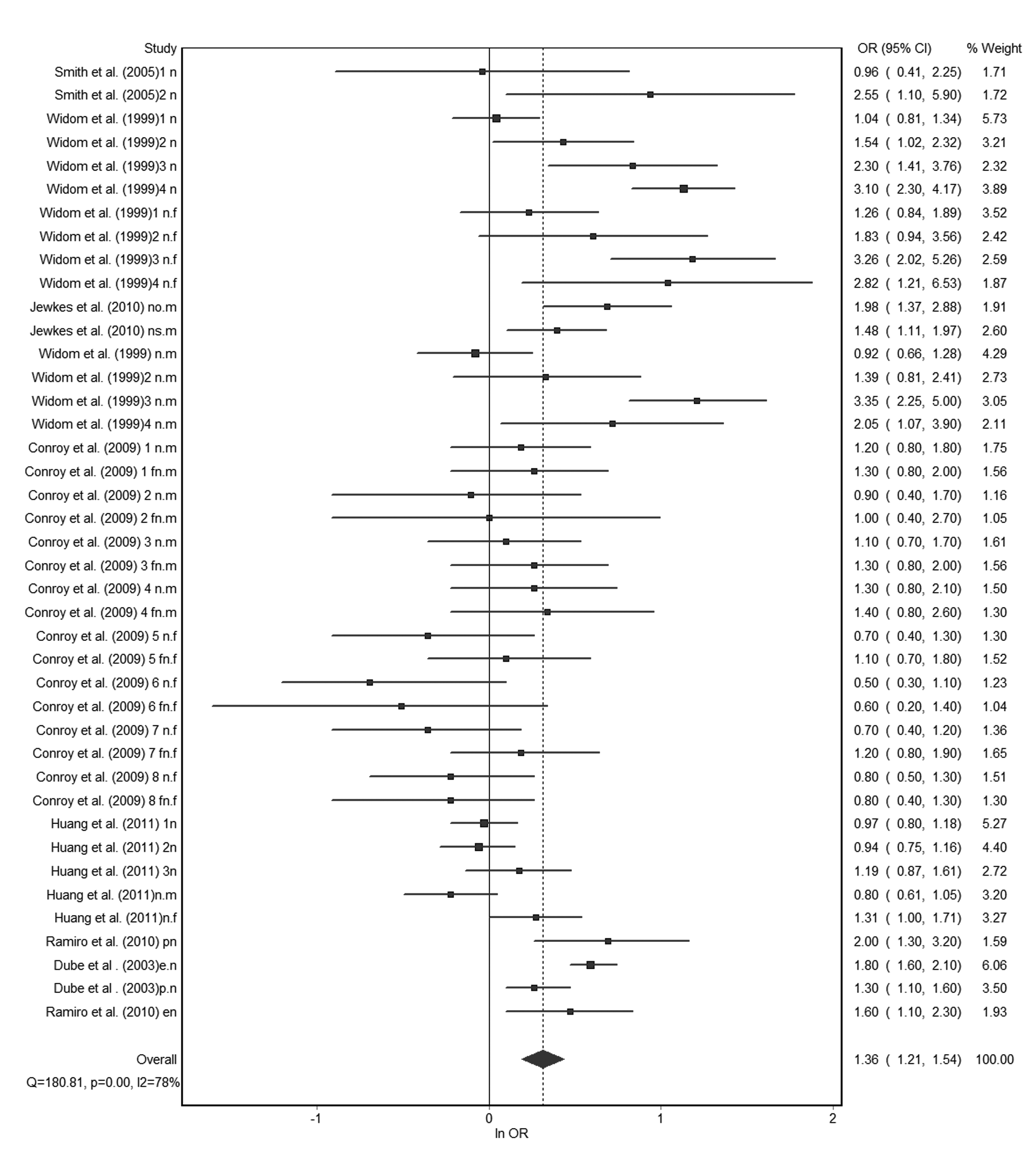

Supplement: Figure S22 — Forest plot for quality-effect meta-analysis of the association between neglect and drug use. Studies are represented by symbols, the area of which is proportional to the study's weight in the analysis. Output for ORs is set to the (natural) log scale. (TIF) [file pmed.1001349.s022.tif]

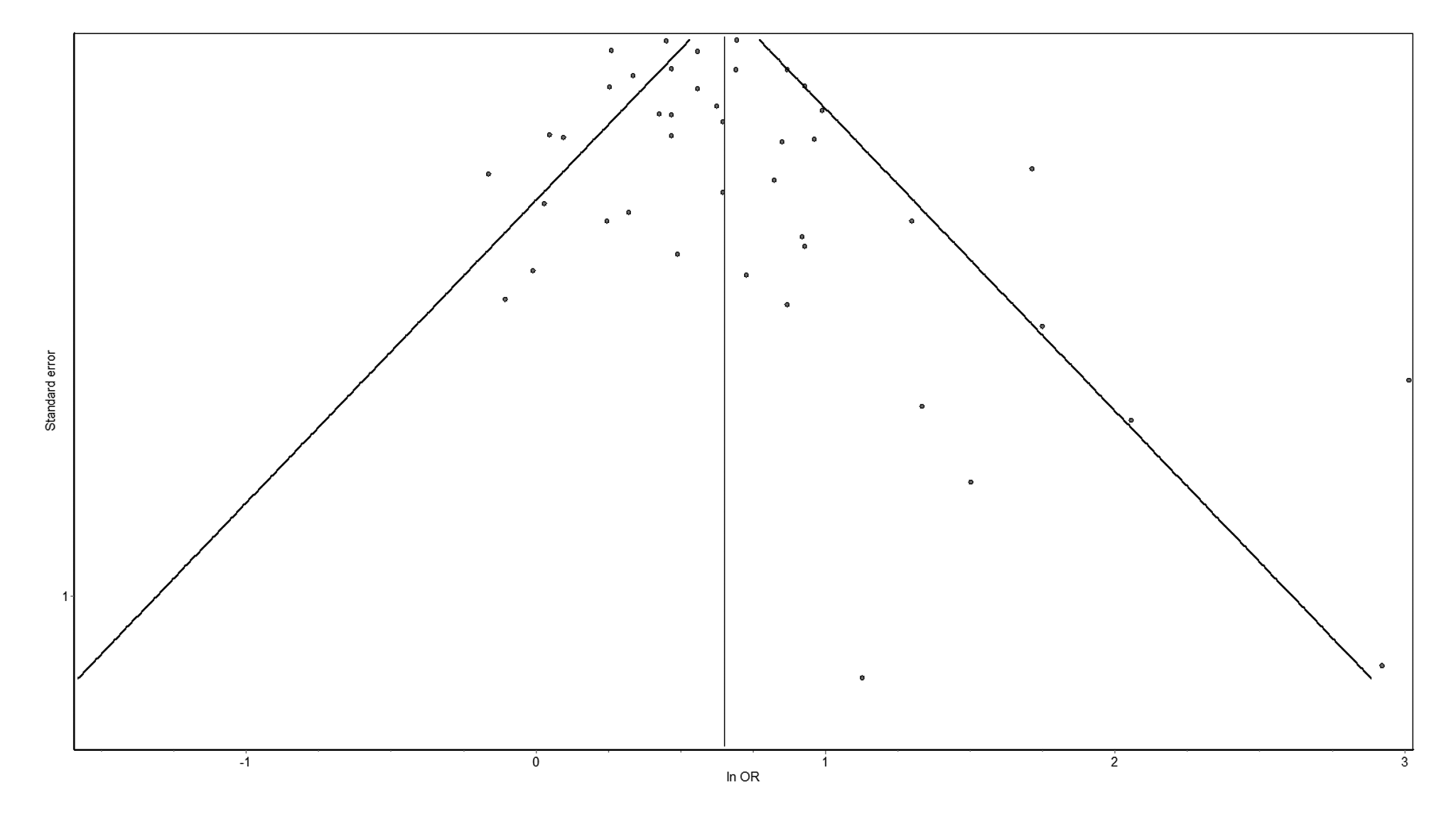

Supplement: Figure S23 — Funnel plot to aid assessment of publication bias for drug use and physical abuse. (TIF) [file pmed.1001349.s023.tif]

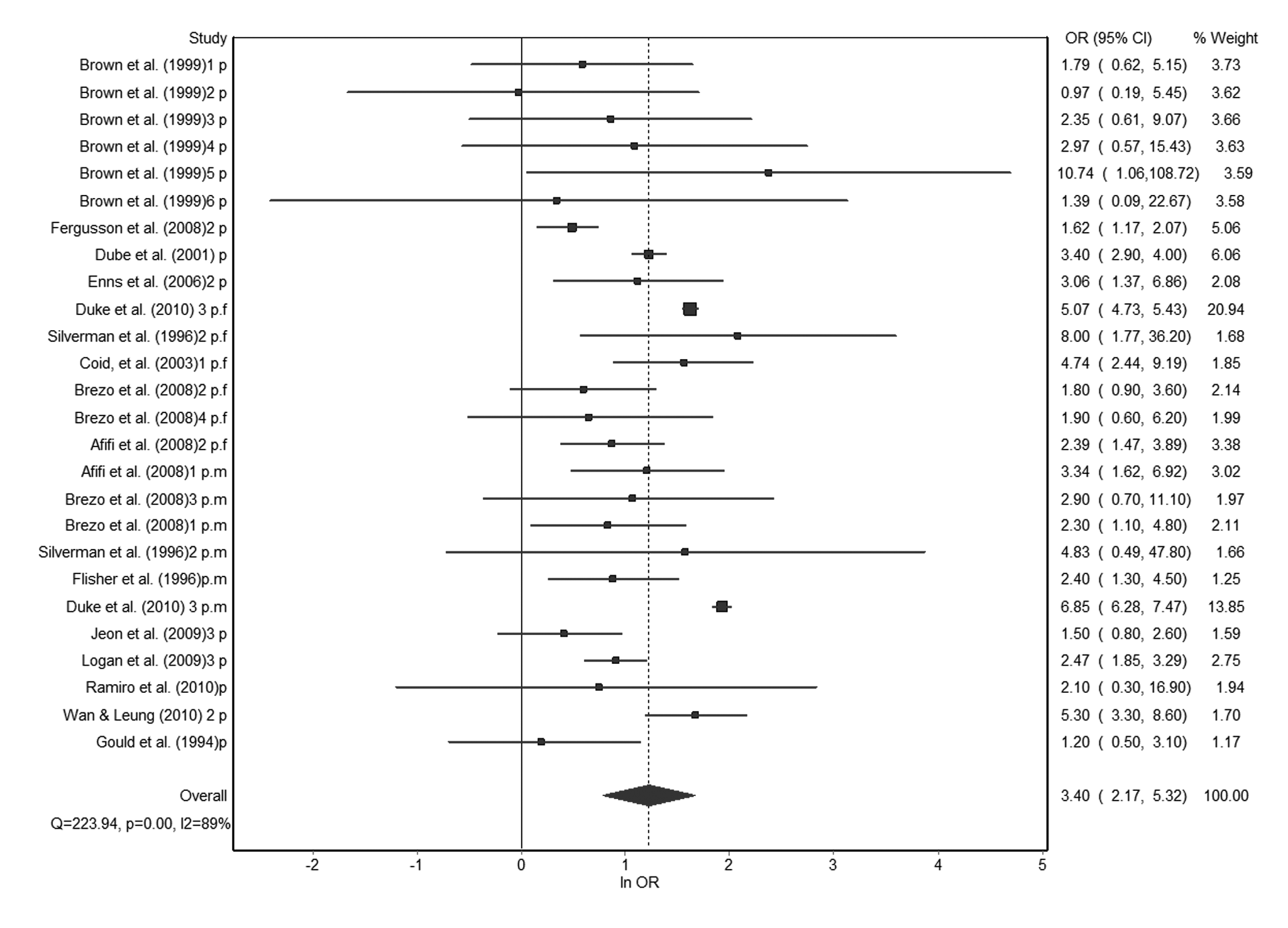

Supplement: Figure S24 — Forest plot for quality-effect meta-analysis of the association between physical abuse and suicide attempt. Studies are represented by symbols, the area of which is proportional to the study's weight in the analysis. Output for ORs is set to the (natural) log scale. (TIF) [file pmed.1001349.s024.tif]

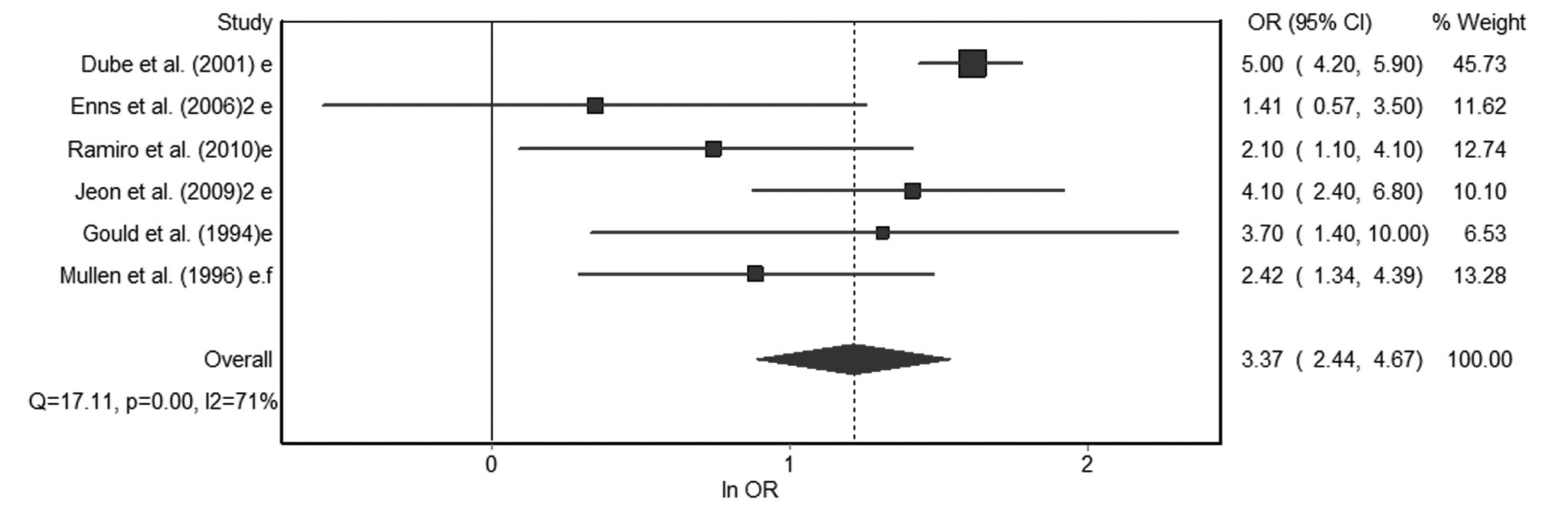

Supplement: Figure S25 — Forest plot for quality-effect meta-analysis of the association between emotional abuse and suicide attempt. Studies are represented by symbols, the area of which is proportional to the study's weight in the analysis. Output for ORs is set to the (natural) log scale. (TIF) [file pmed.1001349.s025.tif]

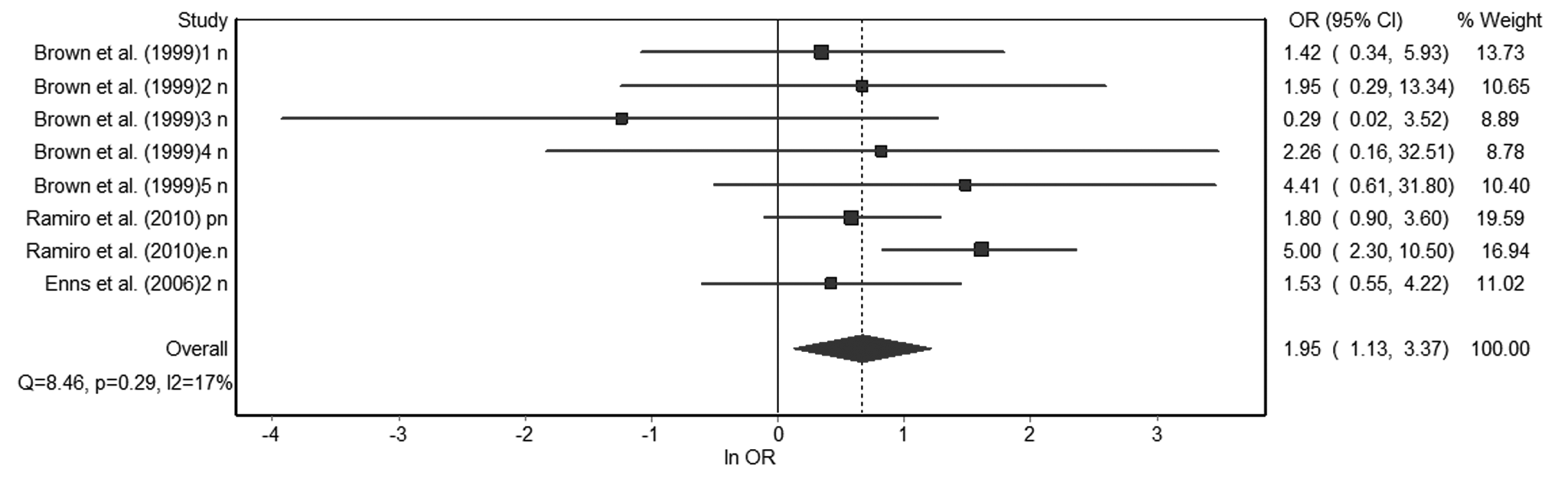

Supplement: Figure S26 — Forest plot for quality-effect meta-analysis of the association between neglect and suicide attempt. Studies are represented by symbols, the area of which is proportional to the study's weight in the analysis. Output for ORs is set to the (natural) log scale. (TIF) [file pmed.1001349.s026.tif]

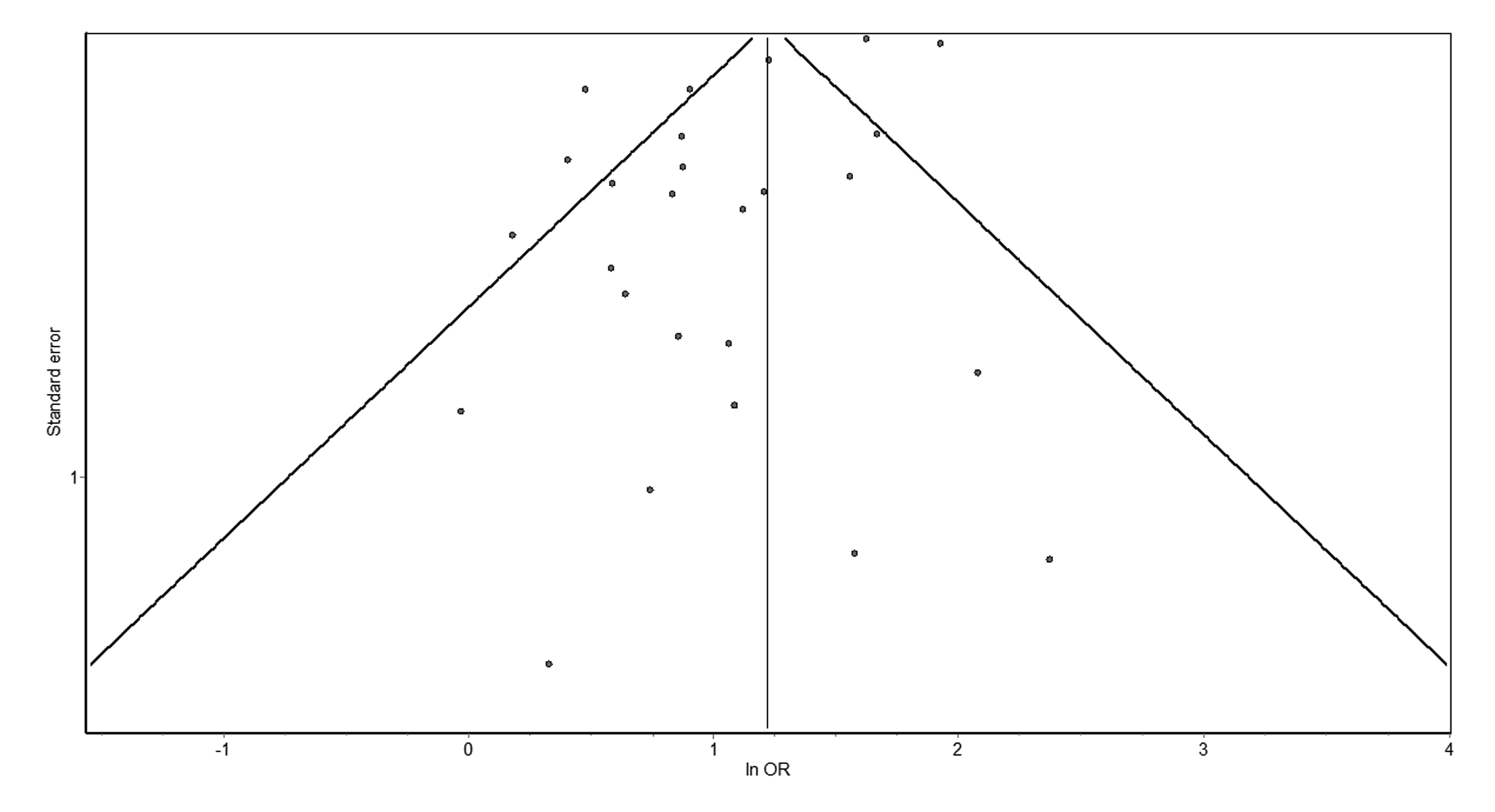

Supplement: Figure S27 — Funnel plot to aid assessment of publication bias for suicide attempt and physical abuse. (TIF) [file pmed.1001349.s027.tif]

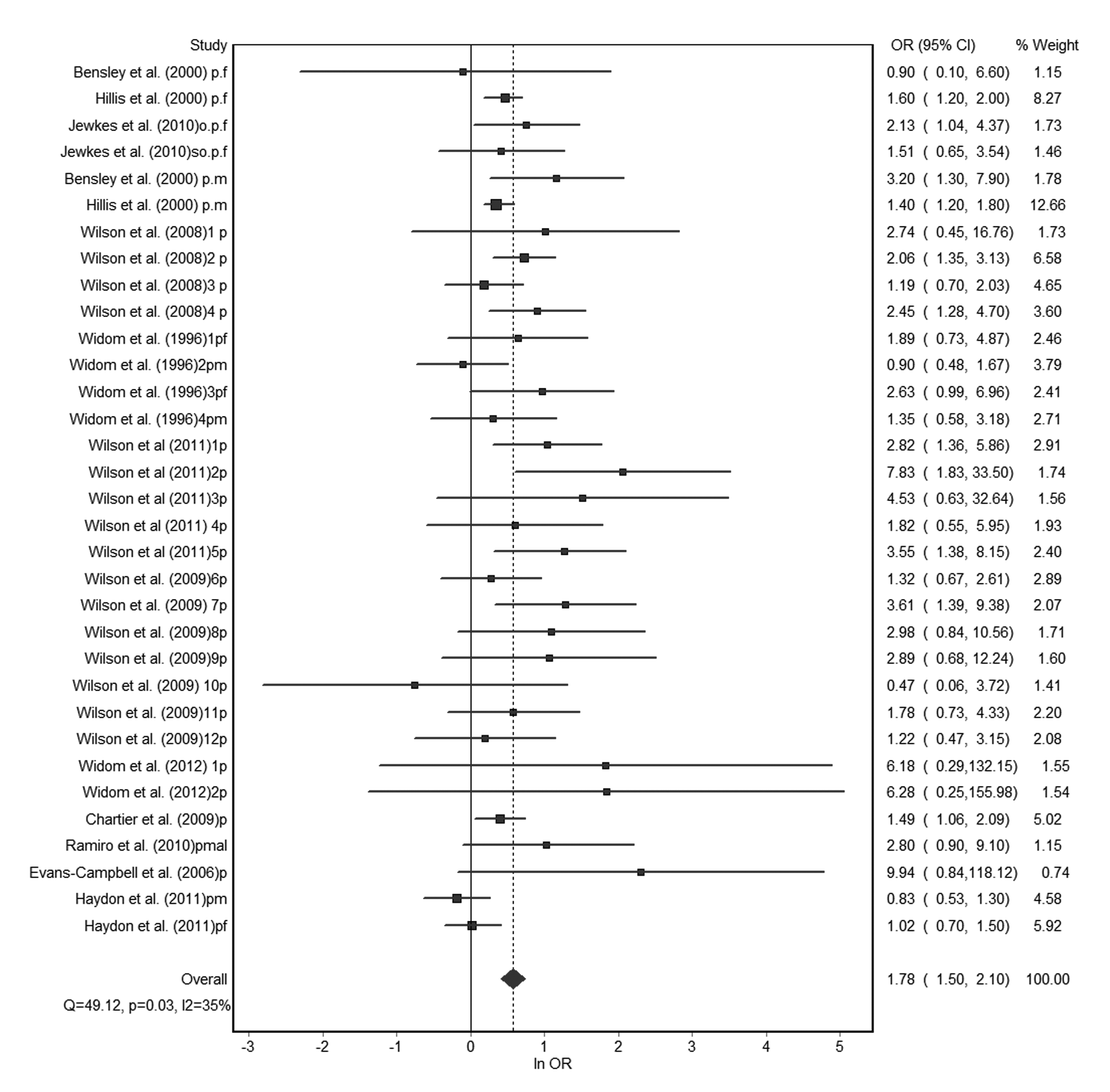

Supplement: Figure S28 — Forest plot for quality-effect meta-analysis of the association between physical abuse and sexually transmitted infections/risky sexual behaviour. Studies are represented by symbols, the area of which is proportional to the study's weight in the analysis. Output for ORs is set to the (natural) log scale. (TIF) [file pmed.1001349.s028.tif]

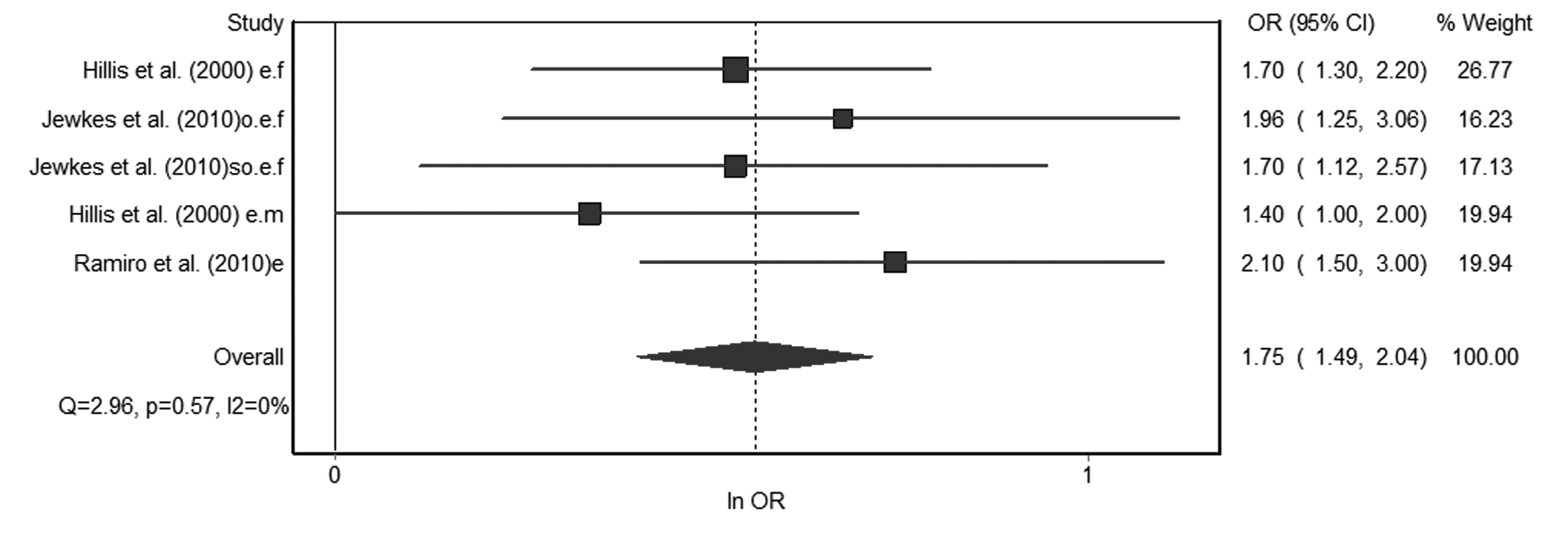

Supplement: Figure S29 — Forest plot for quality-effect meta-analysis of the association between emotional abuse and sexually transmitted infections/risky sexual behaviour. Studies are represented by symbols, the area of which is proportional to the study's weight in the analysis. Output for ORs is set to the (natural) log scale. (TIF) [file pmed.1001349.s029.tif]

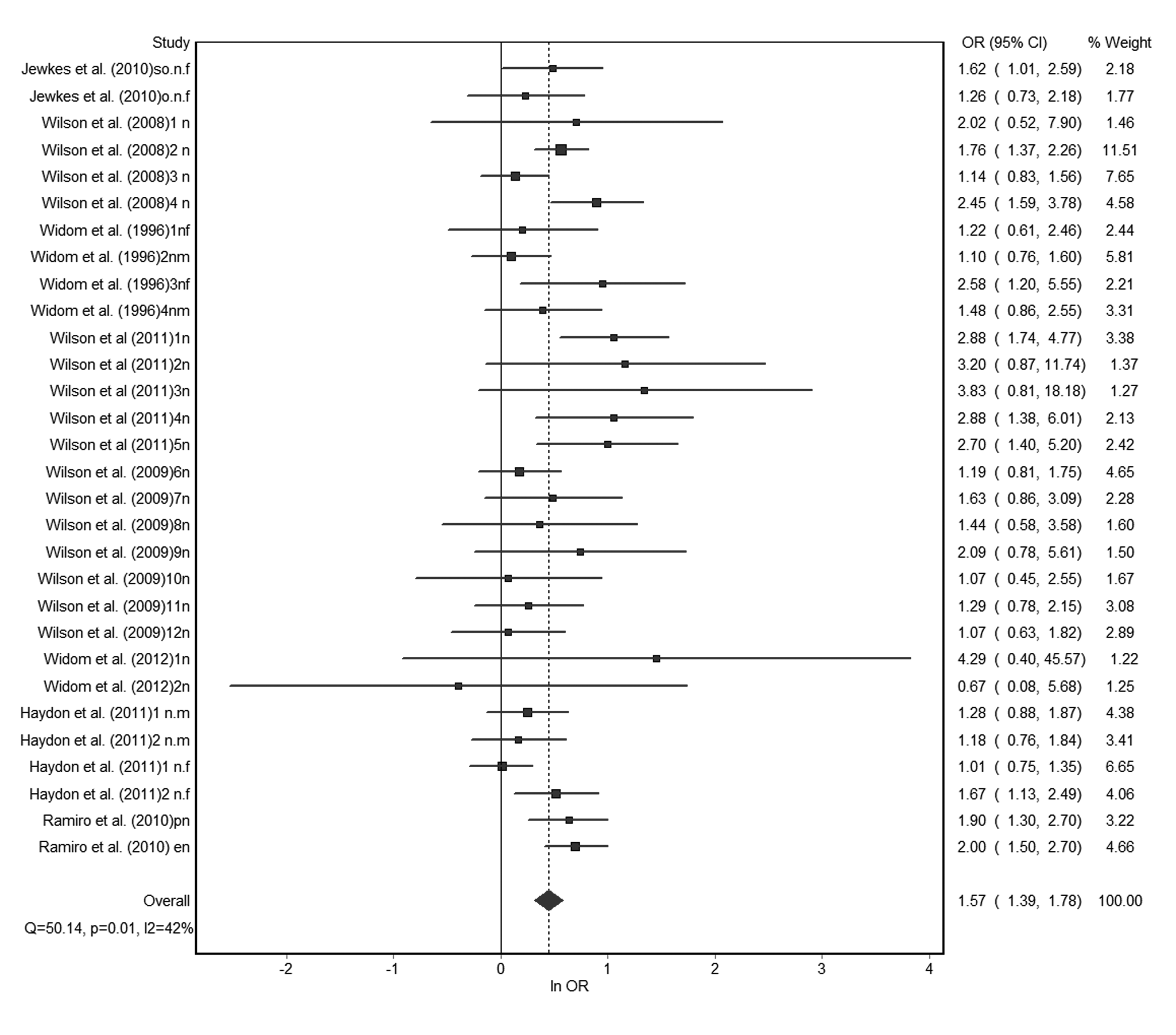

Supplement: Figure S30 — Forest plot for quality-effect meta-analysis of the association between neglect and sexually transmitted infections/risky sexual behaviour. Studies are represented by symbols, the area of which is proportional to the study's weight in the analysis. Output for ORs is set to the (natural) log scale. (TIF) [file pmed.1001349.s030.tif]

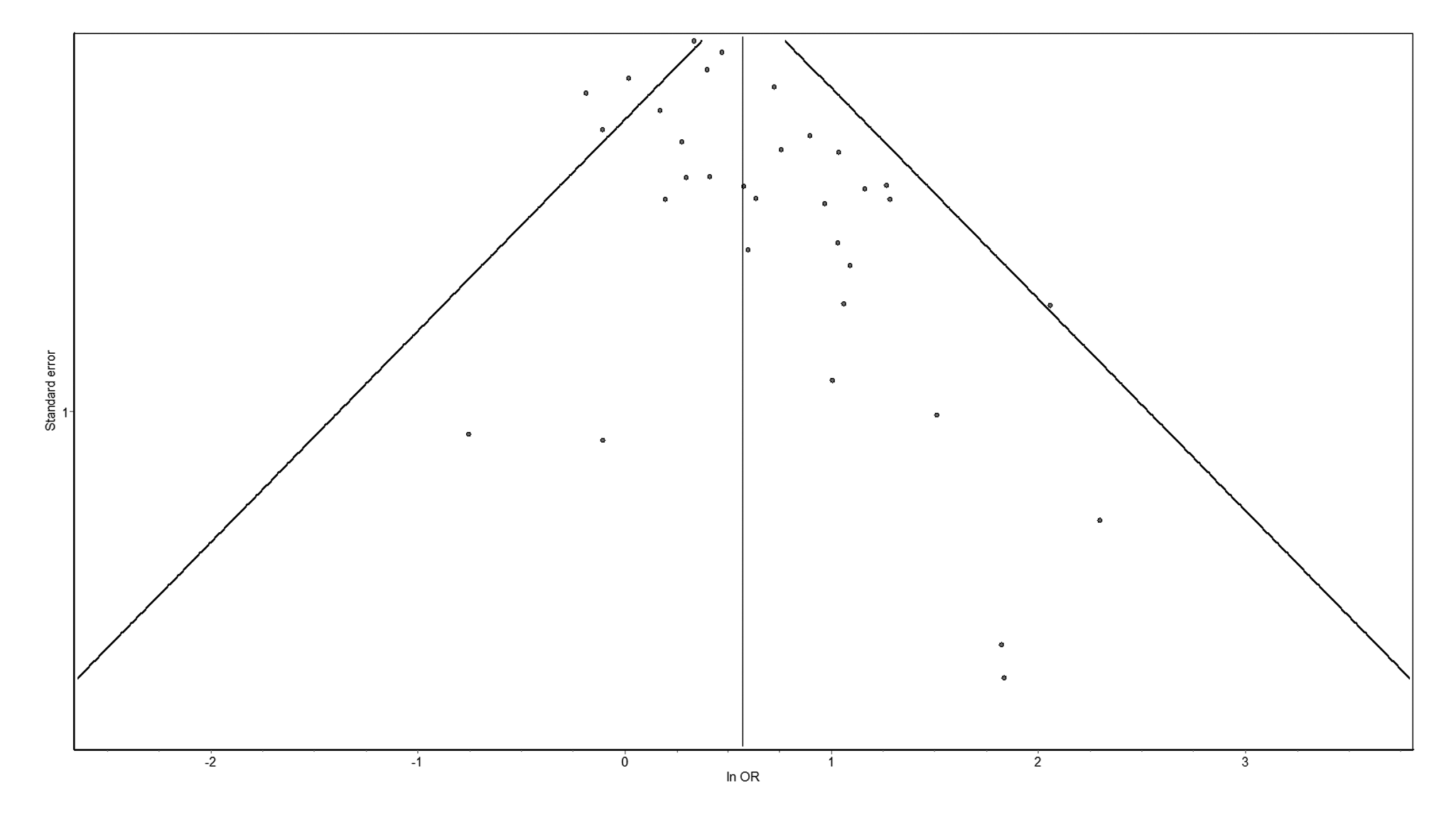

Supplement: Figure S31 — Funnel plot to aid assessment of publication bias for sexually transmitted infections/risky sexual behaviour and physical abuse. (TIF) [file pmed.1001349.s031.tif]

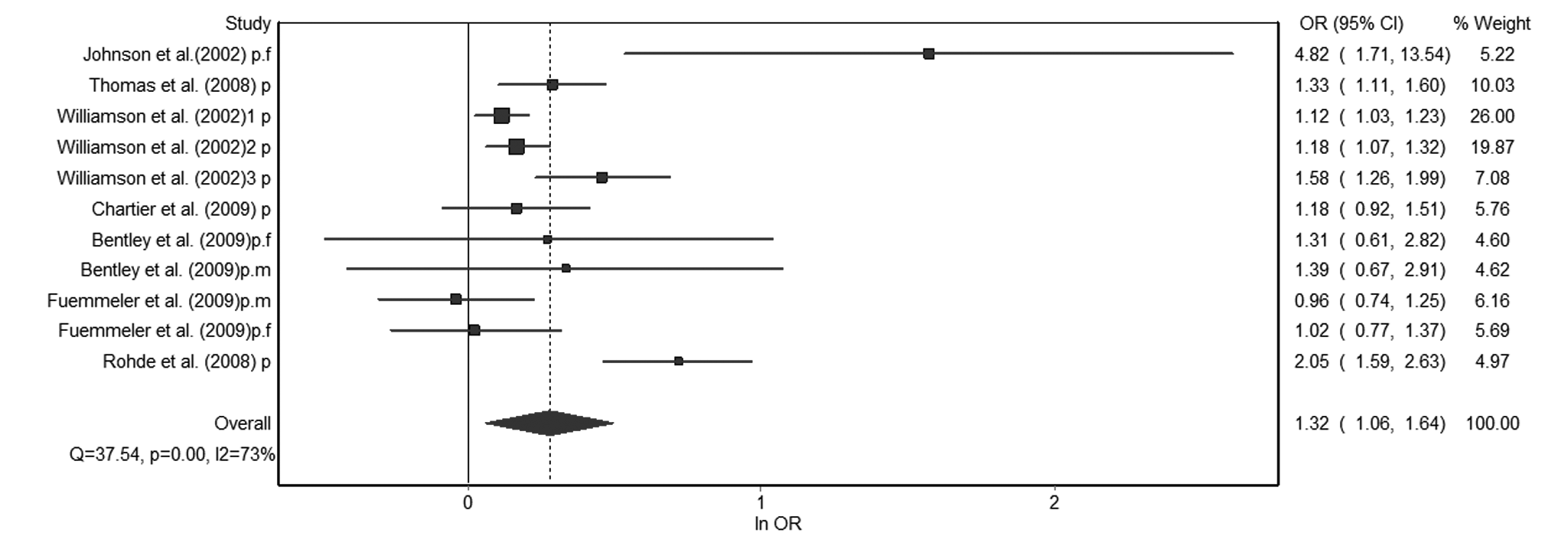

Supplement: Figure S32 — Forest plot for quality-effect meta-analysis of the association between physical abuse and obesity. Studies are represented by symbols, the area of which is proportional to the study's weight in the analysis. Output for ORs is set to the (natural) log scale. (TIF) [file pmed.1001349.s032.tif]

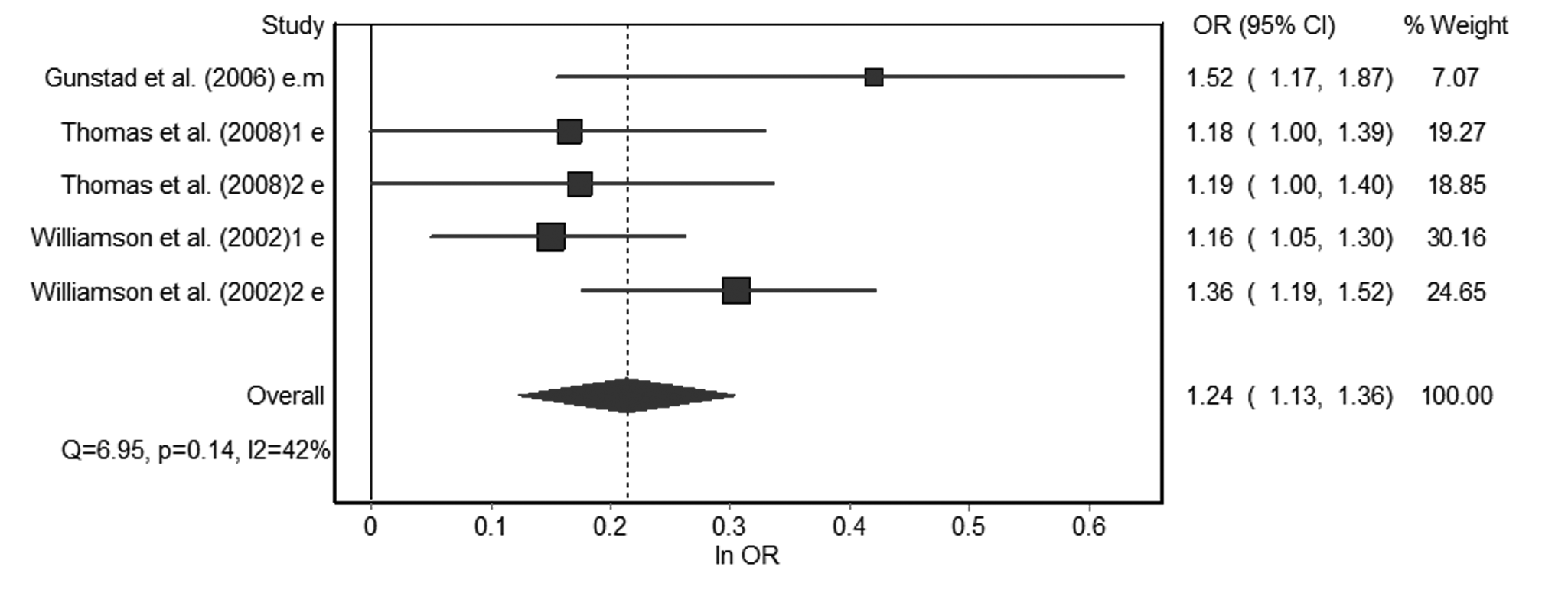

Supplement: Figure S33 — Forest plot for quality-effect meta-analysis of the association between emotional abuse and obesity. Studies are represented by symbols, the area of which is proportional to the study's weight in the analysis. Output for ORs is set to the (natural) log scale. (TIF) [file pmed.1001349.s033.tif]

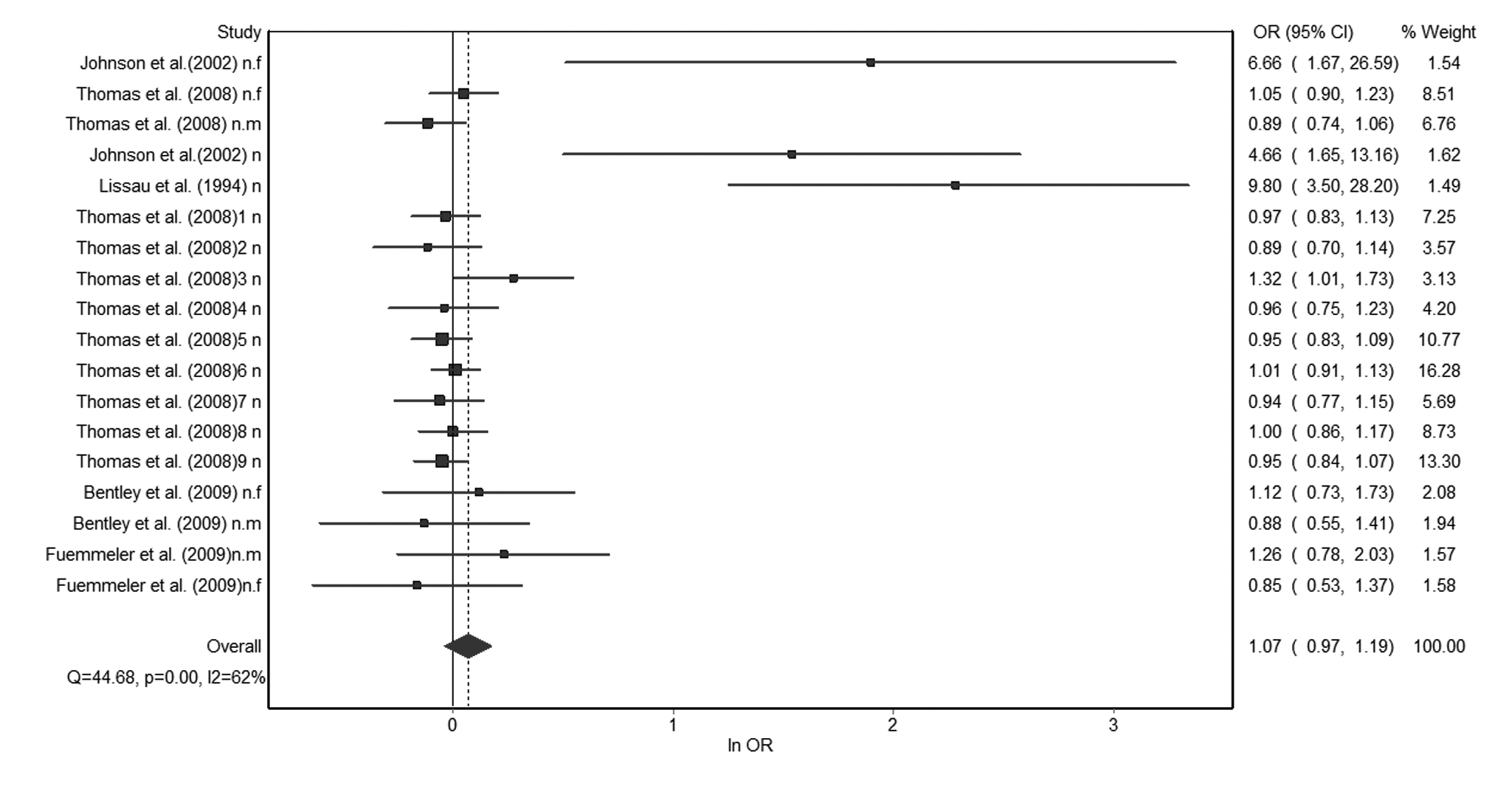

Supplement: Figure S34 — Forest plot for quality-effect meta-analysis of the association between neglect and obesity. Studies are represented by symbols, the area of which is proportional to the study's weight in the analysis. Output for ORs is set to the (natural) log scale. (TIF) [file pmed.1001349.s034.tif]

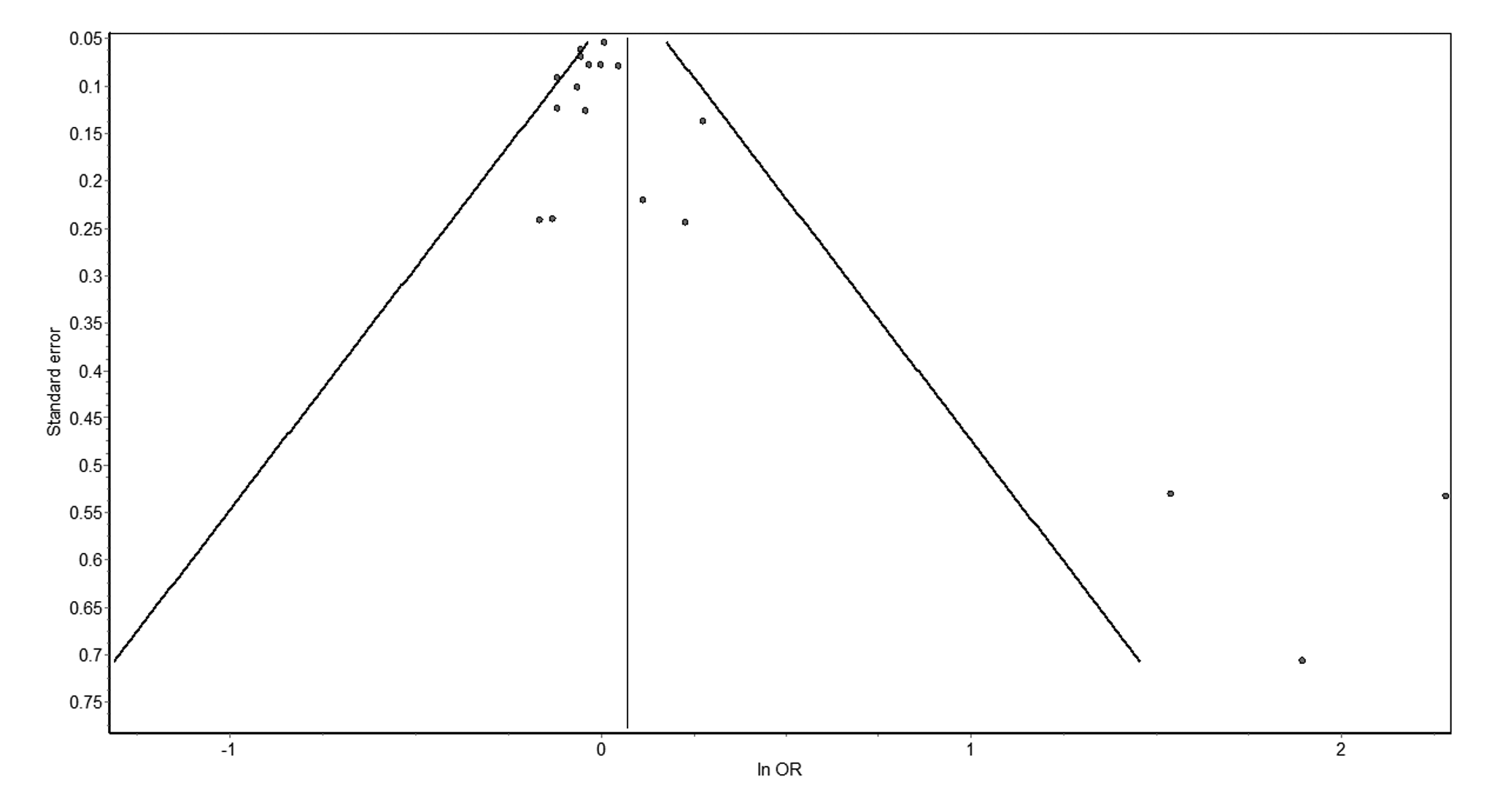

Supplement: Figure S35 — Funnel plot to aid assessment of publication bias for obesity and neglect. (TIF) [file pmed.1001349.s035.tif]

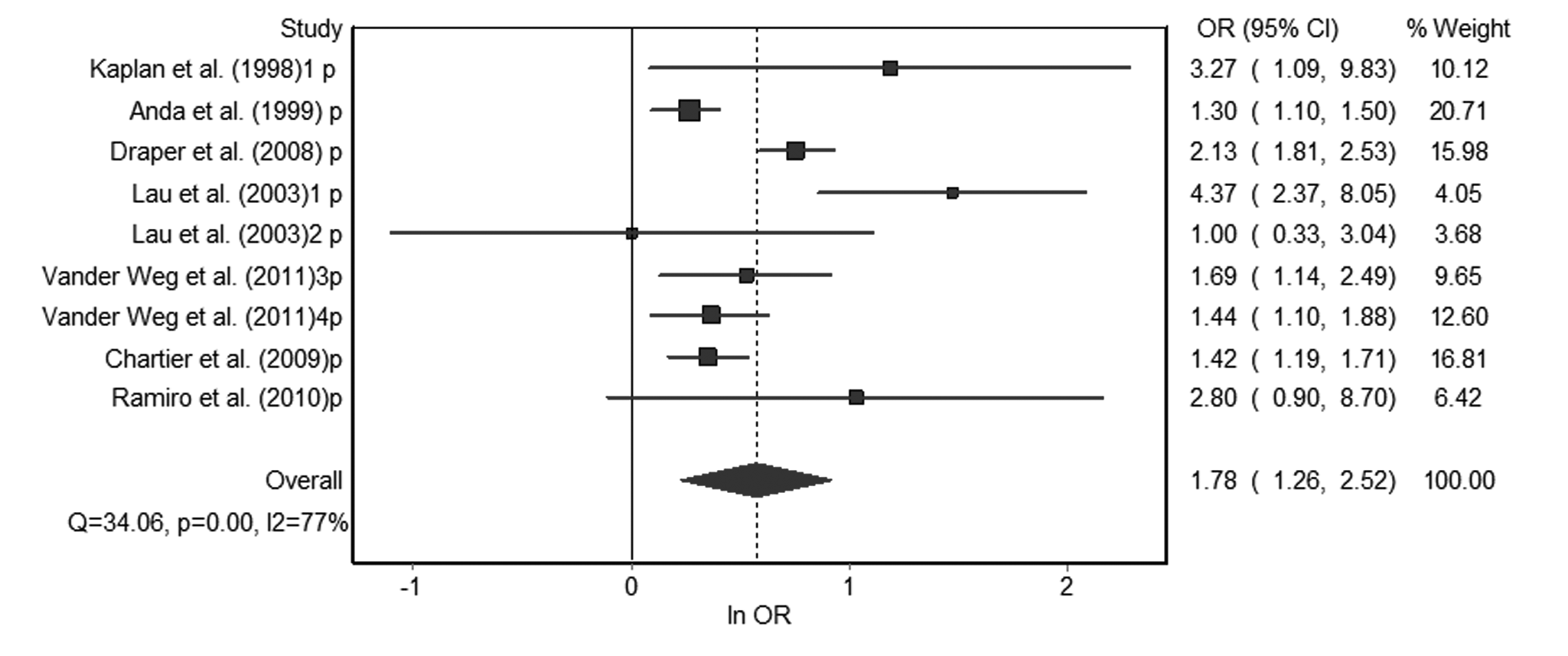

Supplement: Figure S36 — Forest plot for quality-effect meta-analysis of the association between physical abuse and current smoking. Studies are represented by symbols, the area of which is proportional to the study's weight in the analysis. Output for ORs is set to the (natural) log scale. (TIF) [file pmed.1001349.s036.tif]

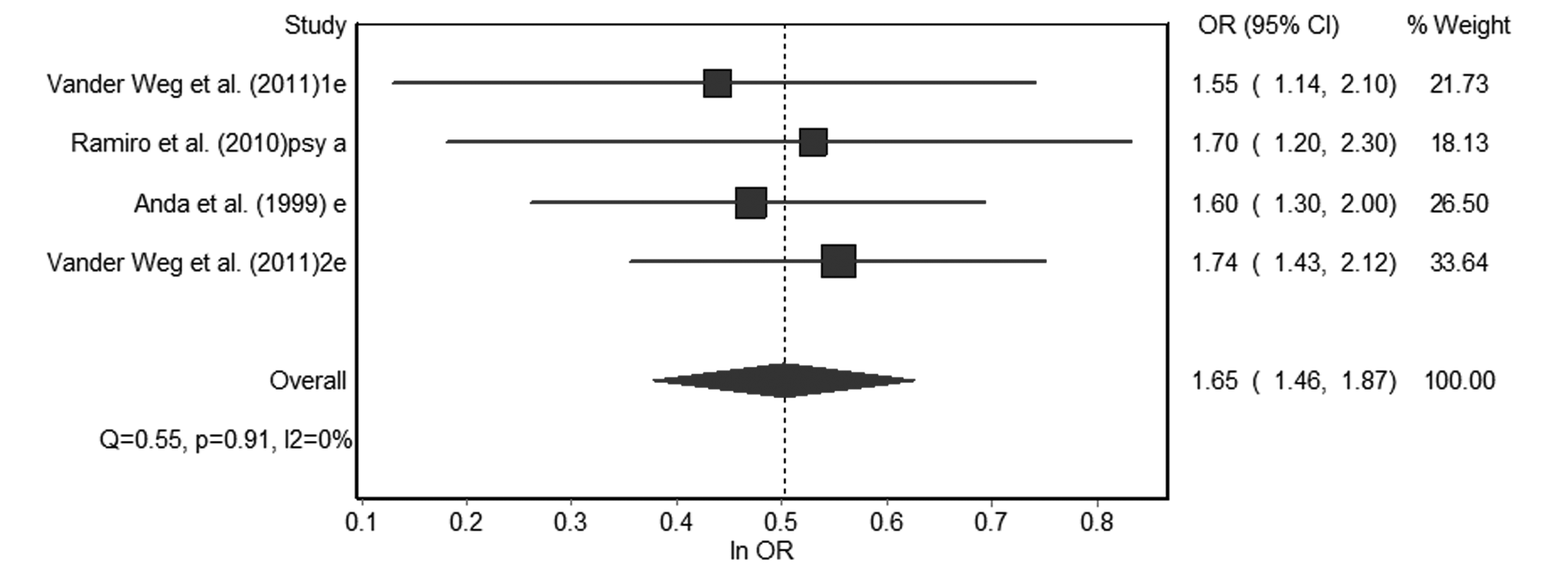

Supplement: Figure S37 — Forest plot for quality-effect meta-analysis of the association between emotional abuse and current smoking. Studies are represented by symbols, the area of which is proportional to the study's weight in the analysis. Output for ORs is set to the (natural) log scale. (TIF) [file pmed.1001349.s037.tif]

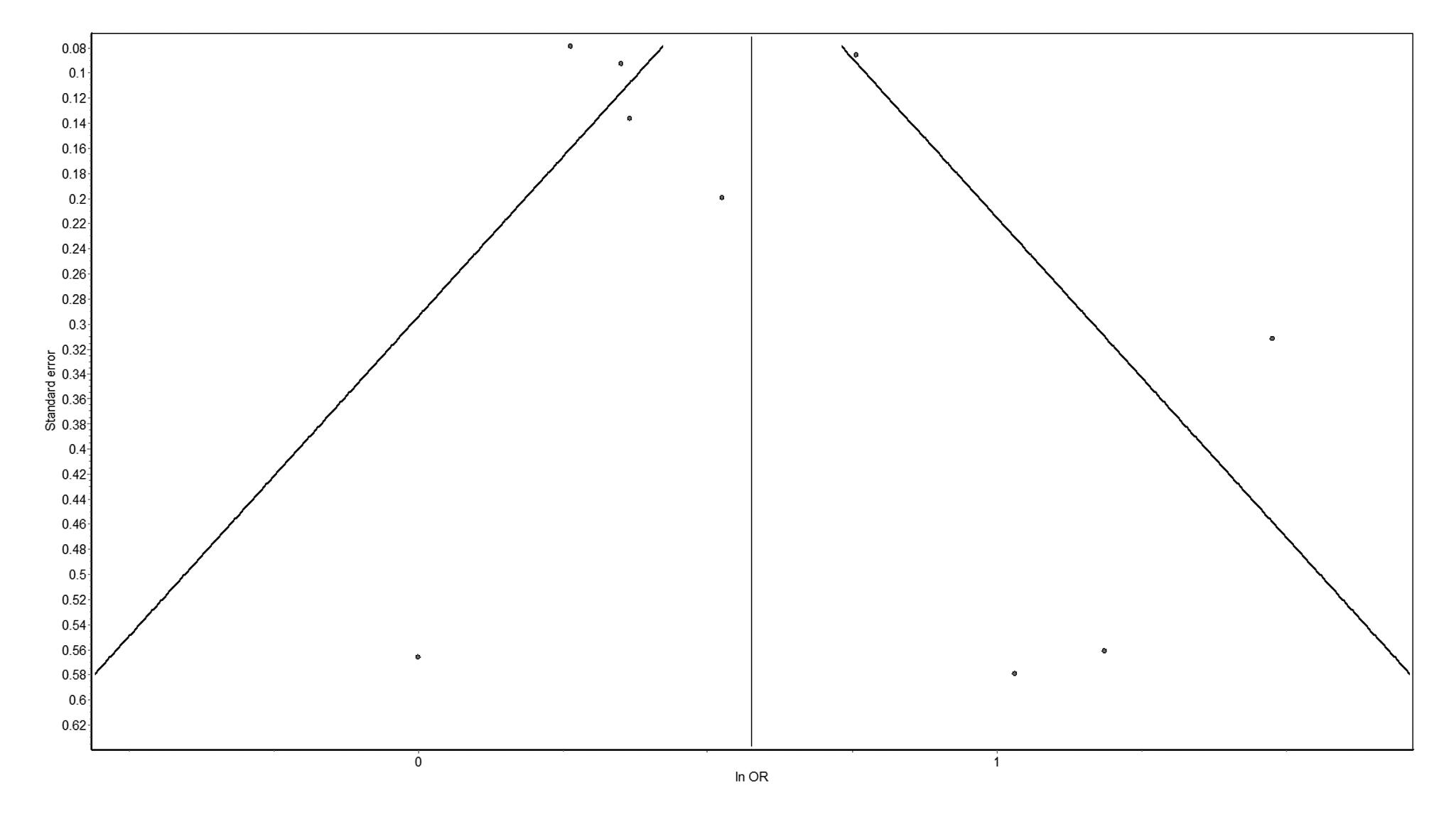

Supplement: Figure S38 — Funnel plot to aid assessment of publication bias for current smoking and physical abuse. (TIF) [file pmed.1001349.s038.tif]

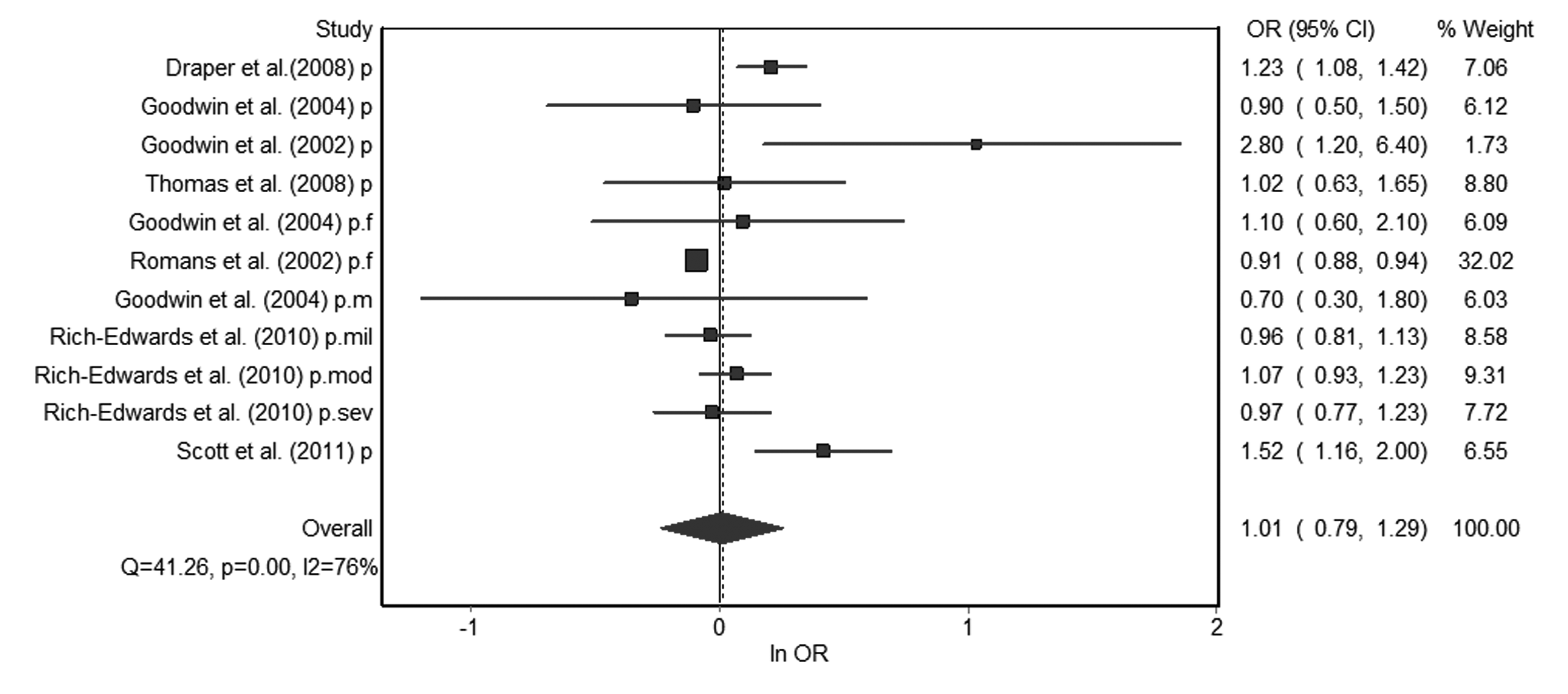

Supplement: Figure S39 — Forest plot for quality-effect meta-analysis of the association between physical abuse and type 2 diabetes. Studies are represented by symbols, the area of which is proportional to the study's weight in the analysis. Output for ORs is set to the (natural) log scale. (TIF) [file pmed.1001349.s039.tif]

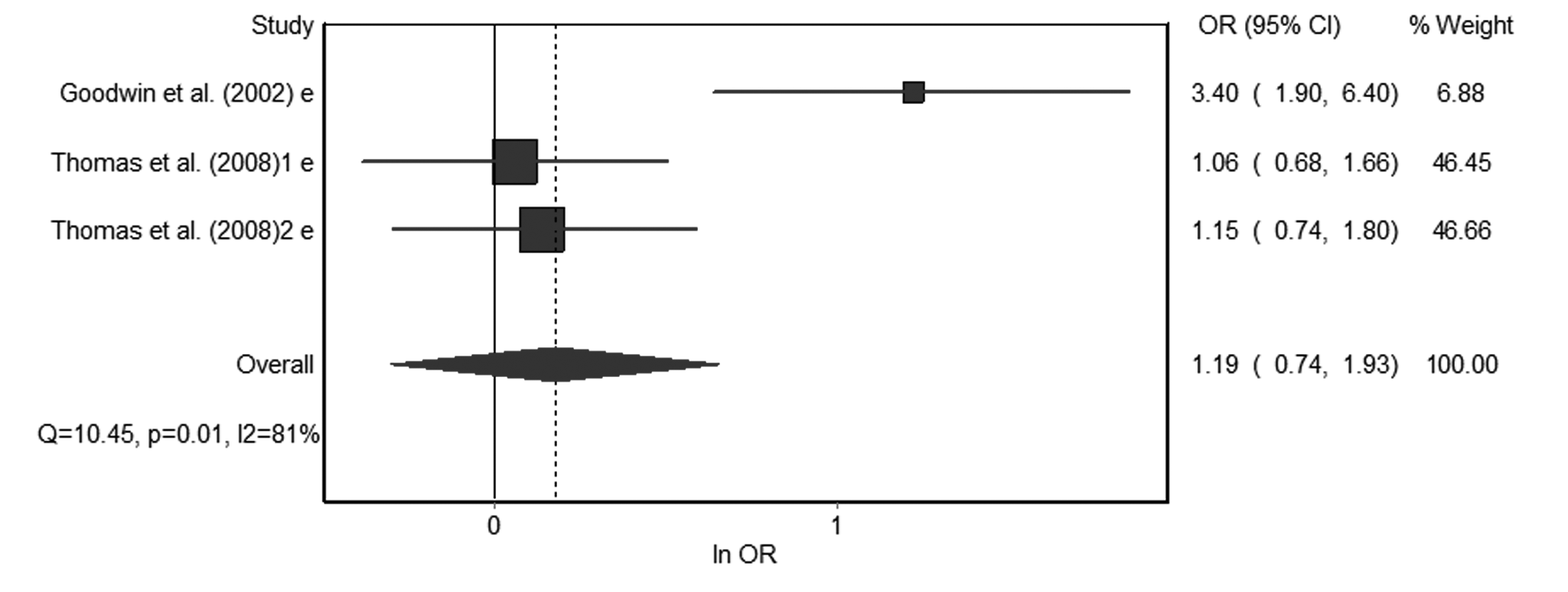

Supplement: Figure S40 — Forest plot for quality-effect meta-analysis of the association between emotional abuse and type 2 diabetes. Studies are represented by symbols, the area of which is proportional to the study's weight in the analysis. Output for ORs is set to the (natural) log scale. (TIF) [file pmed.1001349.s040.tif]

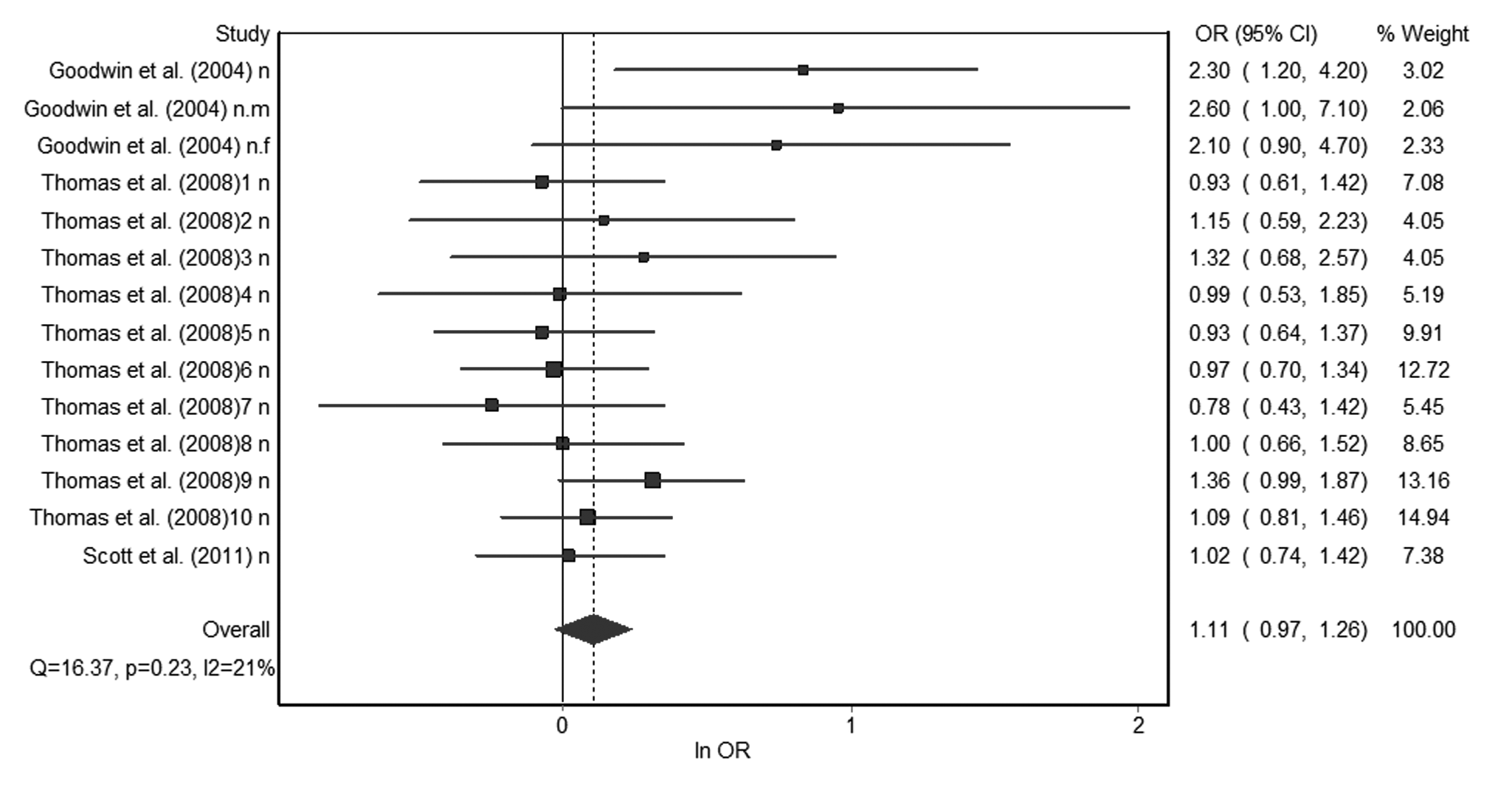

Supplement: Figure S41 — Forest plot for quality-effect meta-analysis of the association between neglect and type 2 diabetes. Studies are represented by symbols, the area of which is proportional to the study's weight in the analysis. Output for ORs is set to the (natural) log scale. (TIF) [file pmed.1001349.s041.tif]

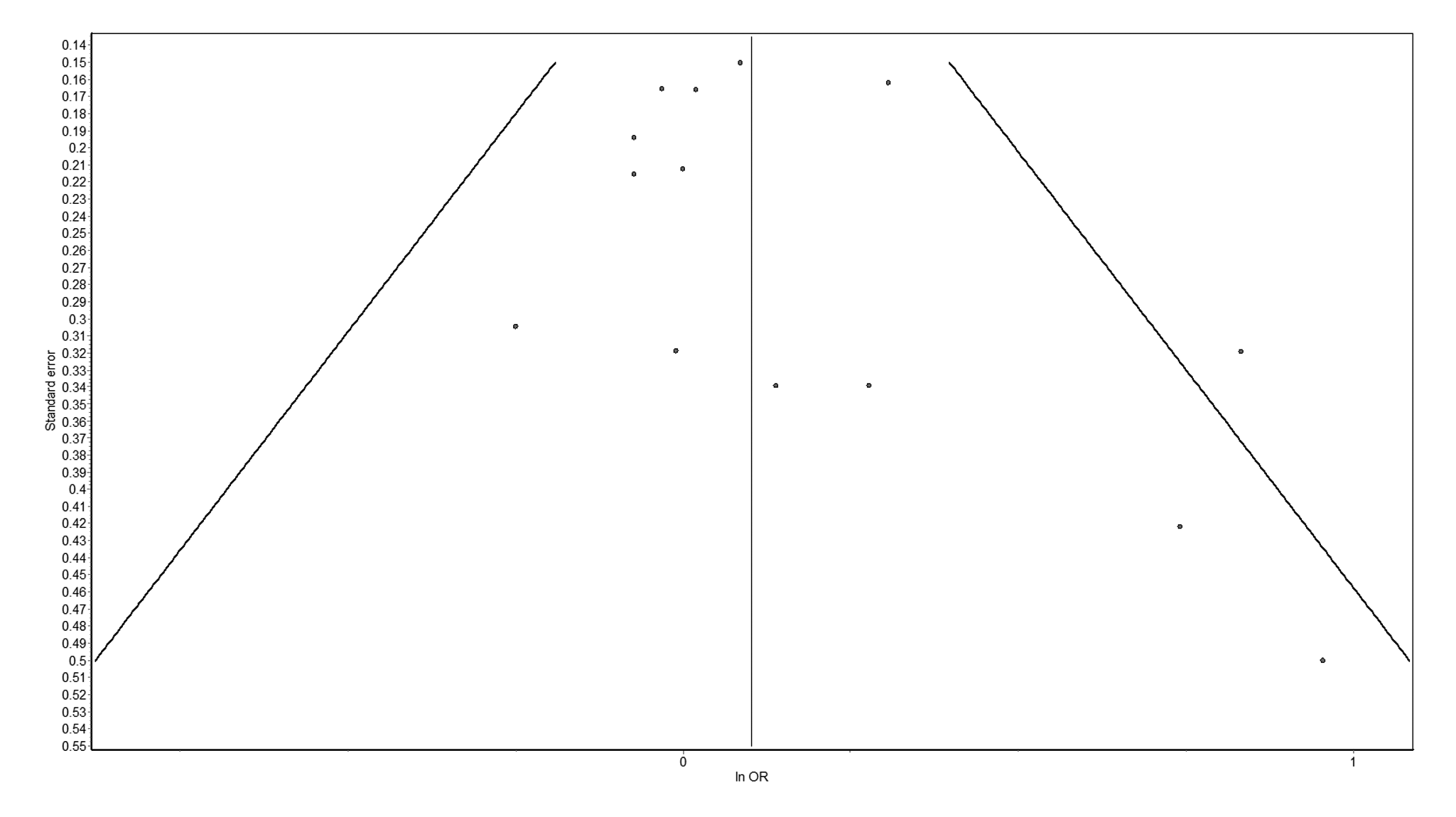

Supplement: Figure S42 — Funnel plot to aid assessment of publication bias for type 2 diabetes and neglect. (TIF) [file pmed.1001349.s042.tif]
