# Supplementary material for: The Long-Term Health Consequences of Child Physical Abuse, Emotional Abuse, and Neglect: A Systematic Review and Meta-Analysis
Source: PLoS Med. 2012 Nov 27;9(11):e1001349. doi: 10.1371/journal.pmed.1001349 (PMC3507962; doi:10.1371/journal.pmed.1001349)
Supplement: Table S1 — Depressive disorders subgroup analyses. (DOC) [file pmed.1001349.s043.doc]

Table S1 Depressive disorders subgroup analyses

|  | **No of data points** | **Pooled OR** | **95% LCI** | **95% UCI** | **Cochran's Q** | **I^2^** | **Test of heterogeneity**  **p-value** |
| --- | --- | --- | --- | --- | --- | --- | --- |
| **Primary analysis** |  |  |  |  |  |  |  |
| **Depressive disorders** |  |  |  |  |  |  |  |
| Physical abuse | 36 | 1.54 | 1.16 | 2.04 | 273.81 | 87.22 | <0.01 |
| Emotional abuse | 9 | 3.06 | 2.43 | 3.85 | 21.99 | 63.63 | <0.01 |
| Neglect | 14 | 2.11 | 1.61 | 2.77 | 45.33 | 71.32 | <0.01 |
| **Subgroup analyses** |  |  |  |  |  |  |  |
| **1. Gender** |  |  |  |  |  |  |  |
| ***Female*** |  |  |  |  |  |  |  |
| Physical abuse | 6 | 1.94 | 1.21 | 3.11 | 31.02 | 83.88 | <0.01 |
| Emotional abuse | 2 | 2.40 | 1.47 | 3.92 | 14.68 | 93.19 | <0.01 |
| Neglect | 2 | 1.49 | 1.03 | 2.16 | 1.46 | 31.37 | 0.23 |
| ***Male*** |  |  |  |  |  |  |  |
| Physical abuse | 2 | 2.33 | 1.29 | 4.21 | 1.99 | 49.77 | 0.16 |
| Emotional abuse | 1 | 3.30 | 2.40 | 4.40 | not pooled | not pooled | not pooled |
| Neglect | 2 | 2.79 | 1.88 | 4.14 | 0.86 | 0.00 | 0.35 |
| **2. Sample type** |  |  |  |  |  |  |  |
| ***Population based*** |  |  |  |  |  |  |  |
| Physical abuse | 28 | 1.56 | 1.15 | 2.12 | 170.20 | 84.14 | <0.01 |
| Emotional abuse | 4 | 2.51 | 1.71 | 3.68 | 12.13 | 75.27 | 0.01 |
| Neglect | 8 | 3.06 | 2.16 | 4.34 | 11.88 | 41.10 | 0.10 |
| ***Non-representative*** |  |  |  |  |  |  |  |
| Physical abuse | 8 | 1.80 | 1.22 | 2.67 | 31.62 | 77.86 | <0.01 |
| Emotional abuse | 5 | 3.52 | 2.89 | 4.30 | 3.02 | 0.00 | 0.56 |
| Neglect | 6 | 1.60 | 1.25 | 2.05 | 12.66 | 60.51 | 0.03 |
| **3. Assessment of exposure** |  |  |  |  |  |  |  |
| ***Prospective*** |  |  |  |  |  |  |  |
| Physical abuse | 4 | 2.35 | 1.29 | 4.25 | 7.59 | 60.48 | 0.06 |
| Neglect | 3 | 1.57 | 1.05 | 2.35 | 5.56 | 64.02 | 0.06 |
| ***Retrospective*** |  |  |  |  |  |  |  |
| Physical abuse | 32 | 1.47 | 1.09 | 1.98 | 260.92 | 88.12 | <0.01 |
| - Females | 5 | 1.89 | 1.08 | 3.31 | 30.96 | 87.08 | <0.01 |
| - Males | 2 | 2.32 | 1.27 | 4.25 | 1.90 | 47.27 | 0.17 |
| Emotional abuse | 9 | 3.06 | 2.43 | 3.85 | 21.99 | 63.63 | <0.01 |
| Neglect | 11 | 2.43 | 1.77 | 3.34 | 27.14 | 63.15 | <0.01 |
| **4. Measurement of physical abuse** |  |  |  |  |  |  |  |
| Official records | 4 | 2.41 | 1.32 | 4.41 | 8.17 | 63.27 | 0.04 |
| Self reported physical abuse (excl. punishment) | 24 | 1.56 | 1.11 | 2.19 | 199.25 | 88.46 | <0.01 |
| Self reported physical punishment | 8 | 1.20 | 0.88 | 1.61 | 40.47 | 82.70 | <0.01 |
| **5. Assessment of outcome** |  |  |  |  |  |  |  |
| ***Major depressive disorder***  ***(structured interview)*** |  |  |  |  |  |  |  |
| Physical abuse | 12 | 1.82 | 1.44 | 2.30 | 53.76 | 72.10 | <0.01 |
| Emotional abuse | 2 | 4.89 | 2.41 | 9.91 | 0.96 | 0.00 | 0.33 |
| Neglect | 3 | 1.45 | 1.11 | 1.90 | 2.23 | 10.38 | 0.33 |
| **Dysthymia**  ***(structured interview)*** |  |  |  |  |  |  |  |
| Physical punishment | 1 | 1.70 | 0.89 | 3.23 | not pooled | not pooled | not pooled |
| ***Depressive disorders (symptom scales/self-reported)*** |  |  |  |  |  |  |  |
| Physical abuse | 23 | 1.52 | 1.03 | 2.24 | 189.69 | 88.40 | <0.01 |
| Emotional abuse | 7 | 2.90 | 2.28 | 3.69 | 18.65 | 67.83 | <0.01 |
| Neglect | 11 | 2.63 | 1.88 | 3.68 | 28.86 | 65.35 | <0.01 |
| **6. Adjustment for confounders** |  |  |  |  |  |  |  |
| ***univariate analysis or controls for age/sex only*** |  |  |  |  |  |  |  |
| Physical abuse | 9 | 1.61 | 0.88 | 2.94 | 145.54 | 94.50 | <0.01 |
| ***adjusted for SES, education,family dysfunction*** |  |  |  |  |  |  |  |
| Physical abuse | 27 | 1.54 | 1.16 | 2.04 | 125.87 | 79.34 | <0.01 |
| **7. High income countries** |  |  |  |  |  |  |  |
| Physical abuse | 34 | 1.58 | 1.18 | 2.12 | 264.12 | 87.51 | <0.01 |
| Emotional abuse | 9 | 3.06 | 2.43 | 3.85 | 21.99 | 63.63 | <0.01 |
| Neglect | 10 | 2.11 | 1.53 | 2.90 | 36.92 | 75.62 | <0.01 |
| **Low to middle-income countries** |  |  |  |  |  |  |  |
| Physical punishment | 2 | 0.58 | 0.30 | 1.12 | 3.20 | 68.72 | 0.07 |
| Neglect | 4 | 1.98 | 1.41 | 2.78 | 7.88 | 61.91 | 0.05 |
| **8. Dose-response relationship*** |  |  |  |  |  |  |  |
| Physical abuse sometimes | 2 | 1.00 | 0.44 | 2.25 | 10.73 | 90.68 | <0.01 |
| Physical abuse often | 2 | 1.55 | 0.76 | 3.18 | 6.78 | 85.26 | 0.01 |
| Emotional abuse sometimes | 1 | 3.23 | 2.00 | 5.21 | not pooled | not pooled | not pooled |
| Emotional abuse often | 1 | 3.79 | 2.07 | 6.95 | not pooled | not pooled | not pooled |
| Neglect sometimes | 3 | 2.33 | 1.47 | 3.70 | 13.37 | 85.04 | <0.01 |
| Neglect often | 3 | 3.10 | 2.02 | 4.76 | 8.11 | 75.33 | 0.02 |

*Dose-response relationship data sources: Jewkes et al. [13] and Hovens et al. [22]
