# Supplementary material for: The Long-Term Health Consequences of Child Physical Abuse, Emotional Abuse, and Neglect: A Systematic Review and Meta-Analysis
Source: PLoS Med. 2012 Nov 27;9(11):e1001349. doi: 10.1371/journal.pmed.1001349 (PMC3507962; doi:10.1371/journal.pmed.1001349)
Supplement: Table S2 — Anxiety disorders subgroup analyses. (DOC) [file pmed.1001349.s044.doc]

Table S2 Anxiety disorders subgroup analyses

|  | **No of data points** | | **Pooled OR** | **95% LCI** | **95% UCI** | **Cochran's Q** | **I^2^** | **Test of heterogeneity**  **p-value** |
| --- | --- | --- | --- | --- | --- | --- | --- | --- |
| **Primary analysis** |  | |  |  |  |  |  |  |
| **Anxiety disorders** |  | |  |  |  |  |  |  |
| Physical abuse | 59 | | 1.51 | 1.27 | 1.79 | 592.99 | 90.22 | <0.01 |
| Emotional abuse | 4 | | 3.21 | 2.05 | 5.03 | 43.17 | 93.05 | <0.01 |
| Neglect | 8 | | 1.82 | 1.51 | 2.20 | 11.24 | 37.74 | 0.13 |
| **Subgroup analyses** |  | |  |  |  |  |  |  |
| **1. Gender** |  | |  |  |  |  |  |  |
| Physical abuse |  | |  |  |  |  |  |  |
| - Females | 15 | | 2.05 | 1.53 | 2.73 | 145.52 | 90.38 | <0.01 |
| - Males | 13 | | 2.05 | 1.59 | 2.65 | 37.53 | 68.03 | <0.01 |
| Emotional abuse |  | |  |  |  |  |  |  |
| - Females | 2 | | 3.95 | 2.25 | 6.95 | 34.60 | 97.11 | <0.01 |
| **2. Assessment of outcome** |  | |  |  |  |  |  |  |
| **Anxiety disorders (self-reported, symptom scales)** |  |  | |  |  |  |  |  |
| Physical abuse | 6 | | 3.07 | 2.04 | 4.62 | 46.81 | 91.96 | <0.01 |
| Emotional abuse | 2 | | 3.95 | 2.25 | 6.95 | 34.60 | 97.11 | <0.01 |
| ***Anxiety disorders (structured interview)*** |  | |  |  |  |  |  |  |
| Physical abuse | 31 | | 1.34 | 1.09 | 1.64 | 138.78 | 78.38 | <0.01 |
| Emotional abuse | 2 | | 1.84 | 1.19 | 2.86 | 0.25 | 0.00 | 0.62 |
| Neglect | 5 | | 1.95 | 1.30 | 2.91 | 11.15 | 64.11 | 0.02 |
| ***PTSD (structured interview)*** |  | |  |  |  |  |  |  |
| Physical abuse | 16 | | 2.94 | 2.25 | 3.84 | 91.53 | 83.61 | <0.01 |
| Neglect | 3 | | 1.73 | 1.44 | 2.08 | 0.10 | 0.00 | 0.95 |
| ***Panic disorder (structured interview)*** |  | |  |  |  |  |  |  |
| Physical abuse | 6 | | 1.69 | 1.34 | 2.13 | 6.01 | 16.77 | 0.31 |
| **3. Sample type** |  | |  |  |  |  |  |  |
| ***Population based*** |  | |  |  |  |  |  |  |
| Physical abuse | 53 | | 1.49 | 1.24 | 1.80 | 562.39 | 90.75 | <0.01 |
| - Females | 14 | | 2.06 | 1.52 | 2.78 | 145.41 | 91.06 | <0.01 |
| - Males | 12 | | 2.01 | 1.55 | 2.62 | 36.35 | 69.74 | <0.01 |
| Emotional abuse | 4 | | 3.21 | 2.05 | 5.03 | 43.17 | 93.05 | <0.01 |
| Neglect | 5 | | 1.95 | 1.30 | 2.91 | 11.15 | 64.11 | 0.02 |
| ***Non-representative*** |  | |  |  |  |  |  |  |
| Physical abuse | 6 | | 2.36 | 1.76 | 3.17 | 6.28 | 20.32 | 0.28 |
| Neglect | 3 | | 1.73 | 1.44 | 2.08 | 0.10 | 0.00 | 0.95 |
| **4. Assessment of exposure** |  | |  |  |  |  |  |  |
| ***Prospective*** |  | |  |  |  |  |  |  |
| Physical abuse | 4 | | 2.02 | 1.44 | 2.83 | 1.08 | 0.00 | 0.78 |
| Neglect | 4 | | 1.86 | 1.53 | 2.25 | 2.20 | 0.00 | 0.53 |
| ***Retrospective*** |  | |  |  |  |  |  |  |
| Physical abuse | 55 | | 1.51 | 1.27 | 1.78 | 584.49 | 90.76 | <0.01 |
| - Females | 15 | | 2.05 | 1.53 | 2.73 | 145.52 | 90.38 | <0.01 |
| - Males | 13 | | 2.05 | 1.59 | 2.65 | 37.53 | 68.03 | <0.01 |
| Emotional abuse | 4 | | 3.21 | 2.05 | 5.03 | 43.17 | 93.05 | <0.01 |
| Neglect | 4 | | 1.67 | 1.22 | 2.28 | 8.93 | 66.40 | 0.03 |
| **5. Dose-response*** |  | |  |  |  |  |  |  |
| Physical abuse sometimes | 1 | | 1.78 | 0.89 | 3.57 | not pooled | not pooled | not pooled |
| Physical abuse often | 1 | | 2.90 | 1.31 | 6.45 | not pooled | not pooled | not pooled |
| Emotional abuse sometimes | 1 | | 2.03 | 1.19 | 3.47 | not pooled | not pooled | not pooled |
| Emotional abuse often | 1 | | 1.61 | 0.77 | 3.36 | not pooled | not pooled | not pooled |
| Neglect sometimes | 1 | | 2.65 | 1.68 | 4.17 | not pooled | not pooled | not pooled |
| Neglect often | 1 | | 2.23 | 1.34 | 3.72 | not pooled | not pooled | not pooled |

*Dose-response relationship data source: Hovens et al. [22]
