# Supplementary material for: The Long-Term Health Consequences of Child Physical Abuse, Emotional Abuse, and Neglect: A Systematic Review and Meta-Analysis
Source: PLoS Med. 2012 Nov 27;9(11):e1001349. doi: 10.1371/journal.pmed.1001349 (PMC3507962; doi:10.1371/journal.pmed.1001349)
Supplement: Table S3 — Eating disorders subgroup analyses. (DOC) [file pmed.1001349.s045.doc]

Table S3 Eating disorders subgroup analyses

|  | **No of data points** | **Pooled OR** | **95% LCI** | | **95% UCI** | **Cochran's Q** | **I^2^** | **Test of heterogeneity**  **p-value** |
| --- | --- | --- | --- | --- | --- | --- | --- | --- |
| **Primary analysis** |  |  |  | |  |  |  |  |
| **Any eating disorder** |  |  |  | |  |  |  |  |
| Physical abuse | 6 | 2.58 | 1.17 | | 5.70 | 43.66 | 88.55 | <0.01 |
| Emotional abuse | 2 | 2.56 | 1.41 | | 4.65 | 4.40 | 77.27 | 0.04 |
| Neglect | 2 | 2.99 | 1.53 | | 5.83 | 2.14 | 53.33 | 0.14 |
| **Subgroup analyses** |  |  |  | |  |  |  |  |
| **1. Gender** |  |  |  | |  |  |  |  |
| ***Female*** |  |  |  | |  |  |  |  |
| Physical abuse | 6 | 2.58 | 1.17 | | 5.70 | 43.66 | 88.55 | <0.01 |
| Emotional abuse | 2 | 2.56 | 1.41 | | 4.65 | 4.40 | 77.27 | 0.04 |
| Neglect | 1 | 2.02 | 1.06 | | 3.83 | not pooled | not pooled | not pooled |
| **2. Eating disorder type** |  |  |  | |  |  |  |  |
| ***Eating disorder not specified*** |  |  |  | |  |  |  |  |
| Emotional abuse | 1 | 1.76 | 1.17 | | 2.61 | not pooled | not pooled | not pooled |
| Neglect | 1 | 4.82 | 1.82 | | 12.73 | not pooled | not pooled | not pooled |
| ***Bulimia nervosa*** |  |  |  | |  |  |  |  |
| Physical abuse (females) | 5 | 5.18 | 2.84 | | 9.44 | 13.87 | 71.15 | 0.01 |
| Emotional abuse | 1 | 4.25 | 2.07 | | 8.73 | not pooled | not pooled | not pooled |
| Neglect | 1 | 2.02 | 1.06 | | 3.83 | not pooled | not pooled | not pooled |
| **3. Sample type** |  |  |  | |  |  |  |  |
| ***Non-representative*** |  |  |  | |  |  |  |  |
| Physical abuse (females) | 1 | 1.19 | 1.17 | | 1.20 | not pooled | not pooled | not pooled |
| ***Population based*** |  |  |  | |  |  |  |  |
| Physical abuse (females) | 5 | 5.18 | 2.84 | | 9.44 | 13.87 | 71.15 | 0.01 |
| Emotional abuse (females) | 2 | 2.56 | 1.41 | | 4.65 | 4.40 | 77.27 | 0.04 |
| Neglect | 2 | 2.99 | 1.53 | | 5.83 | 2.14 | 53.33 | 0.14 |
| **4. Assessment of exposure** |  |  |  | |  |  |  |  |
| ***Prospective*** |  |  |  | |  |  |  |  |
| Neglect | 1 | 4.82 | 1.82 | | 12.73 | not pooled | not pooled | not pooled |
| ***Retrospective*** |  |  |  | |  |  |  |  |
| Physical abuse (females) | 6 | 2.58 | 1.17 | | 5.70 | 43.66 | 88.55 | <0.01 |
| Emotional abuse (females) | 2 | 2.56 | 1.41 | | 4.65 | 4.40 | 77.27 | 0.04 |
| Neglect (females) | 1 | 2.02 | 1.06 | | 3.83 | not pooled | not pooled | not pooled |
| **5. Dose-response relationship* (Bulimia nervosa, females)** |  |  | | |  |  |  |  |
| any physical abuse | 1 | 5.95 | | 3.25 | 19.31 | not pooled | not pooled | not pooled |
| severe physical abuse | 1 | 10.00 | | 2.19 | 45.64 | not pooled | not pooled | not pooled |
| repeated physical abuse | 1 | 7.92 | | 3.25 | 19.31 | not pooled | not pooled | not pooled |
| repeated severe physical abuse | 1 | 16.00 | | 2.00 | 127.90 | not pooled | not pooled | not pooled |

*Dose-response relationship data source: Welch et al. [24]
