# Supplementary material for: The Long-Term Health Consequences of Child Physical Abuse, Emotional Abuse, and Neglect: A Systematic Review and Meta-Analysis
Source: PLoS Med. 2012 Nov 27;9(11):e1001349. doi: 10.1371/journal.pmed.1001349 (PMC3507962; doi:10.1371/journal.pmed.1001349)
Supplement: Table S4 — Childhood behavioural/conduct disorders subgroup analyses. (DOC) [file pmed.1001349.s046.doc]

Table S4 Childhood behavioural/conduct disorders subgroup analyses

|  | **No of data points** | **Pooled OR** | **95% LCI** | **95% UCI** | **Cochran's Q** | **I^2^** | **Test of heterogeneity**  **p-value** |
| --- | --- | --- | --- | --- | --- | --- | --- |
| **Primary analysis** |  |  |  |  |  |  |  |
| **Childhood behavioural/conduct disorders** |  |  |  |  |  |  |  |
| Physical abuse | 12 | 2.29 | 1.76 | 2.97 | 15.83 | 30.53 | 0.15 |
| Neglect | 6 | 2.01 | 1.42 | 2.84 | 2.02 | 0.00 | 0.85 |
| **Subgroup analyses** |  |  |  |  |  |  |  |
| **1. Conduct disorder type** |  |  |  |  |  |  |  |
| ***Conduct disorder*** |  |  |  |  |  |  |  |
| Physical abuse | 3 | 1.93 | 1.03 | 3.61 | 8.19 | 75.58 | 0.02 |
| Neglect | 1 | 2.30 | 0.70 | 7.70 | not pooled | not pooled | not pooled |
| ***Childhood behavioural*** |  |  |  |  |  |  |  |
| Physical abuse | 9 | 2.40 | 1.82 | 3.16 | 5.84 | 0.00 | 0.67 |
| Neglect | 5 | 1.99 | 1.39 | 2.87 | 1.92 | 0.00 | 0.75 |
| **2. Sample type** |  |  |  |  |  |  |  |
| ***Non-representative*** |  |  |  |  |  |  |  |
| Physical abuse | 3 | 5.98 | 2.73 | 13.10 | 0.82 | 0.00 | 0.66 |
| Neglect | 1 | 2.30 | 0.70 | 7.70 | not pooled | not pooled | not pooled |
| ***Population based*** |  |  |  |  |  |  |  |
| Physical abuse | 9 | 2.02 | 1.58 | 2.58 | 7.81 | 0.00 | 0.45 |
| Neglect | 5 | 1.99 | 1.39 | 2.87 | 1.92 | 0.00 | 0.75 |
| **3. Assessment of exposure** |  |  |  |  |  |  |  |
| ***Prospective*** |  |  |  |  |  |  |  |
| Physical abuse | 4 | 3.13 | 1.64 | 5.95 | 1.37 | 0.00 | 0.71 |
| Neglect | 3 | 1.72 | 0.86 | 3.46 | 0.67 | 0.00 | 0.71 |
| ***Retrospective*** |  |  |  |  |  |  |  |
| Physical abuse | 8 | 2.17 | 1.59 | 2.94 | 11.89 | 41.14 | 0.10 |
| Neglect | 3 | 2.32 | 1.50 | 3.58 | 1.33 | 0.00 | 0.51 |
