# Supplementary material for: The Long-Term Health Consequences of Child Physical Abuse, Emotional Abuse, and Neglect: A Systematic Review and Meta-Analysis
Source: PLoS Med. 2012 Nov 27;9(11):e1001349. doi: 10.1371/journal.pmed.1001349 (PMC3507962; doi:10.1371/journal.pmed.1001349)
Supplement: Table S5 — Alcohol use subgroup analyses. (DOC) [file pmed.1001349.s047.doc]

Table S5 Alcohol use subgroup analyses

|  | **No of data points** | **Pooled OR** | **95% LCI** | **95% UCI** | **Cochran's Q** | **I^2^** | **Test of heterogeneity**  **p-value** |
| --- | --- | --- | --- | --- | --- | --- | --- |
| **Primary analysis** |  |  |  |  |  |  |  |
| **Alcohol use** |  |  |  |  |  |  |  |
| Physical abuse (any alcohol use) | 44 | 1.30 | 1.10 | 1.55 | 207.27 | 79.25 | <0.01 |
| - Non-problem drinker | 11 | 1.47 | 1.17 | 1.85 | 32.87 | 69.57 | <0.01 |
| - Problem drinker | 33 | 1.26 | 1.03 | 1.55 | 153.20 | 79.11 | <0.01 |
| Emotional abuse (any alcohol use) | 10 | 1.27 | 1.11 | 1.46 | 13.26 | 32.12 | 0.15 |
| - Non-problem drinker | 2 | 1.29 | 0.88 | 1.90 | 4.28 | 76.62 | 0.04 |
| - Problem drinker | 8 | 1.27 | 1.11 | 1.46 | 8.58 | 18.38 | 0.28 |
| Neglect (any alcohol use) | 15 | 1.14 | 0.92 | 1.39 | 100.32 | 86.04 | <0.01 |
| - Non-problem drinker | 4 | 1.50 | 1.15 | 1.96 | 15.14 | 80.18 | <0.01 |
| - Problem drinker | 11 | 1.09 | 0.87 | 1.35 | 50.38 | 80.15 | <0.01 |
| **Subgroup analyses** |  |  |  |  |  |  |  |
| **1. Gender**  **(alcohol problem drinker)** |  |  |  |  |  |  |  |
| ***Female*** |  |  |  |  |  |  |  |
| Physical abuse | 9 | 1.22 | 0.86 | 1.74 | 19.51 | 58.99 | 0.01 |
| Emotional abuse | 2 | 1.30 | 0.89 | 1.88 | 5.29 | 81.11 | 0.02 |
| Neglect | 5 | 1.58 | 1.28 | 1.95 | 1.12 | 0.00 | 0.89 |
| ***Male*** |  |  |  |  |  |  |  |
| Physical abuse | 9 | 1.38 | 1.10 | 1.74 | 20.33 | 60.65 | 0.01 |
| Emotional abuse | 3 | 1.43 | 1.18 | 1.73 | 0.56 | 0.00 | 0.76 |
| Neglect | 3 | 1.04 | 0.72 | 1.48 | 8.69 | 76.98 | 0.01 |
| **2. Sample type** |  |  |  |  |  |  |  |
| ***Population based*** |  |  |  |  |  |  |  |
| Physical abuse | 24 | 1.30 | 1.00 | 1.69 | 123.08 | 81.31 | <0.01 |
| - Females | 4 | 1.32 | 0.96 | 1.82 | 2.13 | 0.00 | 0.55 |
| - Males | 3 | 1.65 | 0.97 | 2.80 | 8.11 | 75.35 | 0.02 |
| Emotional abuse | 6 | 1.22 | 0.98 | 1.51 | 6.70 | 25.42 | 0.24 |
| Neglect | 8 | 1.42 | 1.23 | 1.64 | 9.28 | 24.55 | 0.23 |
| ***Non-representative*** |  |  |  |  |  |  |  |
| Physical abuse | 20 | 1.30 | 1.12 | 1.52 | 68.82 | 72.39 | <0.01 |
| - Females | 8 | 1.27 | 0.96 | 1.67 | 25.37 | 72.41 | <0.01 |
| - Males | 8 | 1.27 | 1.06 | 1.52 | 14.81 | 52.73 | 0.04 |
| Neglect | 7 | 1.07 | 0.77 | 1.50 | 50.66 | 88.16 | <0.01 |
| - Females | 3 | 1.56 | 1.10 | 2.19 | 1.03 | 0.00 | 0.60 |
| Emotional abuse | 4 | 1.33 | 1.15 | 1.55 | 6.30 | 52.39 | 0.10 |
| **3. Assessment of exposure (alcohol problem drinker)** |  |  |  |  |  |  |  |
| ***Prospective*** |  |  |  |  |  |  |  |
| Physical abuse | 2 | 0.84 | 0.54 | 1.31 | 0.00 | 0.00 | 0.96 |
| Neglect | 2 | 0.97 | 0.60 | 1.57 | 5.19 | 80.73 | 0.02 |
| ***Retrospective*** |  |  |  |  |  |  |  |
| Physical abuse | 31 | 1.30 | 1.04 | 1.63 | 150.17 | 80.02 | <0.01 |
| - Females | 10 | 1.31 | 0.90 | 1.91 | 25.63 | 64.88 | <0.01 |
| - Males | 9 | 1.46 | 1.16 | 1.83 | 21.28 | 62.41 | 0.01 |
| Emotional abuse | 8 | 1.27 | 1.11 | 1.46 | 8.58 | 18.38 | 0.28 |
| Neglect | 9 | 1.14 | 0.87 | 1.50 | 45.18 | 82.29 | <0.01 |
| **4. High income countries** |  |  |  |  |  |  |  |
| Physical abuse | 40 | 1.32 | 1.11 | 1.56 | 199.78 | 80.48 | <0.01 |
| Emotional abuse | 5 | 1.29 | 1.12 | 1.48 | 9.14 | 56.22 | <0.01 |
| Neglect | 9 | 1.08 | 0.85 | 1.38 | 75.22 | 89.36 | <0.01 |
| **Low to middle income countries** |  |  |  |  |  |  |  |
| Physical abuse | 4 | 1.17 | 0.60 | 2.27 | 6.31 | 52.46 | 0.10 |
| Emotional abuse | 5 | 1.28 | 1.02 | 1.62 | 3.81 | 0.00 | 0.43 |
| Neglect | 6 | 1.49 | 1.14 | 1.94 | 9.58 | 47.81 | 0.09 |
| **5. Age of drinking initiation under 14 years** |  |  |  |  |  |  |  |
| Physical abuse | 2 | 2.16 | 1.86 | 2.50 | 0.09 | 0.00 | 0.76 |
| Emotional abuse | 1 | 2.30 | 1.70 | 3.00 | not pooled | not pooled | not pooled |
| Neglect | 2 | 2.28 | 1.90 | 2.72 | 0.90 | 0.00 | 0.34 |
| **6. Alcohol abuse/dependence diagnosis** |  |  |  |  |  |  |  |
| Physical abuse | 13 | 1.40 | 1.21 | 1.64 | 19.38 | 38.07 | 0.08 |
| **7. Dose-response relationship* (alcohol problem drinker)** |  |  |  |  |  |  |  |
| Emotional abuse sometimes | 1 | 1.53 | 1.12 | 2.09 | not pooled | not pooled | not pooled |
| Emotional abuse often | 1 | 1.48 | 1.02 | 2.15 | not pooled | not pooled | not pooled |
| Neglect sometimes | 1 | 2.17 | 0.99 | 4.72 | not pooled | not pooled | not pooled |
| Neglect often | 1 | 1.82 | 0.81 | 4.11 | not pooled | not pooled | not pooled |

*Dose-response relationship data source: Jewkes et al. [13]
