# Supplementary material for: The Long-Term Health Consequences of Child Physical Abuse, Emotional Abuse, and Neglect: A Systematic Review and Meta-Analysis
Source: PLoS Med. 2012 Nov 27;9(11):e1001349. doi: 10.1371/journal.pmed.1001349 (PMC3507962; doi:10.1371/journal.pmed.1001349)
Supplement: Table S6 — Drug use subgroup analyses. (DOC) [file pmed.1001349.s048.doc]

Table S6 Drug use subgroup analyses

|  | **No of data points** | **Pooled OR** | **95% LCI** | **95% UCI** | **Cochran's Q** | **I^2^** | **Test of heterogeneity**  **p-value** |
| --- | --- | --- | --- | --- | --- | --- | --- |
| **Primary analysis** |  |  |  |  |  |  |  |
| **Drug use** |  |  |  |  |  |  |  |
| Physical abuse | 43 | 1.92 | 1.67 | 2.20 | 136.06 | 69.13 | <0.01 |
| Emotional abuse | 8 | 1.41 | 1.11 | 1.79 | 30.51 | 77.06 | <0.01 |
| Neglect | 41 | 1.36 | 1.21 | 1.54 | 180.81 | 77.88 | <0.01 |
| **Subgroup analyses** |  |  |  |  |  |  |  |
| **1. Gender** |  |  |  |  |  |  |  |
| ***Female*** |  |  |  |  |  |  |  |
| Physical abuse | 8 | 1.72 | 1.38 | 2.14 | 11.50 | 39.13 | 0.12 |
| Emotional abuse | 2 | 0.95 | 0.69 | 1.30 | 0.10 | 0.00 | 0.75 |
| Neglect | 13 | 1.26 | 1.01 | 1.57 | 43.17 | 72.20 | <0.01 |
| ***Male*** |  |  |  |  |  |  |  |
| Physical abuse | 6 | 1.90 | 1.36 | 2.64 | 16.60 | 69.89 | 0.01 |
| Emotional abuse | 2 | 1.46 | 0.97 | 2.19 | 1.44 | 30.79 | 0.23 |
| Neglect | 15 | 1.34 | 1.12 | 1.61 | 47.81 | 70.72 | <0.01 |
| **2. Sample type** |  |  |  |  |  |  |  |
| ***Population based*** |  |  |  |  |  |  |  |
| Physical abuse | 22 | 1.92 | 1.57 | 2.34 | 80.50 | 73.91 | <0.01 |
| Emotional abuse | 3 | 1.50 | 1.13 | 1.99 | 5.97 | 66.47 | 0.05 |
| Neglect | 7 | 1.12 | 0.94 | 1.35 | 21.36 | 71.91 | <0.01 |
| ***Non-representative*** |  |  |  |  |  |  |  |
| Physical abuse | 21 | 1.97 | 1.65 | 2.35 | 47.65 | 58.03 | <0.01 |
| - Females | 6 | 1.82 | 1.30 | 2.54 | 11.08 | 54.86 | 0.05 |
| - Males | 5 | 1.80 | 1.29 | 2.51 | 12.56 | 68.16 | 0.01 |
| Emotional abuse | 5 | 1.40 | 1.00 | 1.94 | 20.16 | 80.16 | <0.01 |
| Neglect | 34 | 1.45 | 1.27 | 1.66 | 134.07 | 75.39 | <0.01 |
| - Females | 12 | 1.25 | 0.98 | 1.60 | 42.24 | 73.96 | <0.01 |
| - Males | 14 | 1.43 | 1.20 | 1.71 | 33.09 | 60.71 | <0.01 |
| **3. Assessment of exposure** |  |  |  |  |  |  |  |
| ***Prospective*** |  |  |  |  |  |  |  |
| Physical abuse | 8 | 1.39 | 1.05 | 1.84 | 14.28 | 50.97 | 0.05 |
| Neglect | 8 | 1.20 | 1.01 | 1.43 | 10.26 | 31.77 | 0.17 |
| ***Retrospective*** |  |  |  |  |  |  |  |
| Physical abuse | 35 | 2.06 | 1.74 | 2.43 | 117.62 | 71.09 | <0.01 |
| - Females | 6 | 1.74 | 1.30 | 2.32 | 10.71 | 53.30 | 0.06 |
| - Males | 4 | 2.48 | 1.78 | 3.47 | 6.41 | 53.20 | 0.09 |
| Emotional abuse | 8 | 1.41 | 1.11 | 1.79 | 30.51 | 77.06 | <0.01 |
| Neglect | 33 | 1.40 | 1.22 | 1.61 | 167.45 | 80.89 | <0.01 |
| - Females | 11 | 1.21 | 0.94 | 1.55 | 41.12 | 75.68 | <0.01 |
| - Males | 13 | 1.43 | 1.17 | 1.75 | 43.20 | 72.22 | <0.01 |
| **4. High income** |  |  |  |  |  |  |  |
| Physical abuse | 40 | 1.87 | 1.64 | 2.15 | 118.50 | 67.09 | <0.01 |
| Emotional abuse | 7 | 1.34 | 1.03 | 1.74 | 27.55 | 78.22 | <0.01 |
| Neglect | 37 | 1.34 | 1.18 | 1.52 | 171.53 | 79.01 | <0.01 |
| **Low to middle income** |  |  |  |  |  |  |  |
| Physical abuse | 3 | 6.38 | 2.68 | 15.16 | 3.86 | 48.19 | 0.15 |
| Emotional abuse | 1 | 2.10 | 1.30 | 3.10 | not pooled | not pooled | not pooled |
| Neglect | 4 | 1.72 | 1.44 | 2.06 | 2.14 | 0.00 | 0.54 |
| **5. Dose-response relationship*** |  |  |  |  |  |  |  |
| Emotional abuse (males) | 1 | 1.20 | 0.80 | 1.90 | not pooled | not pooled | not pooled |
| Emotional abuse frequent (males) | 1 | 1.90 | 1.00 | 3.40 | not pooled | not pooled | not pooled |
| Emotional abuse (females) | 1 | 0.90 | 0.60 | 1.40 | not pooled | not pooled | not pooled |
| Emotional abuse frequent (females) | 1 | 1.00 | 0.60 | 1.60 | not pooled | not pooled | not pooled |
| Neglect | 9 | 0.98 | 0.80 | 1.21 | 17.04 | 53.05 | 0.03 |
| Neglect frequent | 9 | 1.20 | 0.98 | 1.47 | 10.90 | 26.63 | 0.21 |

*Dose-response relationship data sources: Jewkes et al. [13] and Conroy et al. [106]
