# Supplementary material for: The Long-Term Health Consequences of Child Physical Abuse, Emotional Abuse, and Neglect: A Systematic Review and Meta-Analysis
Source: PLoS Med. 2012 Nov 27;9(11):e1001349. doi: 10.1371/journal.pmed.1001349 (PMC3507962; doi:10.1371/journal.pmed.1001349)
Supplement: Table S7 — Suicidal behaviour subgroup analyses. (DOC) [file pmed.1001349.s049.doc]

Table S7 Suicidal behaviour subgroup analyses

|  | **No of data points** | **Pooled OR** | **95% LCI** | **95% UCI** | **Cochran's Q** | **I^2^** | **Test of heterogeneity**  **p-value** |
| --- | --- | --- | --- | --- | --- | --- | --- |
| **Primary analysis** |  |  |  |  |  |  |  |
| **Any suicidal behaviour** |  |  |  |  |  |  |  |
| Physical abuse | 58 | 3.00 | 2.07 | 4.33 | 2,392.41 | 97.62 | <0.01 |
| Emotional abuse | 11 | 3.08 | 2.42 | 3.93 | 32.36 | 69.10 | <0.01 |
| Neglect | 15 | 1.85 | 1.25 | 2.73 | 19.43 | 27.94 | 0.15 |
| **Subgroup analyses** |  |  |  |  |  |  |  |
| **1. Outcome and gender** |  |  |  |  |  |  |  |
| ***Suicide ideation*** |  |  |  |  |  |  |  |
| Physical abuse | 21 | 2.57 | 1.56 | 4.22 | 1,762.95 | 98.87 | <0.01 |
| - Females | 6 | 3.16 | 1.63 | 6.16 | 56.47 | 91.15 | <0.01 |
| - Males | 6 | 3.49 | 1.88 | 6.48 | 38.98 | 87.17 | <0.01 |
| Emotional abuse | 4 | 2.63 | 1.70 | 4.06 | 10.33 | 70.96 | 0.02 |
| Neglect | 4 | 1.95 | 1.12 | 3.38 | 8.41 | 64.35 | 0.04 |
| ***Suicide attempt*** |  |  |  |  |  |  |  |
| Physical abuse | 26 | 3.40 | 2.17 | 5.32 | 223.94 | 88.84 | <0.01 |
| - Females | 6 | 3.80 | 2.39 | 6.05 | 20.22 | 75.28 | <0.01 |
| - Males | 6 | 4.48 | 2.35 | 8.52 | 23.53 | 78.75 | <0.01 |
| Emotional abuse | 6 | 3.37 | 2.44 | 4.67 | 17.11 | 70.77 | <0.01 |
| Neglect | 8 | 1.95 | 1.13 | 3.37 | 8.46 | 17.27 | 0.29 |
| ***Self-inflicted injury*** |  |  |  |  |  |  |  |
| Physical abuse | 7 | 3.99 | 2.65 | 6.01 | 8.34 | 28.08 | 0.21 |
| Neglect | 2 | 1.45 | 0.42 | 5.07 | 1.73 | 42.26 | 0.19 |
| **Suicidal behaviour** |  |  |  |  |  |  |  |
| Physical abuse | 4 | 2.31 | 1.55 | 3.43 | 4.79 | 37.43 | 0.19 |
| Emotional abuse | 1 | 3.60 | 2.90 | 4.40 | not pooled | not pooled | not pooled |
| Neglect | 1 | 1.80 | 1.01 | 3.23 | not pooled | not pooled | not pooled |
| **2. Sample type** |  |  |  |  |  |  |  |
| ***Any suicidal behaviour (Population based)*** |  |  |  |  |  |  |  |
| Physical abuse | 43 | 3.07 | 2.00 | 4.71 | 2,163.24 | 98.06 | <0.01 |
| - Females | 13 | 3.42 | 2.29 | 5.12 | 89.14 | 86.54 | <0.01 |
| - Males | 14 | 3.76 | 2.32 | 6.09 | 141.03 | 90.78 | <0.01 |
| Emotional abuse | 4 | 1.66 | 1.11 | 2.49 | 4.39 | 31.73 | 0.22 |
| Neglect | 9 | 1.99 | 1.23 | 3.21 | 8.47 | 5.56 | 0.39 |
| ***Any suicidal behaviour (Non-representative)*** |  |  |  |  |  |  |  |
| Physical abuse | 15 | 2.49 | 1.70 | 3.65 | 59.48 | 76.46 | <0.01 |
| Emotional abuse | 7 | 4.05 | 3.39 | 4.84 | 8.80 | 31.80 | 0.19 |
| Neglect | 6 | 1.64 | 0.92 | 2.92 | 10.40 | 51.92 | 0.06 |
| **3. Assessment of exposure** |  |  |  |  |  |  |  |
| ***Self-inflicted injury (Prospective)*** |  |  |  |  |  |  |  |
| Physical abuse | 2 | 3.61 | 1.15 | 11.31 | 1.79 | 44.21 | 0.18 |
| Neglect | 2 | 1.45 | 0.42 | 5.07 | 1.73 | 42.26 | 0.19 |
| ***Any suicidal behaviour (Retrospective)*** |  |  |  |  |  |  |  |
| Physical abuse | 56 | 2.97 | 2.02 | 4.38 | 2,390.62 | 97.70 | <0.01 |
| - Females | 13 | 3.42 | 2.29 | 5.12 | 89.14 | 86.54 | <0.01 |
| - Males | 14 | 3.76 | 2.32 | 6.09 | 141.03 | 90.78 | <0.01 |
| Emotional abuse | 11 | 3.08 | 2.42 | 3.93 | 32.36 | 69.10 | <0.01 |
| Neglect | 13 | 1.90 | 1.25 | 2.89 | 17.38 | 30.95 | 0.14 |
| **4. Any suicidal behaviour**  **(High income countries)** |  |  |  |  |  |  |  |
| Physical abuse | 49 | 2.94 | 1.95 | 4.44 | 2,354.22 | 97.96 | <0.01 |
| Emotional abuse | 10 | 3.15 | 2.44 | 4.07 | 29.04 | 69.01 | <0.01 |
| Neglect | 10 | 1.52 | 0.94 | 2.46 | 8.83 | 0.00 | 0.45 |
| **Any suicidal behaviour (low-to-middle income countries)** |  |  |  |  |  |  |  |
| Physical abuse | 9 | 3.50 | 2.13 | 5.75 | 38.15 | 79.03 | <0.01 |
| Emotional abuse | 1 | 2.10 | 1.10 | 4.10 | not pooled | not pooled | not pooled |
| Neglect | 5 | 2.80 | 1.84 | 4.24 | 7.92 | 49.49 | 0.09 |
| **5. Dose-response relationship**  **(suicide ideation)*** |  |  |  |  |  |  |  |
| Neglect sometimes | 1 | 1.93 | 0.72 | 5.14 | not pooled | not pooled | not pooled |
| Neglect often | 1 | 5.07 | 2.07 | 12.45 | not pooled | not pooled | not pooled |

*Dose-response relationship data source: Jewkes et al. [13]
