# Supplementary material for: The Long-Term Health Consequences of Child Physical Abuse, Emotional Abuse, and Neglect: A Systematic Review and Meta-Analysis
Source: PLoS Med. 2012 Nov 27;9(11):e1001349. doi: 10.1371/journal.pmed.1001349 (PMC3507962; doi:10.1371/journal.pmed.1001349)
Supplement: Table S8 — Sexually transmitted infections and risky sexual behaviour subgroup analyses. (DOC) [file pmed.1001349.s050.doc]

Table S8 Sexually transmitted infections and risky sexual behavior subgroup analyses

|  | **No of data points** | **Pooled OR** | **95% LCI** | **95% UCI** | **Cochran's Q** | **I^2^** | **Test of heterogeneity**  **p-value** |
| --- | --- | --- | --- | --- | --- | --- | --- |
| **Primary analysis** |  |  |  |  |  |  |  |
| **Sexually transmitted infections/risky sexual behaviour** |  |  |  |  |  |  |  |
| Physical abuse | 33 | 1.78 | 1.50 | 2.10 | 49.12 | 34.85 | 0.03 |
| Emotional abuse | 5 | 1.75 | 1.49 | 2.04 | 2.96 | 0.00 | 0.57 |
| Neglect | 30 | 1.57 | 1.39 | 1.78 | 50.14 | 42.16 | 0.01 |
| **Subgroup analyses** |  |  |  |  |  |  |  |
| **1. Type of sexually transmitted infection** |  |  |  |  |  |  |  |
| ***HIV infection*** |  |  |  |  |  |  |  |
| Physical abuse | 4 | 2.51 | 1.16 | 5.42 | 1.09 | 0.00 | 0.78 |
| Emotional abuse | 2 | 1.82 | 1.34 | 2.47 | 0.21 | 0.00 | 0.65 |
| Neglect | 2 | 2.50 | 0.77 | 8.15 | 0.29 | 0.00 | 0.59 |
| ***Other sexually transmitted infections*** |  |  |  |  |  |  |  |
| Physical abuse | 12 | 1.53 | 1.13 | 2.07 | 17.27 | 7.65 | 0.10 |
| Emotional abuse | 2 | 1.56 | 1.26 | 1.93 | 0.76 | 0.00 | 0.38 |
| Neglect | 14 | 1.26 | 1.08 | 1.46 | 7.96 | 0.00 | 0.85 |
| ***Risky sexual behaviour*** |  |  |  |  |  |  |  |
| Physical abuse | 17 | 1.95 | 1.58 | 2.40 | 23.37 | 31.54 | 0.10 |
| Emotional abuse | 1 | 2.10 | 1.50 | 3.00 | not pooled | not pooled | not pooled |
| Neglect | 14 | 1.80 | 1.52 | 2.13 | 27.74 | 53.14 | 0.01 |
| **2. Gender** |  |  |  |  |  |  |  |
| **Female** |  |  |  |  |  |  |  |
| ***HIV infection*** |  |  |  |  |  |  |  |
| - Physical abuse | 2 | 1.82 | 1.05 | 3.15 | 0.37 | 0.00 | 0.54 |
| - Emotional abuse | 2 | 1.82 | 1.34 | 2.47 | 0.21 | 0.00 | 0.65 |
| ***Other sexually transmitted infection*** |  |  |  |  |  |  |  |
| - Physical abuse | 2 | 1.29 | 0.88 | 1.89 | 3.70 | 72.97 | 0.05 |
| - Emotional abuse | 1 | 1.70 | 1.30 | 2.20 | not pooled | not pooled | not pooled |
| - Neglect | 4 | 1.30 | 1.02 | 1.66 | 5.20 | 50.23 | 0.16 |
| ***Risky sexual behaviour*** |  |  |  |  |  |  |  |
| - Physical abuse | 3 | 1.13 | 0.68 | 1.87 | 1.68 | 0.00 | 0.43 |
| - Neglect | 2 | 1.72 | 0.94 | 3.15 | 2.01 | 50.23 | 0.16 |
| **Male** |  |  |  |  |  |  |  |
| ***Other sexually transmitted infections*** |  |  |  |  |  |  |  |
| - Physical abuse | 2 | 1.13 | 0.74 | 1.72 | 4.33 | 76.92 | 0.04 |
| - Emotional abuse | 1 | 1.40 | 1.00 | 2.00 | not pooled | not pooled | not pooled |
| - Neglect | 2 | 1.23 | 0.93 | 1.64 | 0.08 | 0.00 | 0.78 |
| ***Risky sexual behaviour*** |  |  |  |  |  |  |  |
| - Physical abuse | 3 | 1.30 | 0.76 | 2.24 | 5.14 | 61.09 | 0.08 |
| - Neglect | 2 | 1.22 | 0.90 | 1.66 | 0.78 | 0.00 | 0.38 |
| **3. Sample type** |  |  |  |  |  |  |  |
| ***Population based*** |  |  |  |  |  |  |  |
| Physical abuse | 6 | 1.28 | 0.88 | 1.86 | 11.44 | 56.31 | 0.04 |
| Emotional abuse | 1 | 2.10 | 1.50 | 3.00 | not pooled | not pooled | not pooled |
| Neglect | 6 | 1.41 | 1.13 | 1.76 | 14.24 | 64.89 | 0.01 |
| ***Non-representative*** |  |  |  |  |  |  |  |
| Physical abuse | 27 | 1.90 | 1.61 | 2.25 | 33.56 | 22.52 | 0.15 |
| Emotional abuse | 4 | 1.67 | 1.40 | 1.99 | 1.50 | 0.00 | 0.68 |
| Neglect | 24 | 1.61 | 1.40 | 1.85 | 35.45 | 35.12 | 0.05 |
| **4. Assessment of exposure** |  |  |  |  |  |  |  |
| ***Prospective*** |  |  |  |  |  |  |  |
| Physical abuse | 22 | 1.94 | 1.59 | 2.36 | 25.18 | 16.61 | 0.24 |
| Neglect | 22 | 1.61 | 1.39 | 1.86 | 34.83 | 39.70 | 0.03 |
| ***Retrospective*** |  |  |  |  |  |  |  |
| Physical abuse | 11 | 1.52 | 1.17 | 1.99 | 17.57 | 43.08 | 0.06 |
| Emotional abuse | 5 | 1.75 | 1.49 | 2.04 | 2.96 | 0.00 | 0.57 |
| Neglect | 8 | 1.42 | 1.18 | 1.70 | 14.71 | 52.41 | 0.04 |
| **5. Dose response relationship*** |  |  |  |  |  |  |  |
| Physical punishment sometimes (HIV infection) | 1 | 1.51 | 0.65 | 3.54 | not pooled | not pooled | not pooled |
| Physical punishment often (HIV infection) | 1 | 2.13 | 1.04 | 4.37 | not pooled | not pooled | not pooled |
| Emotional abuse sometimes (HIV infection) | 1 | 1.70 | 1.12 | 2.57 | not pooled | not pooled | not pooled |
| Emotional abuse often (HIV infection) | 1 | 1.96 | 1.25 | 3.06 | not pooled | not pooled | not pooled |
| Neglect sometimes (HSV2 infection) | 1 | 1.62 | 1.01 | 2.59 | not pooled | not pooled | not pooled |
| Neglect often (HSV2 infection) | 1 | 1.26 | 0.73 | 2.18 | not pooled | not pooled | not pooled |

*Dose-response relationship data source: Jewkes et al. [13]
