# Supplementary material for: The Long-Term Health Consequences of Child Physical Abuse, Emotional Abuse, and Neglect: A Systematic Review and Meta-Analysis
Source: PLoS Med. 2012 Nov 27;9(11):e1001349. doi: 10.1371/journal.pmed.1001349 (PMC3507962; doi:10.1371/journal.pmed.1001349)
Supplement: Table S9 — Obesity subgroup analyses. (DOC) [file pmed.1001349.s051.doc]

Table S9 Obesity subgroup analyses

|  | **No of data points** | **Pooled OR** | **95% LCI** | **95% UCI** | **Cochran's Q** | **I^2^** | **Test of heterogeneity**  **p-value** |
| --- | --- | --- | --- | --- | --- | --- | --- |
| **Primary analysis** |  |  |  |  |  |  |  |
| **Obesity** |  |  |  |  |  |  |  |
| Physical abuse | 11 | 1.32 | 1.06 | 1.64 | 37.54 | 73.36 | <0.01 |
| Emotional abuse | 5 | 1.24 | 1.13 | 1.36 | 6.95 | 42.48 | 0.14 |
| Neglect | 18 | 1.07 | 0.97 | 1.19 | 44.68 | 61.95 | <0.01 |
| **Subgroup analyses** |  |  |  |  |  |  |  |
| **1. Assessment of outcome** |  |  |  |  |  |  |  |
| ***Obesity (BMI measurement)*** |  |  |  |  |  |  |  |
| Physical abuse | 7 | 1.18 | 1.02 | 1.37 | 10.74 | 44.15 | 0.10 |
| Emotional abuse | 3 | 1.28 | 1.10 | 1.48 | 6.05 | 66.96 | 0.05 |
| Neglect | 5 | 1.31 | 0.90 | 1.92 | 19.14 | 79.10 | <0.01 |
| ***Obesity (waist circumference)*** |  |  |  |  |  |  |  |
| Physical abuse | 1 | 1.33 | 1.11 | 1.60 | not pooled | not pooled | not pooled |
| Emotional abuse | 2 | 1.18 | 1.05 | 1.33 | 0.00 | 0.00 | 0.94 |
| Neglect | 11 | 0.98 | 0.93 | 1.03 | 8.19 | 0.00 | 0.61 |
| ***Obesity (self-reported)*** |  |  |  |  |  |  |  |
| Physical abuse | 3 | 2.03 | 1.02 | 4.06 | 13.89 | 85.61 | <0.01 |
| Neglect | 2 | 5.33 | 2.32 | 12.25 | 0.16 | 0.00 | 0.69 |
| **2. Gender** |  |  |  |  |  |  |  |
| ***Female*** |  |  |  |  |  |  |  |
| Physical abuse | 3 | 1.47 | 0.87 | 2.46 | 8.14 | 75.44 | 0.02 |
| Neglect | 4 | 1.19 | 0.89 | 1.60 | 7.69 | 60.99 | 0.05 |
| ***Male*** |  |  |  |  |  |  |  |
| Physical Abuse | 2 | 1.07 | 0.81 | 1.42 | 0.87 | 0.00 | 0.35 |
| Emotional abuse | 1 | 1.52 | 1.17 | 1.87 | not pooled | not pooled | not pooled |
| Neglect | 3 | 0.93 | 0.79 | 1.09 | 1.83 | 0.00 | 0.40 |
| **3. Sample type** |  |  |  |  |  |  |  |
| ***Population based*** |  |  |  |  |  |  |  |
| Physical abuse | 8 | 1.41 | 1.07 | 1.84 | 27.29 | 74.35 | <0.01 |
| Emotional abuse | 2 | 1.18 | 1.05 | 1.33 | 0.00 | 0.00 | 0.94 |
| Neglect | 18 | 1.07 | 0.97 | 1.19 | 44.68 | 61.95 | <0.01 |
| ***Non-representative*** |  |  |  |  |  |  |  |
| Physical abuse | 3 | 1.21 | 1.03 | 1.43 | 7.49 | 73.31 | 0.02 |
| Emotional abuse | 3 | 1.28 | 1.10 | 1.48 | 6.05 | 66.96 | 0.05 |
| **4. Assessment of exposure** |  |  |  |  |  |  |  |
| ***Prospective*** |  |  |  |  |  |  |  |
| Physical abuse | 1 | 4.82 | 1.71 | 13.54 | not pooled | not pooled | not pooled |
| Neglect | 11 | 1.10 | 0.93 | 1.29 | 37.53 | 73.36 | <0.01 |
| ***Retrospective*** |  |  |  |  |  |  |  |
| Physical abuse | 10 | 1.23 | 1.04 | 1.46 | 30.68 | 70.66 | <0.01 |
| Emotional abuse | 5 | 1.24 | 1.13 | 1.36 | 6.95 | 42.48 | 0.14 |
| Neglect | 7 | 1.02 | 0.90 | 1.14 | 6.99 | 14.22 | 0.32 |
| **5. Dose-response relationship*** |  |  |  |  |  |  |  |
| Physical abuse sometimes | 1 | 1.18 | 1.07 | 1.32 | not pooled | not pooled | not pooled |
| Physical abuse often | 1 | 1.58 | 1.26 | 1.99 | not pooled | not pooled | not pooled |
| Emotional abuse sometimes | 1 | 1.16 | 1.05 | 1.30 | not pooled | not pooled | not pooled |
| Emotional abuse often | 1 | 1.36 | 1.19 | 1.52 | not pooled | not pooled | not pooled |

*Dose-response relationship data source: Williamson et al. [31]
