# Supplementary material for: The Long-Term Health Consequences of Child Physical Abuse, Emotional Abuse, and Neglect: A Systematic Review and Meta-Analysis
Source: PLoS Med. 2012 Nov 27;9(11):e1001349. doi: 10.1371/journal.pmed.1001349 (PMC3507962; doi:10.1371/journal.pmed.1001349)
Supplement: Table S10 — Tobacco smoking subgroup analyses. (DOC) [file pmed.1001349.s052.doc]

Table S10 Tobacco smoking subgroup analyses

|  | **No of data points** | **Pooled OR** | **95% LCI** | **95% UCI** | **Cochran's Q** | **I^2^** | **Test of heterogeneity**  **p-value** |
| --- | --- | --- | --- | --- | --- | --- | --- |
| **Primary Analysis** |  |  |  |  |  |  |  |
| **Tobacco Smoking** |  |  |  |  |  |  |  |
| Physical abuse | 19 | 1.55 | 1.09 | 2.21 | 161.75 | 88.87 | <0.01 |
| Emotional abuse | 6 | 1.70 | 1.55 | 1.87 | 2.38 | 0.00 | 0.79 |
| Neglect | 2 | 1.20 | 0.98 | 1.48 | 0.63 | 0.00 | 0.43 |
| **Subgroup analyses** |  |  |  |  |  |  |  |
| **1. Current smoker** |  |  |  |  |  |  |  |
| Physical abuse | 9 | 1.78 | 1.26 | 2.52 | 34.06 | 76.51 | <0.01 |
| Emotional abuse | 4 | 1.65 | 1.46 | 1.87 | 0.55 | 0.00 | 0.91 |
| Neglect | 2 | 1.20 | 0.98 | 1.48 | 0.63 | 0.00 | 0.43 |
| **Ever smoker** |  |  |  |  |  |  |  |
| Physical abuse | 10 | 1.45 | 0.83 | 2.55 | 104.44 | 91.38 | <0.01 |
| Emotional abuse | 2 | 1.79 | 1.55 | 2.06 | 0.98 | 0.00 | 0.32 |
| **2. Dose-response relationship*** |  |  |  |  |  |  |  |
| any Physical abuse | 1 | 1.28 | 1.13 | 1.45 | not pooled | not pooled | not pooled |
| 1-2 times Physical abuse | 1 | 1.15 | 0.99 | 1.35 | not pooled | not pooled | not pooled |
| 3-5 times Physical abuse | 1 | 1.40 | 1.11 | 1.78 | not pooled | not pooled | not pooled |
| 6+ times Physical abuse | 1 | 1.37 | 1.12 | 1.69 | not pooled | not pooled | not pooled |

*Dose-response relationship data source: Roberts et al. [32]
