# Supplementary material for: The Long-Term Health Consequences of Child Physical Abuse, Emotional Abuse, and Neglect: A Systematic Review and Meta-Analysis
Source: PLoS Med. 2012 Nov 27;9(11):e1001349. doi: 10.1371/journal.pmed.1001349 (PMC3507962; doi:10.1371/journal.pmed.1001349)
Supplement: Table S11 — Type 2 diabetes subgroup analyses. (DOC) [file pmed.1001349.s053.doc]

Table S11 Type 2 diabetes subgroup analyses

|  | **No of data points** | **Pooled OR** | **95% LCI** | **95% UCI** | **Cochran's Q** | **I^2^** | **Test of heterogeneity**  **p-value** |
| --- | --- | --- | --- | --- | --- | --- | --- |
| **Primary analysis** |  |  |  |  |  |  |  |
| **Type 2 diabetes** |  |  |  |  |  |  |  |
| Physical abuse | 11 | 1.01 | 0.79 | 1.29 | 41.26 | 75.76 | <0.01 |
| Emotional abuse | 3 | 1.19 | 0.74 | 1.93 | 10.45 | 80.86 | 0.01 |
| Neglect | 14 | 1.11 | 0.97 | 1.26 | 16.37 | 20.57 | 0.23 |
| **Subgroup analyses** |  |  |  |  |  |  |  |
| **1. Gender** |  |  |  |  |  |  |  |
| ***Female*** |  |  |  |  |  |  |  |
| Physical abuse | 5 | 0.98 | 0.85 | 1.12 | 5.66 | 29.39 | 0.23 |
| Neglect | 1 | 2.10 | 0.90 | 4.70 | not pooled | not pooled | not pooled |
| ***Male*** |  |  |  |  |  |  |  |
| Neglect | 1 | 2.60 | 1.00 | 7.10 | not pooled | not pooled | not pooled |
| Physical abuse | 1 | 0.70 | 0.30 | 1.80 | not pooled | not pooled | not pooled |
| **2. Assessment of exposure** | |  |  |  |  |  |  |
| ***Prospective*** |  |  |  |  |  |  |  |
| Neglect | 7 | 1.04 | 0.90 | 1.20 | 4.26 | 0.00 | 0.64 |
| ***Retrospective*** |  |  |  |  |  |  |  |
| Physical abuse | 11 | 1.01 | 0.79 | 1.29 | 41.26 | 75.76 | <0.01 |
| - Females | 5 | 0.94 | 0.75 | 1.16 | 5.83 | 31.34 | 0.21 |
| - Males | 1 | 1.52 | 1.16 | 2.00 | not pooled | not pooled | not pooled |
| Emotional abuse | 3 | 1.19 | 0.74 | 1.93 | 10.45 | 80.86 | 0.01 |
| Neglect | 7 | 1.30 | 1.00 | 1.69 | 10.71 | 44.00 | 0.10 |
| **3. Effect size expressed as Hazard ratio** |  |  |  |  |  |  |  |
| Physical abuse | 4 | 1.08 | 0.91 | 1.27 | 8.66 | 65.35 | 0.03 |
| **4. Dose response***  **(Hazard ratio)** |  |  |  |  |  |  |  |
| Physical abuse mild | 1 | 0.96 | 0.81 | 1.13 | not pooled | not pooled | not pooled |
| Physical abuse moderate | 1 | 1.07 | 0.93 | 1.23 | not pooled | not pooled | not pooled |
| Physical abuse severe | 1 | 0.97 | 0.77 | 1.23 | not pooled | not pooled | not pooled |

*Dose-response relationship data source: Rich-Edwards et al. [78]
