# Supplementary material for: The Long-Term Health Consequences of Child Physical Abuse, Emotional Abuse, and Neglect: A Systematic Review and Meta-Analysis
Source: PLoS Med. 2012 Nov 27;9(11):e1001349. doi: 10.1371/journal.pmed.1001349 (PMC3507962; doi:10.1371/journal.pmed.1001349)
Supplement: Table S12 — Evaluation of the evidence for a causal relationship within the Bradford Hill framework for prospective and retrospective studies. (DOC) [file pmed.1001349.s054.doc]

Table S12 Evaluation of the evidence for a causal relationship within the Bradford Hill framework for prospective and retrospective studies

| **Health outcome** | **Dose-response** | **Physical abuse** | | **Emotional abuse** | | **Neglect** | |
| --- | --- | --- | --- | --- | --- | --- | --- |
|  | **Relationship** | **Prospective*** | **Retrospective*** | **Prospective*** | **Retrospective*** | **Prospective*** | **Retrospective*** |
| Depressive disorders | yes (neglect, emotional) | strong | moderate | lacking | strong | weak | strong |
| Anxiety disorders | yes (physical) | strong | moderate | lacking | strong | moderate | moderate |
| Eating disorders | yes (physical abuse) | lacking | strong | lacking | weak | weak | weak |
| Childhood behavioural/conduct disorders | no | strong | strong | lacking | lacking | weak | weak |
| Alcohol problems | no | no effect | moderate | lacking | moderate | no effect | inconsistent |
| Drug use | yes (emotional) inconsistent (neglect) | moderate | strong | lacking | moderate | moderate | moderate |
| Suicide attempts/ ideation/self-inflicted injuries | yes (neglect) | weak (self-inflicted injuries only) | strong | lacking | strong | no effect (self-inflicted injuries only) | moderate |
| STIs/risky sexual behaviour | yes (physical and emotional abuse) | moderate | moderate | lacking | moderate | moderate | moderate |
| Type 2 diabetes | no | lacking | inconsistent | lacking | weak | no effect | moderate |
| Obesity | yes (physical and emotional abuse) | weak | moderate | lacking | moderate | inconsistent | inconsistent |
| Hypertension | yes | no effect | inconsistent | lacking | lacking | no effect | no effect |
| Current Smoking | yes (physical abuse) | lacking | moderate | lacking | moderate | lacking | inconsistent |

*Refers to ascertainment of exposure to child maltreatment.

The classification is adapted from Gilbert et al. [4]

Strong=consistent evidence from a number of studies of a significant strong effect (>=2) after adjustment for confounders;

Moderate= consistent evidence from a number of studies of a significant but small effect (<2), or of a stronger effect that is reduced after adjustment for confounders;

Weak=evidence from a few studies of a significant effect, or evidence of a significant effect based on poor quality studies or associations that do not persist after adjustment for confounders, or consistently seem to favour a positive effect;

Inconsistent=the effect is qualitatively different across studies (for example positive and negative associations or no association or association not statistically significant);

No effect=association not statistically significant

Lacking=no studies

Dose-response relationship observed: yes, no or inconsistent (different across studies)
