# Supplementary material for: The Long-Term Health Consequences of Child Physical Abuse, Emotional Abuse, and Neglect: A Systematic Review and Meta-Analysis
Source: PLoS Med. 2012 Nov 27;9(11):e1001349. doi: 10.1371/journal.pmed.1001349 (PMC3507962; doi:10.1371/journal.pmed.1001349)
Supplement: Text S2 — Review protocol. (DOC) [file pmed.1001349.s056.doc]

**Review protocol**

**The long-term health consequences of child physical abuse, emotional abuse and neglect: a systematic review and meta-analysis**

**Methods of the review**

The study will be conducted according to the PRISMA statement

**Data sources:** Three electronic databases (Medline, EMBASE, and PsycINFO) searched with the assistance of librarians.

Search terms:

| **Database** | **Search group** | **Search terms** |
| --- | --- | --- |
| **Embase** | Child maltreatment expanded search | (((maltreatment OR 'physical abuse' OR 'psychological abuse' OR 'dysfunctional household' OR 'dysfunctional households' OR 'child abuse'/exp OR 'battered child syndrome'/exp OR 'child neglect'/exp OR 'emotional deprivation'/exp OR 'dysfunctional family'/exp) AND (([child]/lim OR [adolescent]/lim) OR child*:ti,ab)) AND ('adulthood'/exp OR 'adult'/exp) AND (risk*:ab,ti OR harm*:ab,ti OR advers*:ab,ti OR associat*:ab,ti OR antecedent*:ab,ti OR predict*:ti,ab OR 'risk'/exp OR 'etiology'/exp OR 'longitudinal study'/exp OR 'case control study'/exp OR 'retrospective study'/exp)) NOT (sexual:ti)) |
| **Medline (Ovid)** | child maltreatment | 1. exp child abuse/  2. exp battered child syndrome/  3. exp psychosocial deprivation/  4. exp child of impaired parents/  5. ((physical* or emotional* or psychological*) adj abuse*).mp.  6. (dysfunctional adj (household* or famil*)).  7. ((neglect* or depriv*) adj child*).mp.  8. 1 or 2 or 3 or 4 or 5 or 6 or 7  9. exp child/  10. exp adolescent/  11. exp child,preschool/  12. child*.ti,ab.  13. 9 or 10 or 11 or 12 |
|  | adult | 14. exp adult/ |
|  | risk | 15. risk*.ab,ti.  16. harm*.ab,ti.  17. advers*.ab,ti.  18. associat*.ab,ti.  19. antecedent*.ab,ti.  20. predict*.ti,ab.  21. exp risk/  22. exp longitudinal studies/  23. exp case control studies/  24. exp retrospective studies/  25. 15 or 16 or 17 or 18 or 19 or 20 or 21 or 22 or 23 or 24 |
|  | Exclude child sexual abuse | 26. 8 and 13 and 14 and 25  27. (sexual*)).ti.  28. 26 not 27 |
| **PsycINFO** | Child maltreatment | Search Query #19 ((((DE=("child abuse" or "battered child syndrome" or "abandonment" or "child neglect")) or(KW=((emotional abuse) or (physical abuse)) and (child* or adolesc*))) and (adulthood)) and(KW=(risk or etiology or association) or PT=(retrospective or (case control) or longitudinal) or KW=(harm* or adverse or anteceden*))) not ((TI=("sexual abuse" or "sexually abused")) |

**Additional searching:**

Reference list review (any article pulled for possible inclusion)

Contact with authors

Any article deemed suitable by reviewers is included for closer examination.

**Inclusion/exclusion criteria**

**Inclusion criteria:**

Studies were included if they were published in a peer-reviewed journal and reported an association between exposure to child non-sexual maltreatment (child emotional abuse, child physical abuse and neglect in childhood) and health outcomes.

1. *Question of interest*: Are individuals who have experienced non-sexual child maltreatment at higher risk of developing health related problems (as defined by Global Burden of Disease and risk factors (GBD) study) compared with those who are not exposed?

*Population*: General population, children adolescents or adults. Non-representative samples were also considered (such as large military/HMO/school student/college samples) for consistency of relationships.

*Intervention/exposure*: Definition of child maltreatment should follow WHO definition (physical, psychological/emotional abuse and neglect but not combined abuse).

WHO Definition of child non-sexual maltreatment

| **Physical abuse**  Physical abuse of a child is defined as the intentional use of physical force against a child  that results in – or has a high likelihood of resulting in – harm for the child’s health, survival, development or dignity. This includes hitting, beating, kicking, shaking, biting, strangling, scalding, burning, poisoning and suffocating. Much physical violence against children in the home is inflicted with the object of punishing. |
| --- |
| **Emotional and psychological abuse**  Emotional and psychological abuse involves both isolated incidents, as well as a pattern of failure over time on the part of a parent or caregiver to provide a developmentally appropriate and supportive environment. Acts in this category may have a high probability of damaging the child’s physical or mental health, or its physical, mental, spiritual, moral or social development. Abuse of this type includes: the restriction of movement; patterns of belittling, blaming, threatening, frightening, discriminating against or ridiculing; and other non-physical forms of rejection or hostile treatment. |
| **Neglect**  Neglect includes both isolated incidents, as well as a pattern of failure over time on the part of a parent or other family member to provide for the development and well-being of the child – where the parent is in a position to do so – in one or more of the following areas:  • health;  • education;  • emotional development;  • nutrition;  • shelter and safe living conditions.  The parents of neglected children are not necessarily poor. |

*Exposure Measurement* – abuse or neglect substantiated by court system or self-report by victim or other screening instrument such as ICAST

Age range for exposure – 0-18 years but also accept studies where the definition of childhood is not stated (abuse occurs “in childhood”).

*Comparison*: Individuals not exposed to child maltreatment

*Outcome*: GBD health outcomes, GBD risk factors (including tobacco smoking, high body mass, high blood pressure and unsafe sex or risky sexual behavior but excluding intimate partner violence).

*Outcome Measurement* – Mental and physical health outcomes diagnosed by a health professional (using ICD, DSM criteria) or direct physical measurements and blood tests but standardised/non-standardised screening instruments or self-reported health outcomes also accepted (quality score adjusted accordingly).

2. *Study designs of interest*: Prospective and retrospective cohort, cross-sectional and case-control studies included.

No limits on year of publication or language.

Articles in LOTE deemed relevant based on its abstract are translated.

**Exclusion criteria:**

Articles initially excluded if they are duplicates or if the title clearly demonstrates that the exposure and outcome of interest are not the focus of the article. Articles are then excluded based on the following:

- The article does not explore an association between child non-sexual maltreatment and health outcomes.
- No effect size and uncertainty information reported or cannot be computed from information given.
- Studies do not provide result by individual abuse categories (just combined abuse category).
- The article does not focus on child physical abuse, emotional abuse or neglect (focus is victimization, bullying or sexual violence/abuse in adulthood).
- Non-sexual child maltreatment is considered as a risk factor/mediator between two other exposure and outcome variables.
- Outcome is not a GBD health outcome including: gambling problems, multiple health problems (not defined); self rated health (poor/fair); revictimization, child abuse as a risk factor for adolescent relationship violence, dating violence, domestic physical abuse, sexual victimization, criminal behaviour/delinquency/aggressive behaviour/violent behaviour (except childhood behavioural disorders such as ADHD and conduct disorders), non-ICD health outcomes (such as neuroticism/ impulsiveness/teenage pregnancy/lifetime perpetration of IPV), non-spinal pain such as pelvic pain, sexual dysfunction, irritable bowel syndrome/chronic fatigue/pathological gambling).
- Focus of article is ‘other childhood trauma’ or other adverse childhood experience and is not consistent with WHO definition of non-sexual childhood maltreatment including death of a parent/sibling, substance abuse by parent/mentally disordered parent, frequent exposure to violence (collective and interpersonal, witnessing intimate partner violence), economic deprivation, parental attachment style/rearing patterns/ bonding/ rejection/discipline practices/deprivation; divorce/abandonment, family dysfunction/discord, victim of childhood bullying, quality of childhood caretaking.
- Focus of article is assessing the validity of a child maltreatment scale.
- The focus of the article is on risk factors for child maltreatment.
- There is no control group or comparison group. The study employs a clinical sample without an appropriate comparison group.
- The study is a review article.
- Multiple papers on same study population excluded unless different outcomes are presented.

Study inclusion/exclusion is completed independently (RN and MB). Results are reviewed (RN) and any disagreement is recorded. Results are discussed (RN and MB) to reach consensus.

**Data extraction sheet**

The data extraction sheet is first pilot tested on 10 studies and then revised accordingly to include:

**Identification of study:**

1. Record the first authors’ last name, initials
2. Record the journal name
3. Record the year of publication
4. Record the volume number
5. Record the page numbers

# Characteristics of study:

1. Study period
2. Study design
3. Sample size and % female
4. Retrospective/prospective analysis
5. Type of abuse
6. Assessment of exposure
7. Health outcomes
8. Assessment of outcome

**Other data**:

1. Effect size and 95% confidence interval.

If epidemiological studies report the effect size as a relative risk this is converted to odds ratio, where possible, using the OR2RR utility. OR2RR is an Excel add-in which implements a method to convert odds ratios into relative risks and the other way around, using the disease risk and risk factor prevalence reported in the study. For the conversion of relative risks into odds ratios an analytical expression is used (Barendregt J, OR2RR. Brisbane: EpiGear International Pty Ltd, [http://www.epigear.com](http://www.epigear.com/)).

**Quality Assessment:**

| **Representativeness of the population**   - Population based representative=1 - Not representative/selected group/volunteers/no description=0 |
| --- |
| **Ascertainment of exposure to child abuse and neglect**   - Data on child maltreatment collected prospectively=1 - Data on child maltreatment collected retrospectively=0 |
| **Selection of the non-exposed cohort/controls**   - Drawn from the same population=1 - Drawn from a different source/no description=0 |
| **Assessment of childhood abuse and neglect**   - Secure official record (court substantiated abuse)=1 - Self-reported or structured interview or self administered questionnaire or no description=0 |
| **Case definition for childhood abuse**   - Uses WHO definitions of child maltreatment or court substantiated abuse or Barnett-Cicchetti Maltreatment Classification System =1 - Marks and bruises (physical abuse), questions from scales (Childhood Trauma Questionnaire) or published surveys or own system=0 |
| **Assessment of outcome**   - Use of a structured diagnostic interview for DSM-III/IV (DIS, DISC, CIDI) (mental health), direct physical measurements, blood tests (physical health)=1 - Questions from published health surveys/screening instruments or own system /symptoms described/no system/not specified/self-reported = 0 |
| **Adequacy of follow-up of cohorts (where relevant) or response rate**   - Completeness (>=80%) with description of those lost to follow up =1 - Poor (<80%) or no statement=0 |
| **Appropriate statistical analysis**   - Yes=1 - No=0 |
| **Appropriate methods to control confounding**   - Yes=1 (multivariable adjusted OR including SES, education or family dysfunction in models) - No=0 (univariate analysis or controls for age/sex only) |
| **Source of funding declared**   - Yes (financial disclosure, funding/support/grant declared) =1 - No = 0 |

The total quality score for each study is the sum of the scores for individual assessment items. This is converted to a proportional quality score for use in Meta-XL (the total quality score divided by the maximum score possible).

Data extraction and quality assessment is completed independently (RN and MB). Results are reviewed (RN) and where disagreement occurs results are discussed (RN and MB and TV) to reach consensus.
